# Physical activity and cardiometabolic health across an extreme lifestyle gradient

**DOI:** 10.1093/emph/eoag017

**Published:** 2026-07-21

**Authors:** Thomas S Kraft, Ian J Wallace, Yvonne A L Lim, Kar Lye Tam, Tan Bee Ting A/P Tan Boon Huat, Steven K W Chow, Kamal Solhaimi bin Fadzil, Colin Nicholas, Izandis bin Mohd Sayed, Vivek V Venkataraman, Amanda J Lea

**Affiliations:** Department of Anthropology, University of Utah, Salt Lake City, UT 84112, USA; Department of Anthropology, University of New Mexico, Albuquerque, NM 87131, USA; Department of Parasitology, Faculty of Medicine, Universiti Malaya, Kuala Lumpur 50603, Malaysia; Department of Parasitology, Faculty of Medicine, Universiti Malaya, Kuala Lumpur 50603, Malaysia; Department of Parasitology, Faculty of Medicine, Universiti Malaya, Kuala Lumpur 50603, Malaysia; Federation of Private Medical Practitioners’ Associations of Malaysia, Kuala Lumpur, Malaysia; Pantai Hospital, Kuala Lumpur, Malaysia; Department of Anthropology and Sociology, Universiti Malaya, Kuala Lumpur, 50603, Malaysia; Center for Orang Asli Concerns, Subang Jaya, Malaysia; Hospital Orang Asli, Gombak, Kuala Lumpur, Malaysia; Department of Anthropology and Archaeology, University of Calgary, Calgary, AB T3B 5C1, Canada; Department of Biological Sciences, Vanderbilt University, Nashville, TN 37232, USA; Evolutionary Studies Initiative, Vanderbilt University, Nashville, TN 37232, USA; Vanderbilt Genetics Institute, Vanderbilt University, Nashville, TN 37232, USA

**Keywords:** physical activity, obesity, cardiometabolic health, market integration, Orang Asli

## Abstract

**Background and objectives:**

Cardiometabolic health in many small-scale subsistence populations has been shown to be substantially better than populations living in industrialized, urban environments. This disparity is often partially attributed to the characteristically high levels of physical activity observed in non-industrialized societies, yet it remains unclear how much of the difference in cardiometabolic health between non-industrialized and industrialized populations is due specifically to physical activity rather than to other lifestyle factors that covary with industrialization.

**Methodology:**

To address this question, we collected objective data on physical activity (as characterized by daily step counts, time spent inactive, and intensity gradient, among other measures), cardiometabolic biomarker profiles (16 measures: anthropometrics, blood lipids, blood pressure, obesity, hypertension, pre-diabetes, and diabetes), and detailed lifestyle information from 1075 Orang Asli adults in Peninsular Malaysia, who span an exceptionally wide lifestyle gradient from small-scale subsistence communities to urban, industrialized settings.

**Results:**

More urban and market-integrated lifestyles were associated with marked reductions in physical activity, particularly among older individuals, and with poorer cardiometabolic health. At the individual level, greater physical activity was directly associated with better cardiometabolic health. However, physical activity accounted for only a small portion of urbanization’s association with cardiometabolic health, indicating that other industrialization-related factors play substantial roles in shaping health outcomes.

**Conclusions and implications:**

These findings suggest that declining physical activity represents an evolutionarily novel challenge that exacerbates the burden of cardiometabolic disease, especially among older adults, in contemporary industrialized environments.

## INTRODUCTION

The generally robust cardiometabolic health observed among many non-industrial, small-scale subsistence societies stands in stark contrast to the escalating prevalence of obesity, type 2 diabetes, and cardiovascular disease in industrialized, urban populations [1]. These health disorders are notably rare or absent among many hunter-gatherer, pastoralist, and subsistence farming groups, suggesting that elements of non-industrialized lifestyles help preserve cardiometabolic health [2–7]. Given that physical activity levels among hunter-gatherers, pastoralists, and subsistence farmers are typically much higher than those of people in many industrialized nations [5], and extensive evidence from industrialized societies that routine exercise promotes cardiometabolic health [8–10], it is commonly hypothesized that health differences between non-industrialized and industrialized societies are strongly driven by variation in physical activity levels [3, 5, 6, 11]. However, it remains uncertain how much of the observed differences in cardiometabolic health between non-industrialized and industrialized societies are directly attributable to physical activity, as opposed to myriad other lifestyle factors that also change with industrialization.

High levels of physical activity among small-scale subsistence societies undoubtedly contribute to better cardiometabolic health; there is no reason to expect that exercise would be beneficial only in industrialized settings. Yet, multiple lines of evidence suggest that the magnitude of this benefit could be smaller than commonly assumed. First, studies using doubly labeled water have revealed similar levels of total daily energy expenditure between non-industrialized and industrialized populations, suggesting that differences in the risk of obesity and obesity-related disorders may stem more from variation in dietary energy intake than differences in physical activity [12, 13]. Second, studies of certain non-industrial societies have identified deterioration in cardiometabolic health among traditional-living people who have recently transitioned to more urban, industrialized environments, despite the absence of concomitant major declines in their physical activity levels [14–16]. Third, although people in small-scale subsistence societies are generally very active, their movement patterns consist primarily of light-to-moderate intensity activities such as walking and food processing [17, 18]. Although light- and moderate-intensity activities have been shown to provide some cardiometabolic benefits in industrialized contexts, their effects are inconsistent and often smaller than the benefits of vigorous exercise [8, 19]. For example, a recent analysis of UK Biobank data found that one minute of vigorous physical activity conferred the same reduction in type 2 diabetes risk as approximately nine minutes of moderate activity or ninety-four minutes of light activity [20].

The degree to which high physical activity levels in small-scale subsistence societies explain their favorable cardiometabolic health remains uncertain largely because there have been few studies of sufficient sample size directly linking objective measures of physical activity with cardiometabolic health across non-industrialized and industrialized contexts [5, 14]. To address this gap, we investigated cross-sectional relationships between physical activity, cardiometabolic health biomarkers, and lifestyle variation among the Indigenous peoples of Peninsular Malaysia known as the Orang Asli. The Orang Asli are traditionally rainforest-dwelling hunter-gatherers, fishers, and subsistence farmers. However, in recent years, many individuals have experienced varying degrees of lifestyle change due to factors such as acculturation, national economic development, the expansion of built infrastructure, and integration into the market economy, with the result that Orang Asli lifestyles now span a wide spectrum from traditional, non-industrialized to urban, industrialized. Previously, we have introduced an index of urbanicity that quantifies this broad lifestyle variation, and have shown that cardiometabolic health varies in a predictable way across the lifestyle spectrum, with the best health observed among the most traditional and least urbanized Orang Asli [21].

In this study, we collected data from 1075 Orang Asli adults spanning an extreme lifestyle gradient to test three general hypotheses about the relationships among urbanization, physical activity, and cardiometabolic health. First, we combined detailed ethnographic data on lifestyle variation with gold-standard accelerometry to characterize physical activity patterns in a traditionally non-industrialized population undergoing rapid lifestyle change, and to test the hypothesis that greater urbanization is associated with decreased levels of physical activity. We evaluated multiple metrics and dimensions of physical activity (including differences in the effects of inactivity versus light, moderate, or vigorous activity), and investigated age-related variation given that susceptibility to many cardiometabolic disorders increases with age [22]. Second, we collected high-dimensional cardiometabolic health biomarker data to test the hypothesis that higher physical activity levels are associated with better health outcomes across the lifestyle spectrum at the individual level—that is, to confirm that Orang Asli across the full range of urbanization exhibit the same positive relationship between physical activity and cardiometabolic health observed in many industrialized populations. Third, we estimated the degree to which physical activity mediates relationships between urbanization and cardiometabolic health, to assess the relative contribution of physical activity versus other lifestyle changes accompanying industrialization.

## METHODOLOGY

### Study population

The Orang Asli, or “original people,” comprise 19 distinct ethnolinguistic groups that collectively represent the Indigenous peoples of Peninsular Malaysia. Data for this study come from 34 villages that are predominantly occupied by the following groups: Batek, Batek Nogn, Jahai, Jahut, Jakun, Kensiu, Semai, Semaq Beri, Temiar, and Temuan. These groups have long been separated by anthropologists into three larger groups—Senoi, Proto Malay, and Negrito—based primarily on language and subsistence type, as well as phenotypic differences [23, 24].

A notable feature of the Orang Asli environment is the dramatic lifestyle gradient that exists today, ranging from semi-nomadic practitioners of foraging lifestyles to fully sedentarized groups living in urban or peri-urban centers who are fully engaged with the national market economy [25]. The lifestyle gradient is largely a result of Malaysia’s recent history of rapid economic development; prior to the 1970’s and 1980’s, the interior regions of Peninsular Malaysia remained relatively inaccessible with little built infrastructure (with notable exceptions in areas developed for mining operations, mainly gold and tin). After this time, however, major development projects were started to increase logging and mining, in addition to the expansion of large-scale oil palm and rubber agriculture. The success of these industries, combined with substantial petroleum revenues, has propelled Malaysia in the past 50 years through an extremely rapid transition toward “developed” status. Orang Asli have remained relatively marginalized throughout this process, with a high proportion currently living in poverty, but the extent of economic development remains highly variable across communities and is heavily dependent on geographical proximity to major population centers. Additional information about the study population is provided in the Supplementary Materials and Methods.

### Study design

This study is part of the *Orang Asli Health and Lifeways Project (OA HeLP)*, a long-term study of behavior, culture, environment, and health among Orang Asli throughout Peninsular Malaysia [25]. Following consent at the community and individual levels, data are collected from adults during mobile health clinics that travel to study communities to provide free healthcare. Participants are required to be 18 years of age or older to participate, and no other exclusion criteria are applied to encourage representative sampling. In addition to providing medical treatment, a team of doctors, assistants, and anthropologists conduct lifestyle interviews and comprehensive health screenings, including analysis of blood-based biomarkers from non-fasting venous blood draws using point-of-care devices (CardioChek Plus for blood lipids, A1Cnow + for HbA1c, and AccuChek for blood glucose). Sixteen standard cardiometabolic phenotypes were measured or derived for participants in this study as described in the Supplementary Materials and Methods. Sample characteristics are described in Table S1. All data in this study were collected between 2020 and 2024.

Detailed questionaries about salient lifestyle factors were used to assign each individual an urbanicity score, as described in Watowich *et al.* [21] and Table S2. The score is based on established approaches [26], and integrates a wide range of community-level attributes, including population density, household access to electricity and sanitation systems, infrastructure for community access, prevalence of wage labor, household ownership of televisions and mobile phones, and rates of formal education. This score has been demonstrated to effectively capture lifestyle variation in multiple non-industrial populations undergoing rapid change [21], and to predict cardiometabolic health in the Orang Asli specifically. This composite score quantifies a continuous spectrum of lifestyle variation, offering a more nuanced approach than dichotomies of “urban” versus “rural” [e.g. 27]. Importantly, not all components of the urbanicity score are expected to directly influence physical activity or cardiometabolic health. Instead, urbanicity serves as a proxy for a broader suite of lifestyle factors that might affect health through multiple, interconnected pathways, including shifts in physical activity.

### Accelerometry

At the end of research clinics, all participants were invited to wear research-grade accelerometers (Axivity AX3) on the non-dominant wrist for a period of one week, after which time the devices were collected and returned to researchers. Accelerometry is restricted to periods outside of research clinics to ensure that behavior is not altered by the presence of the research team. Accelerometers are configured to record information at a sampling frequency of 100 Hz with a dynamic range of 8 *g*. We focused data collection on the non-dominant wrist for comparability with other ongoing studies, such as recent waves of the US National Health and Nutrition Examination Survey. Raw accelerometry files were downloaded and pre-processed using standard workflows in the *GGIR* package (v3.2.6) in R as described in the Supplementary Materials and Methods. We extracted eight summary metrics in order to capture distinct aspects of physical activity (Table 1). These included two metrics for overall physical activity [Euclidean Norm Minus One (ENMO) and daily step counts], five metrics capturing the intensity of activity [time spent inactive or in light, moderate, or vigorous activity, and intensity gradient (IG)], and a complexity-based measure of self-similarity characterizing patterns of physical activity over time that has been previously demonstrated to strongly predict mortality [28].

**Table 1:** Physical activity metrics employed in this study.

Variable	Description
*Euclidean Norm Minus One (ENMO)*	Euclidean norm (vector magnitude) of the x, y, and z axes (for a tri-axial device) representing raw signals of acceleration, accounting for the effect of gravity by subtracting one gravitational unit.
*Daily step count*	Estimation of the number of steps an individual takes on a given day, calculated using the Verisense (“revised” or version 2) algorithm [67].
*Minutes of inactivity*	Number of daily waking minutes spent inactive, calculated as time spent below the light-activity threshold (ENMO <30 m*g*).
*Minutes of light activity*	Number of daily minutes spent in light physical activity, defined using a threshold of 100 > ENMO ≥30 m*g*.
*Minutes of moderate activity*	Number of daily minutes spent in moderate physical activity, defined using a threshold of 400 > ENMO ≥100 m*g*.
*Minutes of vigorous activity*	Number of daily minutes spent in vigorous physical activity, defined using a threshold of ENMO ≥400 m*g*.
*Intensity gradient (IG)*	The IG is a continuous (cut-point free) variable that represents the amount of time that a person spends across all intensity levels [68, 69]. It is measured as a slope value, derived from a regression on binned acceleration data of ln-transformed time spent against ln-transformed acceleration intensity. A more negative (lower) gradient reflects a steeper drop with little time accumulated at moderate and higher intensities. In contrast, a less negative (higher) gradient reflects a shallower drop with relatively more time spent at moderate and higher intensities.
*Fractal complexity*	Calculated using detrended fluctuation analysis (DFA), fractal complexity is a measure of signal self-similarity (or self-affinity) across a time-series by analyzing correlations between the amplitude of signal fluctuations at different time-scales. Self-similarity describes a signal which, like a geometric fractal, has short subcomponents that contain the same complexity as increasingly longer components. The metric used here, **a**, is a slope measurement from the relationship between acceleration fluctuation amplitudes and different time-scales of measurement. If **a** is closer to 0.5, this represents less self-affinity and greater randomness, whereas a value of 1.0 represents a high degree of self-similarity (values >1.0 represent even higher regularity) [70].

### Statistical analyses

Associations between age and physical activity outcomes were estimated using Bayesian generalized additive mixed models with age terms modeled using thin-plate splines and random intercepts for repeat observations of individuals (each row of data represented a person-day for a given physical activity metric). To assess associations between urbanicity and physical activity, we fit Bayesian multilevel models of physical activity variables (standardized, with inactivity and step counts first square-root transformed) as a function of age (thin-plate spline), sex, urbanicity, and the interaction of age and urbanicity with a random intercept for individual. Given that there are many ways to conceptualize urbanization and lifestyle change, we also repeated analyses using other previously developed lifestyle metrics [21]. Models were fit using the *brms* package in R using weakly informative priors. Standard diagnostics indicated no problems during estimation (R^ ≅ 1, good mixing, and sufficient effective sample sizes).

We analyzed associations between physical activity and cardiometabolic health outcomes at the person-level by first collapsing physical activity metrics across days into the average value, and standardizing [subtracting the mean and dividing by the standard deviation (SD)] continuous variables for comparison. We then ran a series of linear models regressing the cardiometabolic outcomes on either urbanicity or physical activity metrics, separately, adjusting for an age × sex interaction and smoking (binary variable set to 1 if an individual reported smoking either industrially-produced cigarettes or loose-leaf tobacco “everyday” or “sometimes”). Model weights were applied based on the number of valid days of physical activity measurements per individual. Sample sizes are provided alongside full model results in Tables S3–S5.

Lastly, we conducted mediation analyses to examine how empirical estimates of physical activity statistically accounted for relationships between urbanicity and cardiometabolic health using Bayesian posterior g-computation in R [29]. Because the assumptions required for formal causal mediation are unlikely to be fully met in this observational setting, results were interpreted descriptively rather than causally. The mediation analyses involved fitting multiple models, one estimating physical activity as a function of urbanicity and another with cardiometabolic health outcomes as a function of both physical activity and urbanicity scores. All models included additional main effects for age and sex, an age by sex interaction, and smoking covariates, and were weighted according to the number of valid days of physical activity per individual; robustness checks were done by fitting these same models with alternative urbanicity metrics. For each mediation pathway, posterior draws from these models were used to simulate counterfactual mediator and outcome values under contrasting levels of urbanicity. These posterior simulations were then used to estimate the average causal mediation effect, average direct effect, total effect, and the proportion of the total urbanicity effect mediated by each physical activity metric. Posterior means, 95% credible intervals, and posterior probabilities in the direction of the main effect were used to summarize these outcomes. Mediation tests were only performed if prior analyses indicated strong evidence of a relationship (posterior probability of direction >0.975) between urbanicity and a given cardiometabolic outcome.

All analyses were conducted in the R statistical programming environment version 4.5.0 [30]. All model outputs are provided in Tables S3–S6.

### Ethical approvals

This research was approved by the Medical Review and Ethics Committee of the Malaysian Ministry of Health (protocol ID: NMRR-20-2214-55 565), the Malaysian Department of Orang Asli Development (permit ID: JAKOA.PP.30.052 JLD 21 (98)), and the Institutional Review Boards of Vanderbilt University (protocol ID: 212175), University of New Mexico (protocol ID: 14420), and University of Calgary (REB21–0432). Informed written consent was provided by all individuals at the time of data collection.

## RESULTS

**Figure 1:**
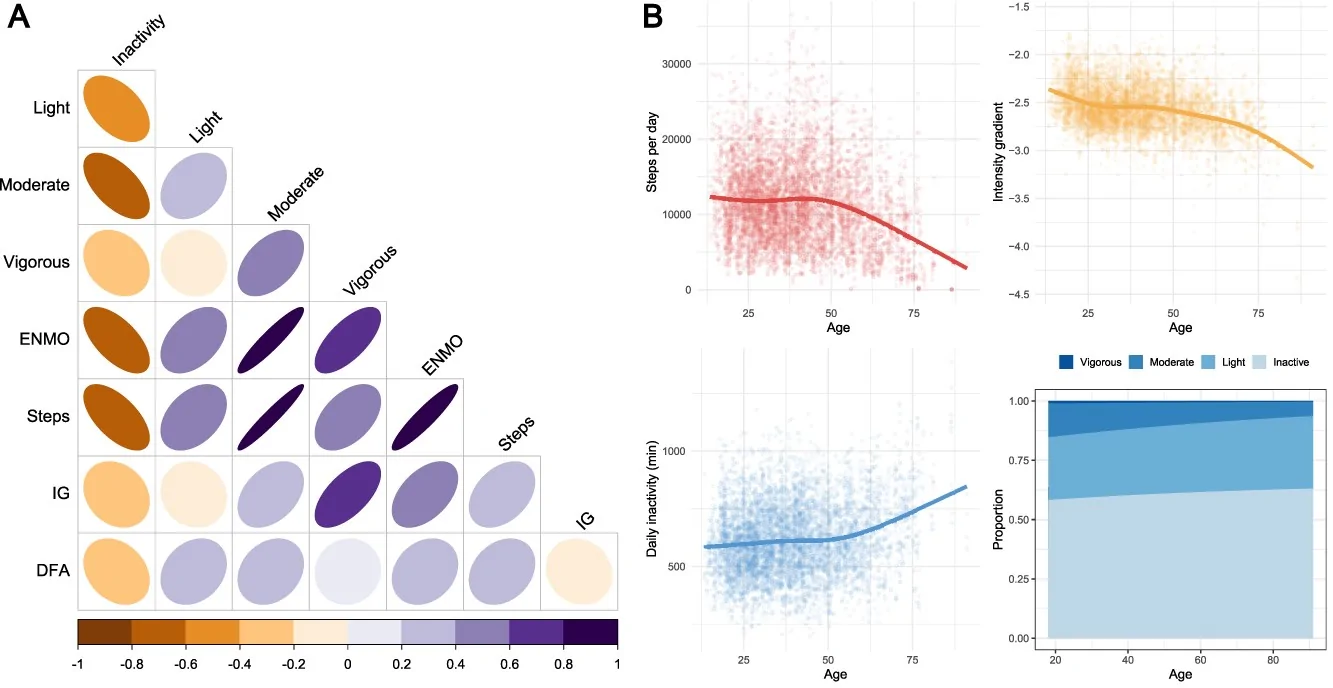
Physical activity and health outcomes in this study. (A) Correlations between different physical activity metrics extracted from accelerometry data (all correlations are at the daily level except for fractal complexity [DFA], which is a person-level metric). (B) Relationships of selected physical activity metrics with age. Smoothing curves are from generalized additive mixed models of physical activity outcomes as a function of a thin-plate spline for age and a random intercept for repeat observations of individuals (each observation is a single day of physical activity). Proportions of time spent in inactivity or vigorous/moderate/light activity as a function of age are derived from a multilevel Dirichlet regression with a random intercept for repeat observations of individuals.

Following quality control checks, physical activity information was available from 1075 participants (age range: 18–91; 63.6% female) for a total of 5892 valid days of activity (median = 6 days per person). We observed generally high correlations among the eight standard physical activity metrics (absolute average Pearson’s *r* = 0.42, range = 0.03–0.96), with some variables (e.g. time spent in moderate activity) showing high redundancy and others (e.g. intensity gradient) evincing only modest correlation to other metrics (Fig. 1A). As expected, time spent inactive was negatively correlated with other metrics, for which higher values represent greater amounts of activity. Based on these correlations, we identified three relatively independent metrics, representing theoretically different aspects of physical activity, as the focus of our main analysis: daily step counts (steps/day), time spent inactive (minutes/day), and intensity gradient (slope) (Fig. 1; analyses of the full suite of metrics are included in Supplementary Materials). Whereas step counts provide a general metric of overall accumulated activity (overlapping with ENMO and moderate activity), time spent inactive measures sedentary behavior, while intensity gradient captures engagement in more vigorous activities (overlapping with time spent in vigorous activity) (Table 1). All three measures included in the main analysis have been previously demonstrated to have important relationships to cardiometabolic health [31, 32].

### Physical activity is lower in more urban contexts

**Figure 2:**
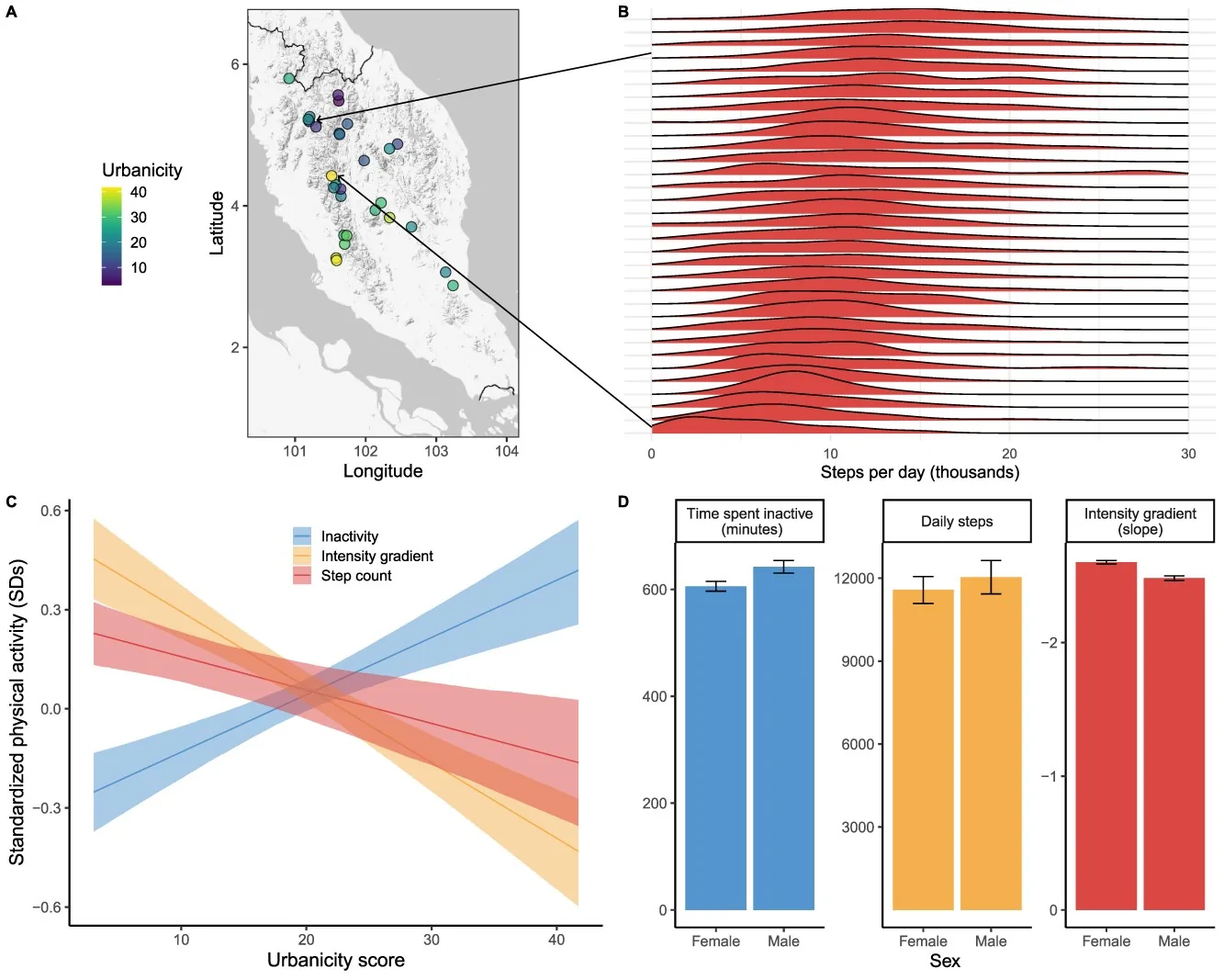
The effect of urbanicity on physical activity. (A) Map of peninsular Malaysia showing the approximate locations of study villages, colored by the community-level urbanicity score (lighter and darker colors represent more urban and rural environments, respectively). (B) Density plots of physical activity (number of estimated steps per day) by village. Arrows show the location of two examples with very different levels of physical activity. (C) Predicted levels of physical activity (standardized) as a function of urbanicity. Lines and shading show mean predictions and 95% confidence intervals from multilevel model of physical activity outcomes as a function of age (spline), sex, urbanicity, and age x urbanicity interaction, with a random intercept for individual (predictions for an individual at age 25 averaged across sexes). (D) Comparison of physical activity metrics between the sexes. Bars represent mean predicted response from posterior distributions ± 95% CIs. Note that the y-axis of the intensity gradient panel has been flipped such that more negative values are higher (a less negative value is indicative of more high intensity activity).

We found that physical activity metrics varied substantially across the extreme urbanicity gradient, ranging from Orang Asli communities with median daily step counts of ~5000 to ~15,000 (Fig. 2A-B). Notably, substantial within-community variation was also observed (Fig. 2B), similar to patterns typically seen in industrialized populations [33, 34]. In support of our first hypothesis—that greater urbanization would be associated with decreased levels of physical activity—we found that more urbanized individuals exhibited significantly lower average step counts and intensity gradients, and greater time spent inactive (Fig. 2C). Differences between the most and least urbanized individuals ranged from ~0.75 SD for intensity gradient and time spent inactive, to ~0.3 SD for daily step counts. The effects of urbanicity on intensity gradient and time spent inactive were thus stronger than the observed trends for daily step counts, suggesting that urbanization is especially discouraging of higher intensity activities, and that high-intensity activities are generally replaced with inactivity. Male participants exhibited greater daily step counts and intensity gradient despite spending more time inactive, reflecting higher levels of moderate and vigorous physical activity (Fig. 2D; ꞵ_sex_ [95% CI] = 0.08 [−0.01, 0.18], 0.49 [0.40, 0.58], 0.23 [0.13, 0.32] in models of standardized step counts, intensity gradient, and time spent inactive, respectively).

### Reductions in physical activity in urban environments are driven by less activity at later ages and less low- and moderate-intensity activity

Physical activity tends to decrease with age across essentially all human populations [17, 35], and the Orang Asli are no exception; steps per day, intensity gradient, and time spent in moderate or vigorous activity were all lower among older individuals, whereas time spent inactive or in light activity was higher (Fig. 1B). Average physical activity estimates remained relatively constant throughout adulthood, before beginning a rapid downward trend in individuals aged ~50–55 years (Fig. 1B).

**Figure 3:**
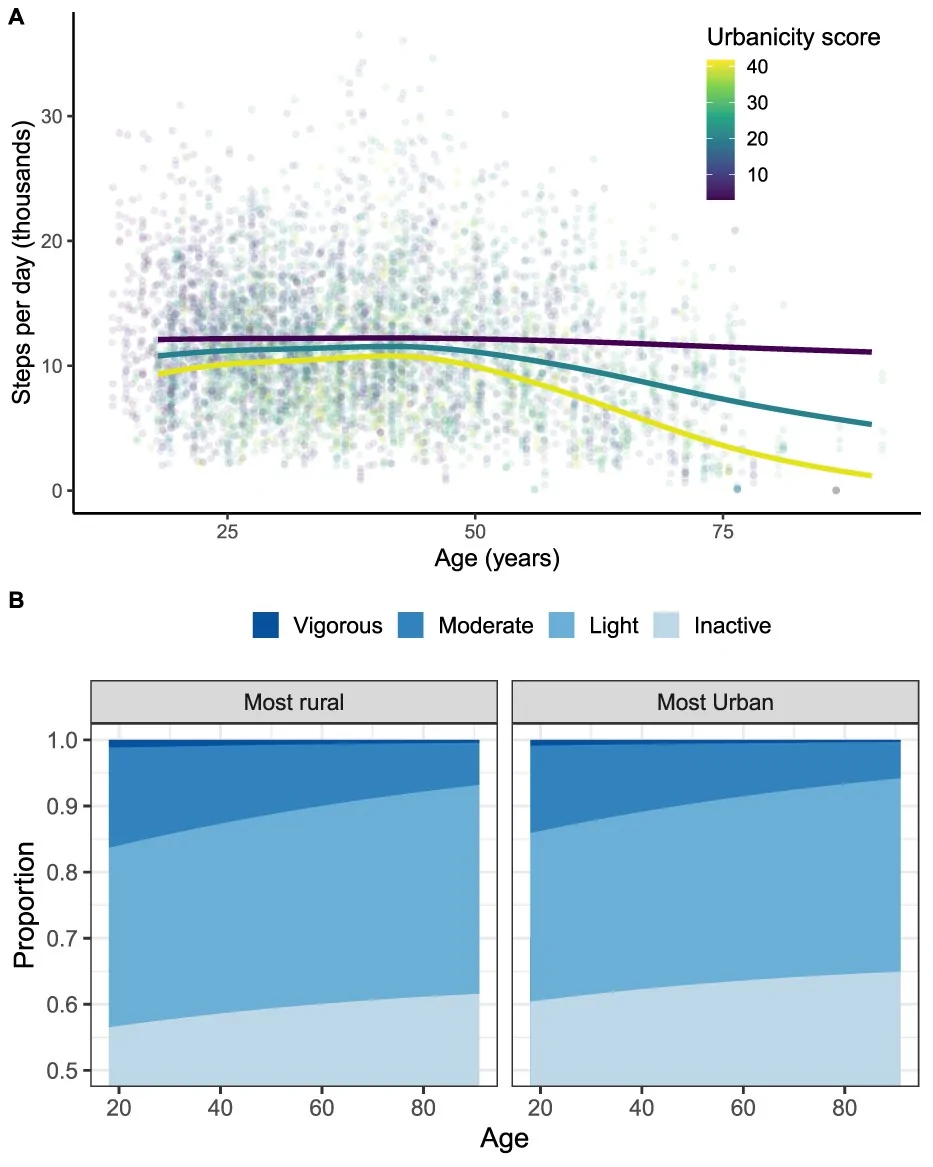
(A) Age-related decreases in physical activity begin around age 50 and are steeper in more urban environments. Each point is a person-day, colored by urbanicity score (yellow = more urban, purple = less urban), with lines representing model predictions from generalized additive mixed models of daily step counts as a function of an interaction between age (as a thin-plate spline) and urbanicity, adjusting for sex and a random intercept for repeat observations of individuals. Step counts were square-root transformed to improve normality and model fitting (daily step counts cannot be negative), with model predictions back-transformed for plotting. (B) The effects of urbanicity on time spent in different intensities of physical activity. Mean predicted proportions of time for individuals in the least and most urban environments, generated from a multilevel Dirichlet regression with fixed effects of age, sex, and urbanicity score plus a random intercept for repeat observations of individuals. Note that the y-axis starts at 0.5 to bolster visualization of differences (all areas between 0 and 0.5 are inactivity).

Interestingly, however, the age-associated decline after age 50 is heavily dependent on lifestyle. By age 75, fitted models show steep reductions in physical activity to an average of ~3600 steps/day (66% difference from peak) in the most urban locations, versus ~11 500 steps/day (5.8% difference from peak) in the least urban locations (Fig. 3A). Likewise, breakdowns of time spent in different intensities of physical activity reveal that urbanization promotes a shift from generally high levels of low- and moderate-intensity physical activity to greater time spent inactive (Fig. 3B; ꞵ_urbanicity_ [95% CI] from Dirichlet regression relative to inactivity as baseline: ꞵ_vigorous_ = −0.09 [−0.12, −0.05], ꞵ_moderate_ = −0.05 [−0.09, −0.01], ꞵ_light_ = −0.03 [−0.06, −0.01]). Vigorous physical activity is rare across the urbanization spectrum. Thus, the effects of urbanization on physical activity are age-dependent and predominantly driven by less time spent in low- and moderate-, rather than high-intensity, activities.

### Urbanization and reduced physical activity levels are associated with worse cardiometabolic health

**Figure 4:**
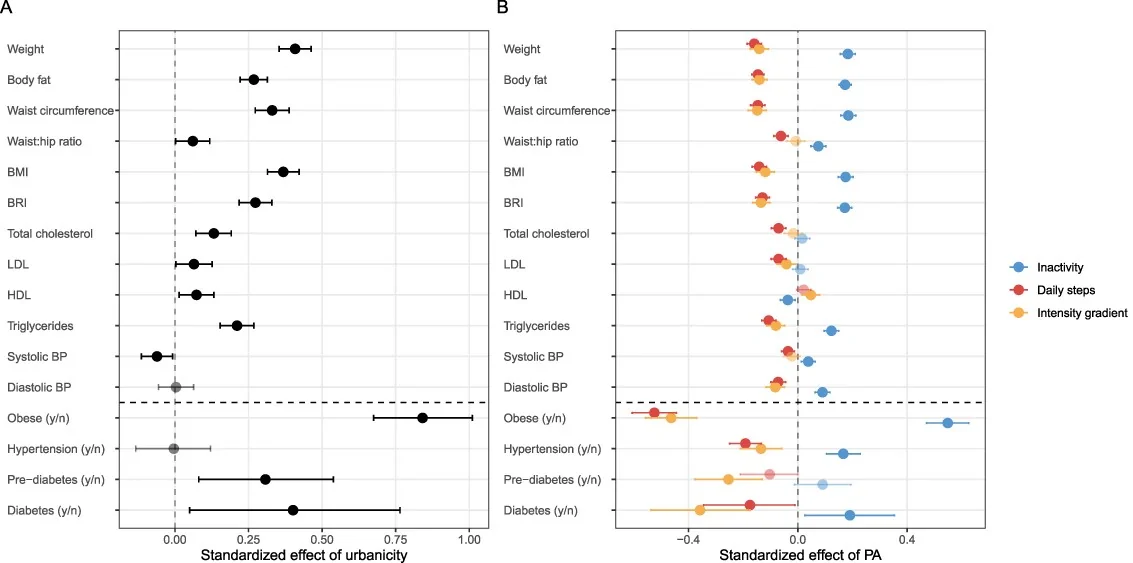
The effects of (A) urbanicity and (B) physical activity on cardiometabolic health outcomes. Points and error bars represent standardized effect size estimates (in units of SDs) and 95% CIs from models of average physical activity metrics per day, adjusting for age, sex, and an age × sex interaction (and smoking in panel B). Estimates below the horizontal dashed line represent binary outcomes, which were not standardized for analysis. Transparency indicates non-significant effects (probability of direction <0.975) and colors in (B) show outcomes for three selected physical activity metrics.

Consistent with our previous work [21], we found that cardiometabolic health among the Orang Asli was significantly worse in more urban environments. Specifically, weight, percent body fat, waist circumference, waist:hip ratio, body mass index (BMI), body roundness index (BRI), obesity, blood lipids (total cholesterol, low-density lipoprotein (LDL), triglycerides), and rates of pre-diabetes and diabetes all increased significantly as a function of urbanicity score (Fig. 4A). We also found that metrics indicating greater levels of physical activity were associated with better cardiometabolic health across many of the same outcome variables (Fig. 4B), despite some correlations among these traits (Fig. S1). For example, step counts, intensity gradient, and time spent inactive were all significant predictors in the expected direction for weight, percent body fat, waist circumference, BMI, BRI, triglycerides, diastolic blood pressure, obesity, hypertension, and diabetes. The remaining physical activity metrics demonstrated similar trends overall (Fig. S2), with some metrics, especially self-affinity of acceleration signals (fractal complexity), generally having smaller effect sizes for many outcomes (i.e. waist circumference). Further, multiple regression models simultaneously estimating the contributions of inactivity and step counts or intensity gradient across biomarkers generally demonstrated independent, additive effects of these variables (Fig. S3); similar results were produced using alternative metrics of lifestyle in place of urbanicity (Fig. S4).

We also conducted exploratory analyses of our main physical activity variables including an age × physical activity interaction term, to investigate the extent to which physical activity impacts health differently across the life course. For a majority of our cardiometabolic outcome variables, elevated physical activity (or decreased time spent inactive) appears to be more strongly associated with health outcomes among younger individuals (Fig. S5, Table S6).

### Physical activity as a mediator of urbanicity effects on health

**Figure 5:**
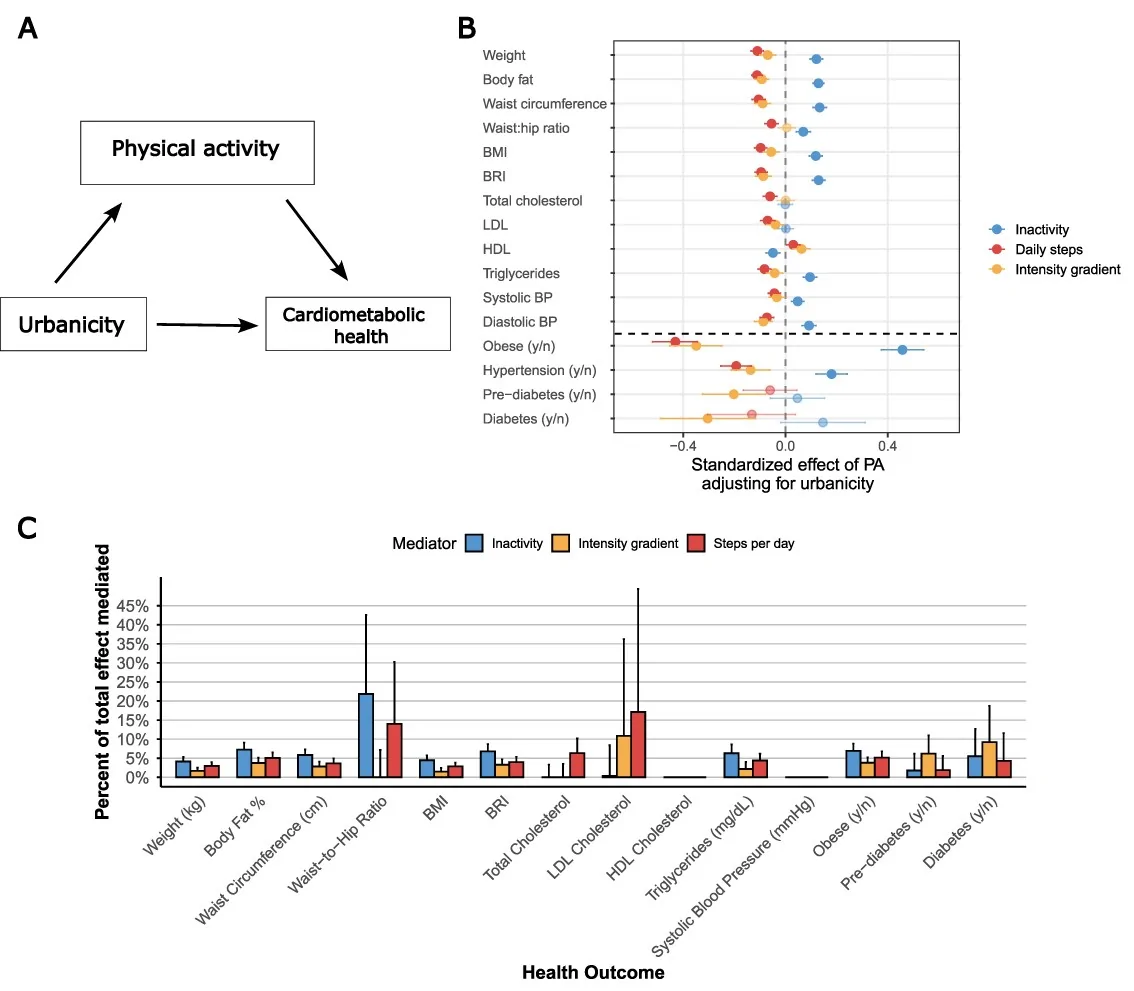
(A) Conceptual diagram for mediation analyses, showing physical activity as a potential mediator of the relationship between cardiometabolic health. (B) Forest plot of standardized coefficients ± 95% CIs in regressions adjusting for the effect of urbanicity. (C) The estimated percentage of the total urbanicity effect mediated by different physical activity metrics. Error bars represent upper 95% CIs. Estimates of negative mediation (suppression) were set to zero for plotting.

Finally, we conducted mediation analyses to quantify the extent to which the effects of urbanicity on cardiometabolic health operate through associated changes in physical activity (Fig. 5A). Models of health outcomes as a function of physical activity metrics adjusted for urbanicity revealed little difference relative to unadjusted models (Fig. 5B). Further, physical activity was a significant (posterior probability of direction >0.975) mediator of urbanicity effects on most observed cardiometabolic health outcomes (Fig. 5C), irrespective of the metric used to characterize urbanicity (Fig. S6). Daily step counts and intensity gradient were significant mediators for 10 out of 12 outcomes, and time spent inactive significantly mediated 8 out of 12 outcomes. Yet, the estimated proportion of the total effect mediated was generally modest or small: across all cardiometabolic outcomes and physical activity mediators, the median proportion mediated was 4.1% (range: 1.5%–21.6%) (Fig. 5C). Physical inactivity was the strongest mediator across many of the different health outcomes, though only by small margins and not for outcomes such as LDL cholesterol, total cholesterol, or pre-diabetes/diabetes.

## DISCUSSION

Abundant comparative evidence suggests that humans evolved in the context of very high levels of physical activity [11], especially compared to our closest primate relatives [17]. This evolved propensity is readily apparent among small-scale, non-industrial societies today who engage in high levels of physical activity, in addition to having excellent cardiometabolic health [1, 14, 18, 36]. Given that physical activity is a reliable predictor of improved health and mortality outcomes in industrialized contexts, it is often assumed that high levels of physical activity contribute substantially to these favorable cardiometabolic profiles [2, 4, 5, 37, 38]. Yet, rigorous, well-powered, and objective studies of physical activity in non-industrial societies are rare, and as a result the role of decreasing physical activity in driving deteriorating cardiometabolic health during lifestyle transitions to more urban, industrialized contexts remains poorly understood. Moreover, some recent findings call into question the extent to which high physical activity levels in subsistence contexts are a primary driver of low rates of overweight and obesity [12–14, 18].

Using a large dataset of objectively-measured physical activity and cardiometabolic health outcomes across an extreme lifestyle gradient, this study addresses three fundamental questions: (i) How does physical activity vary across the spectrum from non-industrial to more urban, industrial lifestyles?, (ii) Are higher physical activity levels associated with better biomarkers of cardiometabolic health?, and (iii) To what extent does physical activity mediate the relationship between more urban, industrial lifestyles and worse health?

### Physical activity and urbanization

We find clear evidence that objectively measured physical activity is reduced in more urban settings, regardless of the metric used to characterize activity (e.g. step counts, intensity gradient, time spent inactive) (Figs 2 and 3). Lower physical activity results from greater time spent inactive, and less time spent in all intensities (low, moderate, and vigorous) of activity (Fig. 3B). These findings closely mirror global trends reported for industrializing (i.e. developing) low- or middle-income nations like Malaysia, where urbanization has been consistently linked to decreases in physical activity [39–45].

Interestingly, among certain highly industrialized and post-industrial nations, evidence suggests that physical activity may sometimes be *lower* in more rural settings [46], mainly as a result of greater opportunities for recreational physical activity and/or less reliance on personal motorized vehicles in some urban cases [45]. Thus, it is possible that, on a global level, the relationship between physical activity and urbanization is somewhat *U*-shaped, with Orang Asli representing only the lower portion of the urbanization spectrum (i.e. without highly sedentary desk jobs), albeit the end of the spectrum represented by the bulk of the global population and that which is most relevant over human evolutionary history. Nevertheless, as observed in China and the United States [40, 47, 48], we expect that a large component of the shifts in Orang Asli physical activity are driven by occupation or work-related activities. Subsistence economies invariably require large amounts of low- and moderate-intensity activities such as walking long distances, food processing, and manual agricultural tasks like planting, weeding, and harvesting [17], whereas wage labor can directly result in either higher or lower levels of activity. Some common jobs for more market-integrated Orang Asli such as rubber tapping or working on palm oil plantations require repetitive and oftentimes hard physical labor, while other occupations (e.g. truck driving, retail work) entail large amounts of sedentary time.

One of our most striking results is that age-related patterns of physical activity vary widely across the lifestyle gradient. As observed almost universally around the world [49–52], physical activity decreases (and inactivity increases) with age among the Orang Asli (Fig. 1B). However, the slope of these relationships, especially after age 50–55 years when size-adjusted total energy expenditure declines in humans [53], are significantly steeper in individuals living more urbanized lifestyles (Fig. 3A). In fact, declines for Orang Asli in the most urban settings (~22% per decade from age 45–75 in step counts) were even greater than those reported in the largest epidemiological study to date, in which Doherty *et al.* [34] estimated a 7.5% decline per decade of acceleration vector magnitude for participants in the UK Biobank. Likewise, the modest age-associated activity decreases observed in older Orang Asli living the least industrialized lifestyles appeared similar to findings from other subsistence populations, including Hadza hunter-gatherers in Tanzania (females, but not males [18, 36]) and Tsimane forager-horticulturalists in Bolivia [14].

### Physical activity and cardiometabolic health

Higher levels of physical activity and less time spent inactive were significantly correlated with traits related to body composition and obesity, but also those related to blood lipids and blood pressure (Figs 4 and 5). These findings recapitulate a robust literature associating physical activity with cardiometabolic health, including randomized controlled trials and long-term prospective epidemiological studies [8, 9, 11, 54, 55], and are consistent with the idea that reductions in activity are playing a major role in the rapid rise of chronic cardiometabolic diseases globally [37]. Due to the cross-sectional and observational nature of our study, we are limited in our ability to infer causality or rule out the possibility that people with poor cardiometabolic health are less likely to be physically active due to loss of functional mobility [56], thus reversing the direction of causality. Nonetheless, repeated longitudinal studies [57, 58] and randomized controlled trials [55] generally support a causal pathway (Fig. 5A).

Given the low levels of vigorous-intensity physical activity observed across all Orang Asli in this study (Figs 1B and 3B), our findings support the inference that light- and moderate-intensity activities can have substantial cardiometabolic health benefits. This important finding echoes meta-analytical results from high-income industrialized populations demonstrating that all types of physical activity can be beneficial for longevity [8]. Notably, this result does not necessarily contradict other recent findings that light and moderate physical activity exhibit weaker dose–response curves to important health endpoints [20], but does raise questions about whether the relationship between physical activity type and health is context-dependent and could be fundamentally different in low- and middle-income nations compared to high-income countries. For example, whereas other studies report that occupational physical activity may not confer the same health benefits as leisure physical activity [59], most of the beneficial physical activity undertaken by Orang Asli in our study undoubtedly occurred in the context of work.

Some researchers have suggested that, given our long evolutionary history of likely having engaged in universally high levels of physical activity compared to other primates, active lifestyles may be necessary for protecting health and functional ability, especially as we age [11, 60]. Our data support this hypothesis and demonstrate that these associations persist even in quite rural and remote settings, where one might expect physical activity effects to have plateaued above generally high levels. Although this highlights critical health effects in humans, comparative (across species) tests remain necessary to determine whether evolution has more powerfully coupled physical activity to health due to our history as a “high-throughput” hunting and gathering species [17].

### Urbanization, physical activity, and cardiometabolic health

Our mediation analyses paint a surprising picture: physical activity is a significant mediator for many health outcomes, but appears to account for only a small proportion (median of 4.1%) of the total effect of urbanicity across health outcomes (Fig. 5C). This suggests that physical activity explains only a modest portion of the observed variance in cardiometabolic health that correlates with urbanicity. However, this is not because the effects of physical activity are merely subsumed by urbanicity. Although somewhat reduced in magnitude (Fig. S7), physical activity remains a robust, independent predictor of many cardiometabolic outcomes when controlling for urbanicity (Fig. 5B). We hypothesize that this result stems from the fact that the relationship between physical activity and health may be only weakly coupled with urbanization. For example, Orang Asli in highly urban environments of low socioeconomic status may be likely to work jobs requiring exhausting physical activity, whereas some individuals in very rural environments may nonetheless gain ready access to labor-saving devices, such as chainsaws, motorbikes, or water filtration systems. As a result, it is clear that public health interventions targeting sedentary behavior should not overlook remote communities in which reduced physical activity may appear well in advance of other more obvious environmental changes.

Our results underscore a large number of previous studies showing that variables other than physical activity, especially diet, play a critical role in driving cardiometabolic health changes in transitioning populations [e.g. 15, 61–64]. For example, a global study recently found only a weak association between total daily energy expenditure and obesity, and concluded that dietary intake plays a much greater role compared to energy expenditure in explaining higher rates of obesity in more economically developed contexts [64], potentially due to metabolic tradeoffs operating within constrained energy budgets [65]. Indeed, it is likely that a large proportion of the remaining urbanicity effect not explained in our study by physical activity is attributable to dietary factors. Although we did not include dietary covariates here due to the current availability of only relatively crude measures among Orang Asli [21], we have directly observed changes over the past ~10 years of the rapid replacement of traditional foods in many Orang Asli communities by store bought rice, ultra-processed packaged foods, and sweetened drinks (especially tea brewed with sugar and condensed milk), in addition to the frequent usage of additives including sugar, salt, and oil. These trends currently exist alongsidestubbornly high rates of food insecurity and undernourishment, leading to a dangerous “double-burden” of malnutrition [66]. Detailed studies of dietary intake in the Orang Asli are ongoing by OA HeLP, and more broadly, work in this area across other transitioning populations is a vital target of future research.

## CONCLUSIONS AND IMPLICATIONS

This study demonstrates that lifestyle change and urbanization are associated with decreases in objectively measured physical activity and concomitant worsening of cardiometabolic health. Despite physical activity being a common explanation for relationships between urbanization and health, physical activity appears to show largely independent effects and mediates only a moderate proportion of total urbanization effects. In addition, age-related physical activity declines in older adults, particularly above age 50, are notably more severe in more urban environments. Taken together, our findings support the general inference that recent global reductions in physical activity represent an evolutionarily novel challenge for our species, and that high levels of physical activity are an important contributor to the generally high quality of cardiometabolic health that has long been observed among subsistence populations around the world [1].

The proliferation of studies systematically collecting objective measures of physical activity offers tremendous promise for future research. In particular, there is much to be learned from studies of diverse populations experiencing disparate environmental conditions, especially as the expansion of globalization continues to generate “natural experiments” that can be leveraged to explore variation in the effects of changing physical activity profiles. In addition to large-scale synthesis of data and findings from small-scale subsistence societies to inform our understanding of what kinds of physical activity levels are representative for our species over evolutionary time, ripe areas of future study include longitudinal research in groups like the Orang Asli experiencing rapid lifestyle change, circadian analyses explicitly investigating the times of day when physical activity reductions are most acute in urban environments [33, 34], and investigation of the relative impacts of light/moderate/vigorous activities on health in subsistence contexts where recreational physical activity is low. Such research has only become more consequential following broad theoretical and empirical developments challenging traditional models of the relationship between physical activity and total daily energy expenditure [65], and will help clarify the particular ways in which modern lifestyles are contributing to the global rise of chronic non-communicable diseases.

## Supplementary Material

Supplementary_material_eoag017

## Data Availability

OA HeLP prioritizes minimizing risk to study participants and follows the CARE Principles for Indigenous Data Governance and the FAIR Guiding Principles for scientific data (emphasizing Findability, Accessibility, Interoperability and Reusability). Individual-level data are stored on Zenodo in a protected repository and available through restricted access at https://doi.org/10.5281/zenodo.20415057. Requests for de-identified data must include a detailed application and procedures for data security, privacy, and minimizing potential harm. Sample data use agreements are provided at orangaslihealth.org. Code to generate the urbanicity score is available on GitHub (https://github.com/mwatowich/Multi-population_lifestyle_scales). All code for analyses presented here are available in the Zenodo repository (https://doi.org/10.5281/zenodo.20415057).

## Notes

This article has been uploaded as a preprint at https://doi.org/10.1101/2025.07.11.25331394.
