# Supplementary material for: Physical activity and cardiometabolic health across an extreme lifestyle gradient
Source: Evol Med Public Health. 2026 Jul 21;14(1):1–15. doi: 10.1093/emph/eoag017 (PMC13489160; doi:10.1093/emph/eoag017)
Supplement: Supplementary file 1 [file supplementary_material_eoag017.zip › TabS6_bayes_2026_05_26.pdf]

Physical activity predictor: Inactivity

Predictor	Weight			Body fat			Waist circumference			Waist:hip ratio			BMI		
	Estimate	95% CrI	Pr(direction)	Estimate	95% CrI	Pr(direction)	Estimate	95% CrI	Pr(direction)	Estimate	95% CrI	Pr(direction)	Estimate	95% CrI	Pr(direction)
Intercept	0	[-0.04, 0.03]	0.561	0.51	[0.48, 0.54]	1	0.16	[0.12, 0.19]	1	-0.04	[-0.07, 0]	0.986	0.2	[0.17, 0.23]	1
Age (z)	-0.17	[-0.2, -0.13]	1	-0.01	[-0.04, 0.02]	0.792	0.09	[0.06, 0.13]	1	0.24	[0.21, 0.28]	1	-0.1	[-0.13, -0.07]	1
Age (z):Sex	0.05	[0, 0.11]	0.976	0.12	[0.08, 0.17]	1	0.04	[-0.01, 0.09]	0.932	-0.02	[-0.07, 0.04]	0.736	0.1	[0.05, 0.15]	1
Sex	0.13	[0.07, 0.19]	1	-1.2	[-1.24, -1.15]	1	-0.25	[-0.31, -0.19]	1	0.28	[0.22, 0.34]	1	-0.39	[-0.44, -0.33]	1
Smoking (y/n)	-0.15	[-0.21, -0.09]	1	-0.27	[-0.32, -0.22]	1	-0.23	[-0.29, -0.17]	1	-0.21	[-0.27, -0.16]	1	-0.2	[-0.26, -0.15]	1
Inactivity (z)	0.21	[0.18, 0.23]	1	0.18	[0.15, 0.2]	1	0.2	[0.17, 0.23]	1	0.08	[0.05, 0.11]	1	0.19	[0.17, 0.22]	1
Inactivity x age	-0.09	[-0.12, -0.07]	1	-0.04	[-0.06, -0.02]	1	-0.06	[-0.08, -0.04]	1	-0.02	[-0.04, 0.01]	0.926	-0.08	[-0.11, -0.06]	1

Predictor	BRI			Total cholesterol			LDL			HDL			Triglycerides			Systolic BP		
	Estimate	95% CrI	Pr(direction)	Estimate	95% CrI	Pr(direction)	Estimate	95% CrI	Pr(direction)	Estimate	95% CrI	Pr(direction)	Estimate	95% CrI	Pr(direction)	Estimate	95% CrI	Pr(direction)
Intercept	0.3	[0.27, 0.33]	1	0.15	[0.11, 0.18]	1	0.18	[0.14, 0.21]	1	0.21	[0.18, 0.25]	1	-0.19	[-0.22, -0.15]	1	-0.05	[-0.09, -0.02]	1
Age (z)	0.17	[0.14, 0.2]	1	0.25	[0.22, 0.29]	1	0.19	[0.16, 0.23]	1	0.17	[0.13, 0.2]	1	0.13	[0.1, 0.17]	1	0.53	[0.5, 0.56]	1
Age (z):Sex	0.01	[-0.04, 0.06]	0.705	-0.15	[-0.2, -0.1]	1	-0.09	[-0.14, -0.03]	0.999	0.07	[0.02, 0.12]	0.996	-0.1	[-0.16, -0.05]	1	-0.33	[-0.38, -0.28]	1
Sex	-0.62	[-0.67, -0.56]	1	-0.24	[-0.3, -0.18]	1	-0.29	[-0.36, -0.23]	1	-0.41	[-0.47, -0.35]	1	0.35	[0.29, 0.4]	1	0.53	[0.48, 0.59]	1
Smoking (y/n)	-0.26	[-0.31, -0.2]	1	-0.12	[-0.18, -0.06]	1	-0.18	[-0.24, -0.12]	1	-0.2	[-0.26, -0.14]	1	0.19	[0.13, 0.25]	1	-0.34	[-0.39, -0.28]	1
Inactivity (z)	0.18	[0.15, 0.21]	1	0.04	[0.01, 0.07]	0.995	0.01	[-0.02, 0.04]	0.829	-0.03	[-0.06, 0]	0.981	0.15	[0.12, 0.18]	1	0.03	[0, 0.05]	0.984
Inactivity x age	-0.03	[-0.06, -0.01]	0.997	-0.09	[-0.11, -0.06]	1	-0.02	[-0.05, 0]	0.951	-0.04	[-0.06, -0.01]	0.999	-0.1	[-0.13, -0.08]	1	0.05	[0.02, 0.07]	1

Predictor	Diastolic BP			Obese (y/n)			Hypertension (y/n)			Pre-diabetes (y/n)			Diabetes (y/n)		
	Estimate	95% CrI	Pr(direction)	Estimate	95% CrI	Pr(direction)	Estimate	95% CrI	Pr(direction)	Estimate	95% CrI	Pr(direction)	Estimate	95% CrI	Pr(direction)
Intercept	0.03	[0, 0.07]	0.972	-1.06	[-1.15, -0.97]	1	0.15	[0.07, 0.22]	1	-2.36	[-2.5, -2.23]	1	-3.51	[-3.73, -3.29]	1
Age (z)	0.3	[0.26, 0.33]	1	-0.19	[-0.28, -0.1]	1	0.69	[0.61, 0.77]	1	0.57	[0.44, 0.69]	1	0.64	[0.45, 0.83]	1
Age (z):Sex	-0.15	[-0.2, -0.1]	1	0.13	[-0.03, 0.3]	0.941	-0.28	[-0.41, -0.16]	1	-0.04	[-0.24, 0.16]	0.657	-0.32	[-0.62, -0.01]	0.978
Sex	0.16	[0.1, 0.22]	1	-1.05	[-1.24, -0.87]	1	0.51	[0.38, 0.64]	1	-0.25	[-0.51, -0.01]	0.977	-0.04	[-0.43, 0.34]	0.583
Smoking (y/n)	-0.25	[-0.31, -0.19]	1	-0.55	[-0.73, -0.37]	1	-0.65	[-0.78, -0.51]	1	-0.39	[-0.62, -0.16]	1	-0.35	[-0.71, 0]	0.975
Inactivity (z)	0.09	[0.06, 0.12]	1	0.56	[0.48, 0.64]	1	0.16	[0.1, 0.22]	1	0.15	[0.03, 0.27]	0.993	0.24	[0.05, 0.43]	0.993
Inactivity x age	0	[-0.02, 0.03]	0.596	-0.09	[-0.16, -0.02]	0.992	0.05	[-0.01, 0.11]	0.953	-0.09	[-0.18, 0]	0.975	-0.07	[-0.2, 0.07]	0.832

*Pr(direction) describes the proportion of the posterior that falls on the same side of 0 as the main association (i.e., a value of 0.96 can be interpreted as expressing 96% confidence that a given parameter is directionally greater or less than zero).

Physical activity predictor

Predictor	Weight			Body fat			Waist circumference			Waist:hip ratio			BMI		
	Estimate	95% CrI	Pr(direction)	Estimate	95% CrI	Pr(direction)	Estimate	95% CrI	Pr(direction)	Estimate	95% CrI	Pr(direction)	Estimate	95% CrI	Pr(direction)
Intercept	-0.01	[-0.05, 0.02]	0.73	0.5	[0.47, 0.53]	1	0.14	[0.11, 0.18]	1	-0.04	[-0.08, -0.01]	0.996	0.19	[0.16, 0.23]	1
Age (z)	-0.15	[-0.18, -0.11]	1	0	[-0.03, 0.02]	0.611	0.11	[0.07, 0.14]	1	0.25	[0.21, 0.28]	1	-0.08	[-0.12, -0.05]	1
Age (z):Sex	0.02	[-0.03, 0.08]	0.809	0.11	[0.06, 0.15]	1	0.02	[-0.03, 0.07]	0.766	-0.02	[-0.08, 0.03]	0.829	0.07	[0.02, 0.12]	0.998
Sex	0.2	[0.14, 0.26]	1	-1.13	[-1.18, -1.09]	1	-0.19	[-0.25, -0.13]	1	0.3	[0.25, 0.36]	1	-0.32	[-0.38, -0.26]	1
Smoking (y/n)	-0.16	[-0.21, -0.1]	1	-0.26	[-0.31, -0.22]	1	-0.23	[-0.29, -0.17]	1	-0.21	[-0.27, -0.15]	1	-0.2	[-0.26, -0.15]	1
Daily steps (z)	-0.18	[-0.21, -0.15]	1	-0.15	[-0.18, -0.13]	1	-0.16	[-0.19, -0.13]	1	-0.07	[-0.09, -0.04]	1	-0.16	[-0.19, -0.14]	1
Daily steps x age	0.11	[0.09, 0.14]	1	0.07	[0.05, 0.09]	1	0.08	[0.06, 0.11]	1	0.03	[0, 0.05]	0.986	0.11	[0.08, 0.13]	1

Predictor	BRI			Total cholesterol			LDL			HDL			Triglycerides			Systolic BP		
	Estimate	95% CrI	Pr(direction)	Estimate	95% CrI	Pr(direction)	Estimate	95% CrI	Pr(direction)	Estimate	95% CrI	Pr(direction)	Estimate	95% CrI	Pr(direction)	Estimate	95% CrI	Pr(direction)
Intercept	0.29	[0.26, 0.32]	1	0.14	[0.1, 0.17]	1	0.16	[0.13, 0.2]	1	0.22	[0.18, 0.25]	1	-0.19	[-0.23, -0.16]	1	-0.05	[-0.08, -0.02]	1
Age (z)	0.19	[0.15, 0.22]	1	0.26	[0.22, 0.29]	1	0.19	[0.15, 0.22]	1	0.16	[0.13, 0.2]	1	0.16	[0.12, 0.19]	1	0.52	[0.49, 0.55]	1
Age (z):Sex	0	[-0.05, 0.05]	0.556	-0.19	[-0.24, -0.14]	1	-0.11	[-0.16, -0.05]	1	0.05	[0, 0.1]	0.976	-0.14	[-0.2, -0.09]	1	-0.31	[-0.36, -0.27]	1
Sex	-0.55	[-0.61, -0.5]	1	-0.22	[-0.27, -0.16]	1	-0.27	[-0.33, -0.21]	1	-0.43	[-0.48, -0.37]	1	0.39	[0.33, 0.44]	1	0.54	[0.49, 0.59]	1
Smoking (y/n)	-0.26	[-0.31, -0.2]	1	-0.12	[-0.18, -0.06]	1	-0.17	[-0.23, -0.11]	1	-0.2	[-0.26, -0.14]	1	0.19	[0.13, 0.25]	1	-0.35	[-0.4, -0.29]	1
Daily steps (z)	-0.14	[-0.17, -0.11]	1	-0.09	[-0.12, -0.06]	1	-0.08	[-0.11, -0.05]	1	0.02	[-0.01, 0.05]	0.936	-0.13	[-0.16, -0.1]	1	-0.03	[-0.05, 0]	0.983
Daily steps x age	0.06	[0.04, 0.08]	1	0.09	[0.06, 0.11]	1	0.04	[0.01, 0.07]	0.998	0.01	[-0.02, 0.03]	0.754	0.11	[0.09, 0.13]	1	-0.05	[-0.07, -0.03]	1

Predictor	Diastolic BP			Obese (y/n)			Hypertension (y/n)			Pre-diabetes (y/n)			Diabetes (y/n)		
	Estimate	95% CrI	Pr(direction)	Estimate	95% CrI	Pr(direction)	Estimate	95% CrI	Pr(direction)	Estimate	95% CrI	Pr(direction)	Estimate	95% CrI	Pr(direction)
Intercept	0.03	[-0.01, 0.06]	0.927	-1.09	[-1.18, -1]	1	0.14	[0.06, 0.21]	1	-2.36	[-2.5, -2.23]	1	-3.55	[-3.78, -3.33]	1
Age (z)	0.29	[0.26, 0.33]	1	-0.16	[-0.25, -0.07]	1	0.68	[0.6, 0.77]	1	0.54	[0.42, 0.66]	1	0.6	[0.4, 0.79]	1
Age (z):Sex	-0.14	[-0.19, -0.09]	1	0.11	[-0.05, 0.27]	0.917	-0.28	[-0.4, -0.16]	1	-0.04	[-0.24, 0.15]	0.666	-0.33	[-0.62, -0.03]	0.985
Sex	0.19	[0.13, 0.25]	1	-0.85	[-1.03, -0.67]	1	0.59	[0.46, 0.72]	1	-0.23	[-0.49, 0.02]	0.965	0.03	[-0.37, 0.4]	0.556
Smoking (y/n)	-0.25	[-0.31, -0.2]	1	-0.56	[-0.74, -0.38]	1	-0.67	[-0.8, -0.53]	1	-0.37	[-0.6, -0.14]	1	-0.33	[-0.68, 0.01]	0.97
Daily steps (z)	-0.07	[-0.1, -0.04]	1	-0.53	[-0.62, -0.45]	1	-0.17	[-0.23, -0.11]	1	-0.14	[-0.26, -0.02]	0.99	-0.17	[-0.37, 0.03]	0.954
Daily steps x age	0	[-0.03, 0.02]	0.633	0.16	[0.09, 0.24]	1	-0.08	[-0.14, -0.02]	0.994	0.07	[-0.02, 0.17]	0.929	-0.01	[-0.15, 0.14]	0.528

*Pr(direction) describes the proportion of the posterior that falls on the same side of 0 as the main association (i.e., a value of 0.96 can be interpreted as expressing 96% confidence that a given parameter is directionally greater or less than zero).

Physical activity predictor: Intensity gradient

Predictor	Weight			Body fat			Waist circumference			Waist:hip ratio			BMI		
	Estimate	95% CrI	Pr(direction)	Estimate	95% CrI	Pr(direction)	Estimate	95% CrI	Pr(direction)	Estimate	95% CrI	Pr(direction)	Estimate	95% CrI	Pr(direction)
Intercept	-0.03	[-0.07, 0]	0.972	0.47	[0.44, 0.5]	1	0.12	[0.08, 0.16]	1	-0.04	[-0.08, -0.01]	0.991	0.17	[0.14, 0.21]	1
Age (z)	-0.15	[-0.19, -0.12]	1	-0.02	[-0.05, 0.01]	0.931	0.1	[0.06, 0.14]	1	0.26	[0.22, 0.29]	1	-0.08	[-0.12, -0.05]	1
Age (z):Sex	-0.04	[-0.09, 0.01]	0.934	0.07	[0.03, 0.12]	0.999	-0.04	[-0.09, 0.01]	0.929	-0.02	[-0.08, 0.03]	0.811	0.02	[-0.04, 0.07]	0.724
Sex	0.31	[0.24, 0.37]	1	-1.05	[-1.1, -1]	1	-0.07	[-0.13, -0.01]	0.985	0.3	[0.24, 0.36]	1	-0.23	[-0.29, -0.16]	1
Smoking (y/n)	-0.12	[-0.18, -0.06]	1	-0.24	[-0.29, -0.19]	1	-0.2	[-0.26, -0.14]	1	-0.2	[-0.26, -0.14]	1	-0.18	[-0.23, -0.12]	1
Intensity gradient (z)	-0.18	[-0.21, -0.15]	1	-0.15	[-0.18, -0.12]	1	-0.19	[-0.22, -0.15]	1	-0.01	[-0.05, 0.02]	0.757	-0.16	[-0.19, -0.12]	1
Intensity gradient x age	0.14	[0.12, 0.16]	1	0.06	[0.04, 0.08]	1	0.13	[0.1, 0.15]	1	0.03	[0, 0.05]	0.986	0.13	[0.1, 0.15]	1

Predictor	BRI			Total cholesterol			LDL			HDL			Triglycerides			Systolic BP		
	Estimate	95% CrI	Pr(direction)	Estimate	95% CrI	Pr(direction)	Estimate	95% CrI	Pr(direction)	Estimate	95% CrI	Pr(direction)	Estimate	95% CrI	Pr(direction)	Estimate	95% CrI	Pr(direction)
Intercept	0.27	[0.23, 0.3]	1	0.14	[0.1, 0.17]	1	0.16	[0.12, 0.2]	1	0.22	[0.19, 0.26]	1	-0.21	[-0.25, -0.18]	1	-0.06	[-0.09, -0.02]	1
Age (z)	0.18	[0.14, 0.21]	1	0.26	[0.23, 0.3]	1	0.19	[0.15, 0.23]	1	0.17	[0.13, 0.2]	1	0.14	[0.11, 0.18]	1	0.52	[0.49, 0.55]	1
Age (z):Sex	-0.04	[-0.1, 0.01]	0.954	-0.22	[-0.27, -0.16]	1	-0.13	[-0.19, -0.07]	1	0.07	[0.01, 0.12]	0.993	-0.18	[-0.23, -0.13]	1	-0.3	[-0.35, -0.25]	1
Sex	-0.45	[-0.51, -0.39]	1	-0.2	[-0.26, -0.13]	1	-0.23	[-0.29, -0.16]	1	-0.47	[-0.53, -0.41]	1	0.44	[0.37, 0.5]	1	0.55	[0.49, 0.6]	1
Smoking (y/n)	-0.23	[-0.28, -0.18]	1	-0.1	[-0.16, -0.04]	0.999	-0.16	[-0.22, -0.09]	1	-0.21	[-0.27, -0.15]	1	0.21	[0.15, 0.27]	1	-0.34	[-0.4, -0.29]	1
Intensity gradient (z)	-0.16	[-0.2, -0.13]	1	-0.04	[-0.08, -0.01]	0.993	-0.07	[-0.11, -0.04]	1	0.06	[0.02, 0.09]	0.999	-0.11	[-0.14, -0.07]	1	-0.02	[-0.05, 0.01]	0.863
Intensity gradient x age	0.1	[0.08, 0.12]	1	0.08	[0.05, 0.1]	1	0.07	[0.05, 0.1]	1	-0.02	[-0.04, 0]	0.968	0.08	[0.06, 0.11]	1	-0.03	[-0.05, -0.01]	0.991

Predictor	Diastolic BP			Obese (y/n)			Hypertension (y/n)			Pre-diabetes (y/n)			Diabetes (y/n)		
	Estimate	95% CrI	Pr(direction)	Estimate	95% CrI	Pr(direction)	Estimate	95% CrI	Pr(direction)	Estimate	95% CrI	Pr(direction)	Estimate	95% CrI	Pr(direction)
Intercept	0.02	[-0.02, 0.05]	0.852	-1.14	[-1.23, -1.05]	1	0.1	[0.02, 0.18]	0.995	-2.5	[-2.65, -2.36]	1	-3.7	[-3.95, -3.47]	1
Age (z)	0.29	[0.26, 0.33]	1	-0.15	[-0.25, -0.05]	0.999	0.67	[0.58, 0.75]	1	0.64	[0.51, 0.78]	1	0.63	[0.41, 0.85]	1
Age (z):Sex	-0.17	[-0.22, -0.12]	1	-0.02	[-0.2, 0.16]	0.571	-0.27	[-0.39, -0.14]	1	-0.2	[-0.42, 0.01]	0.972	-0.45	[-0.76, -0.14]	0.997
Sex	0.26	[0.2, 0.33]	1	-0.55	[-0.74, -0.36]	1	0.64	[0.5, 0.78]	1	0.12	[-0.17, 0.39]	0.792	0.37	[-0.04, 0.78]	0.959
Smoking (y/n)	-0.24	[-0.3, -0.18]	1	-0.48	[-0.65, -0.3]	1	-0.63	[-0.77, -0.5]	1	-0.35	[-0.58, -0.12]	0.999	-0.28	[-0.63, 0.07]	0.943
Intensity gradient (z)	-0.1	[-0.13, -0.07]	1	-0.54	[-0.64, -0.43]	1	-0.12	[-0.2, -0.05]	0.999	-0.6	[-0.76, -0.45]	1	-0.57	[-0.8, -0.33]	1
Intensity gradient x age	0.07	[0.04, 0.09]	1	0.37	[0.28, 0.46]	1	-0.02	[-0.08, 0.04]	0.737	0.4	[0.29, 0.51]	1	0.22	[0.07, 0.38]	0.997

*Pr(direction) describes the proportion of the posterior that falls on the same side of 0 as the main association (i.e., a value of 0.96 can be interpreted as expressing 96% confidence that a given parameter is directionally greater or less than zero).
