# Supplementary material for: Physical activity and cardiometabolic health across an extreme lifestyle gradient
Source: Evol Med Public Health. 2026 Jul 21;14(1):1–15. doi: 10.1093/emph/eoag017 (PMC13489160; doi:10.1093/emph/eoag017)
Supplement: Supplementary file 1 [file supplementary_material_eoag017.zip › config_anon.pdf]

Configuration file for GGIR

argument	value	context
do.report	c(2,3,4,5,6)	not applicable
f0	1	not applicable
f1	1173	not applicable
LC_TIME_backup	English_United States.1252	not applicable
mode	c(2,3,4,5,6)	not applicable
outputdir	E:/kraft/accelerometry/2025-05-15_output	not applicable
studyname	batch_run_2025-05-15	not applicable
GGIRread_version	1.0.5	not applicable
GGIRversion	3.2.6	not applicable
R_version	R version 4.4.3 (2025-02-28 ucrt)	not applicable
qwindow	c(0,24)	params_247
qlevels	c()	params_247
qwindow_dateformat	%d-%m-%Y	params_247
ilevels	c()	params_247
IVIS_windowsize_minutes	60	params_247
IVIS_epochsize_seconds	c()	params_247
IVIS.activity.metric	2	params_247
IVIS_acc_threshold	20	params_247
qM5L5	c()	params_247
MX.ig.min.dur	10	params_247
M5L5res	10	params_247
winhr	5	params_247
iglevels	c(0,25,50,75,100,125,150,175,200,225,250,275,300,325,350,375,400,425,450,475,500,525,550,575,600,625,650,675,700,725,750,775,800,825,850,875,900,925,950,975,1000,1025,1050,1075,1100,1125,1150,1175,1200,1225,1250,1275,1300,1325,1350,1375,1400,1425,1450,1475,1500,1525,1550,1575,1600,1625,1650,1675,1700,1725,1750,1775,1800,1825,1850,1875,1900,1925,1950,1975,2000,2025,2050,2075,2100,2125,2150,2175,2200,2225,2250,2275,2300,2325,2350,2375,2400,2425,2450,2475,2500,2525,2550,2575,2600,2625,2650,2675,2700,2725,2750,2775,2800,2825,2850,2875,2900,2925,2950,2975,3000,3025,3050,3075,3100,3125,3150,3175,3200,3225,3250,3275,3300,3325,3350,3375,3400,3425,3450,3475,3500,3525,	params_247
LUXthresholds	c(0,100,500,1000,3000,5000,10000)	params_247
LUX_cal_constant	c()	params_247
LUX_cal_exponent	c()	params_247
LUX_day_segments	c()	params_247
L5M5window	c(0,24)	params_247
cosinor	FALSE	params_247
part6CR	FALSE	params_247
part6HCA	FALSE	params_247
part6Window	c(start,end)	params_247

part6DFA	FALSE	params_247
clevels	c(30,150)	params_247
SRI2_WASOmin	30	params_247
includedaycrit	16	params_cleaning
ndayswindow	7	params_cleaning
strategy	1	params_cleaning
data_masking_strategy	1	params_cleaning
maxdur	9	params_cleaning
hrs.del.start	0	params_cleaning
hrs.del.end	0	params_cleaning
includedaycrit.part5	0.666666667	params_cleaning
excludefirstlast.part5	FALSE	params_cleaning
TimeSegments2ZeroFile	c()	params_cleaning
do.imp	TRUE	params_cleaning
data_cleaning_file	c()	params_cleaning
minimum_MM_length.part5	23	params_cleaning
excludefirstlast	FALSE	params_cleaning
includenightcrit	16	params_cleaning
excludefirst.part4	FALSE	params_cleaning
excludelast.part4	FALSE	params_cleaning
max_calendar_days	0	params_cleaning
nonWearEdgeCorrection	TRUE	params_cleaning
nonwear_approach	2023	params_cleaning
segmentWEARcrit.part5	0.5	params_cleaning
segmentDAYSPTcrit.part5	c(0.9,0)	params_cleaning
study_dates_file	E:/kraft/accelerometry/sdf_2025-05-15.csv	params_cleaning
study_dates_dateformat	%d-%m-%Y	params_cleaning
includecrit.part6	c(0.666666666666667,0.666666666666667)	params_cleaning
includenightcrit.part5	0	params_cleaning
nonwearFiltermaxHours	c()	params_cleaning
nonwearFilterWindow	c()	params_cleaning
overwrite	FALSE	params_general
acc.metric	ENMO	params_general
maxNcores	30	params_general
print.filename	FALSE	params_general
do.parallel	TRUE	params_general
windowsizes	c(5,900,3600)	params_general
desiredtz	Asia/Kuala_Lumpur	params_general
configtz	c()	params_general
idloc	1	params_general
dayborder	0	params_general
part5_agg2_60seconds	FALSE	params_general
sensor.location	wrist	params_general
expand_tail_max_hours	c()	params_general
recordingEndSleepHour	c()	params_general
dataFormat	raw	params_general
maxRecordingInterval	c()	params_general

extEpochData_timeformat	%d-%m-%Y %H:%M:%S	params_general
recording_split_times	c()	params_general
recording_split_timeformat	%d/%m/%Y %H:%M	params_general
recording_split_overlap	0	params_general
recording_split_ignore_edges	FALSE	params_general
do.anglex	FALSE	params_metrics
do.angley	FALSE	params_metrics
do.anglez	TRUE	params_metrics
do.zcx	FALSE	params_metrics
do.zcy	FALSE	params_metrics
do.zcz	FALSE	params_metrics
do.enmo	TRUE	params_metrics
do.lfenmo	FALSE	params_metrics
do.en	FALSE	params_metrics
do.mad	FALSE	params_metrics
do.enmoa	FALSE	params_metrics
do.roll_med_acc_x	FALSE	params_metrics
do.roll_med_acc_y	FALSE	params_metrics
do.roll_med_acc_z	FALSE	params_metrics
do.dev_roll_med_acc_x	FALSE	params_metrics
do.dev_roll_med_acc_y	FALSE	params_metrics
do.dev_roll_med_acc_z	FALSE	params_metrics
do.bfen	FALSE	params_metrics
do.hfen	FALSE	params_metrics
do.hfenplus	FALSE	params_metrics
do.lfen	FALSE	params_metrics
do.lfx	FALSE	params_metrics
do.lfy	FALSE	params_metrics
do.lfz	FALSE	params_metrics
do.hfx	FALSE	params_metrics
do.hfy	FALSE	params_metrics
do.hfz	FALSE	params_metrics
do.bfx	FALSE	params_metrics
do.bfy	FALSE	params_metrics
do.bfz	FALSE	params_metrics
do.brondcounts	FALSE	params_metrics
do.neishabouricounts	FALSE	params_metrics
hb	15	params_metrics
lb	0.2	params_metrics
n	4	params_metrics
zc.lb	0.25	params_metrics
zc.hb	3	params_metrics
zc.sb	0.01	params_metrics
zc.order	2	params_metrics
zc.scale	1	params_metrics
actilife_LFE	FALSE	params_metrics
epochvalues2csv	FALSE	params_output

save_ms5rawlevels	TRUE	params_output
save_ms5raw_format	RData	params_output
save_ms5raw_without_invalid	FALSE	params_output
storefolderstructure	FALSE	params_output
timewindow	c(WW,MM)	params_output
viewingwindow	1	params_output
dofirstpage	TRUE	params_output
visualreport	FALSE	params_output
week_weekend_aggregate.part5	FALSE	params_output
do.part3.pdf	FALSE	params_output
outliers.only	FALSE	params_output
criterror	3	params_output
do.visual	TRUE	params_output
do.sibreport	TRUE	params_output
do.part2.pdf	TRUE	params_output
sep_reports	,	params_output
sep_config	,	params_output
dec_reports	.	params_output
dec_config	.	params_output
visualreport_without_invalid	TRUE	params_output
old_visualreport	TRUE	params_output
visualreport_hrsPerRow	36	params_output
visualreport_focus	day	params_output
visualreport_validcrit	0	params_output
require_complete_lastnight_part5	FALSE	params_output
method_research_vars	c()	params_output
mvpathreshold	100	params_phyact
boutcriter	0.8	params_phyact
mvpadur	c(1,5,10)	params_phyact
boutcriter.in	0.9	params_phyact
boutcriter.lig	0.8	params_phyact
boutcriter.mvpa	0.8	params_phyact
threshold.lig	30	params_phyact
threshold.mod	100	params_phyact
threshold.vig	400	params_phyact
boutdur.mvpa	1	params_phyact
boutdur.in	c(1,10,30)	params_phyact
boutdur.lig	c(1,10)	params_phyact
frag.metrics	c()	params_phyact
part6_threshold_combi	30_100_400	params_phyact
chunksize	1	params_rawdata
spherecrit	0.3	params_rawdata
minloadcrit	168	params_rawdata
printsummary	FALSE	params_rawdata
do.cal	TRUE	params_rawdata
backup.cal.coef	retrieve	params_rawdata
dynrange	c()	params_rawdata

minimumFileSizeMB	2	params_rawdata
rmc.dec	.	params_rawdata
rmc.firstrow.acc	c()	params_rawdata
rmc.firstrow.header	c()	params_rawdata
rmc.header.length	c()	params_rawdata
rmc.col.acc	c(1,2,3)	params_rawdata
rmc.col.temp	c()	params_rawdata
rmc.col.time	c()	params_rawdata
rmc.unit.acc	g	params_rawdata
rmc.unit.temp	C	params_rawdata
rmc.unit.time	POSIX	params_rawdata
rmc.format.time	%Y-%m-%d %H:%M:%OS	params_rawdata
rmc.bitrate	c()	params_rawdata
rmc.dynamic_range	c()	params_rawdata
rmc.unsignedbit	TRUE	params_rawdata
rmc.origin	1970-01-01	params_rawdata
rmc.desiredtz	c()	params_rawdata
rmc.configtz	c()	params_rawdata
rmc.sf	c()	params_rawdata
rmc.headername.sf	c()	params_rawdata
rmc.headername.sn	c()	params_rawdata
rmc.headername.recordingid	c()	params_rawdata
rmc.header.structure	c()	params_rawdata
rmc.check4timegaps	FALSE	params_rawdata
rmc.noise	13	params_rawdata
nonwear_range_threshold	150	params_rawdata
rmc.col.wear	c()	params_rawdata
rmc.doresample	FALSE	params_rawdata
interpolationType	1	params_rawdata
imputeTimegaps	TRUE	params_rawdata
frequency_tol	0.1	params_rawdata
rmc.scalefactor.acc	1	params_rawdata
anglethreshold	5	params_sleep
timethreshold	5	params_sleep
ignorenonwear	TRUE	params_sleep
HASPT.algo	HDCZA	params_sleep
HASIB.algo	vanHees2015	params_sleep
Sadeh_axis		params_sleep
longitudinal_axis	c()	params_sleep
HASPT.ignore.invalid	FALSE	params_sleep
loglocation	c()	params_sleep
colid	1	params_sleep
coln1	2	params_sleep
nnights	c()	params_sleep
relyonguider	FALSE	params_sleep
def.noc.sleep	1	params_sleep
sleeplogsep	c()	params_sleep

sleepwindowType	SPT	params_sleep
possible_nap_window	c()	params_sleep
possible_nap_dur	c()	params_sleep
possible_nap_gap	0	params_sleep
possible_nap_edge_acc	Inf	params_sleep
nap_model	c()	params_sleep
sleepefficiency.metric	1	params_sleep
HDCZA_threshold	c()	params_sleep
oakley_threshold	20	params_sleep
consider_marker_button	FALSE	params_sleep
impute_marker_button	FALSE	params_sleep
sib_must_fully_overlap_with_Tim	c(TRUE,TRUE)	params_sleep
nap_markerbutton_method	0	params_sleep
