# Supplementary material for: Physical activity and cardiometabolic health across an extreme lifestyle gradient
Source: Evol Med Public Health. 2026 Jul 21;14(1):1–15. doi: 10.1093/emph/eoag017 (PMC13489160; doi:10.1093/emph/eoag017)
Supplement: Supplementary file 1 [file supplementary_material_eoag017.zip › PA_and_health_final_supplement.docx]

Supplementary Information for Kraft et al. “Physical activity and cardiometabolic health across an extreme lifestyle gradient”

**Table of contents:**

- Supplementary Materials and Methods
- Supplementary Text
- Figure S1. Correlation plot of the cardiometabolic health outcomes.
- Figure S2. Main analysis results including all metrics of physical activity
- Figure S3. The effects of daily step counts and intensity gradient on cardiometabolic health outcomes controlling for inactivity
- Figure S4. The effects of alternative urbanicity metrics on physical activity outcomes
- Figure S5. The effects of a physical activity x age interaction on health.
- Figure S6. The effects of physical activity on health, adjusted for alternative urbanicity metrics
- Figure S7. Adjusting for urbanicity minimally changes the relationship between physical activity and cardiometabolic health
- Table S1. Descriptive statistics of the study sample
- Table S2. Information on urbanicity score calculations
- Table S3. Full model results for analyses describing urbanicity effects on health (provided as a pdf)
- Table S4. Full model results for analyses describing physical activity effects on health (provided as a pdf)
- Table S5. Full model results for analyses describing physical activity effects on health, controlling for urbanicity (provided as a pdf)
- Table S6. Full model results for analyses describing the physical activity x age interaction effects on health (provided as a pdf).
- Supplementary references

**Supplementary Materials and Methods**

*Study Population*

The Orang Asli, or “original people,” comprise 19 distinct ethnolinguistic groups that collectively represent the Indigenous peoples of Peninsular Malaysia. Data for this study come from 34 villages that are predominantly occupied by the following groups: Batek, Batek Nogn, Jahai, Jahut, Jakun, Kensiu, Semai, Semaq Beri, Temiar, and Temuan. These groups have long been separated by anthropologists into three larger groups—Senoi, Proto Malay, and Negrito—based primarily on language and subsistence type, and phenotypic differences [1,2].

The Batek, Batek Nogn, Jahai, and Kensiu are generally categorized as “Negrito” or “Semang” (both terminologies with controversial colonial histories), speak languages in the Northern Aslian sub-branch of the Austroasiatic language family, and traditionally lived as nomadic hunter-gatherers and traders of forest products primarily in the north and central regions of Peninsular Malaysia [3]. The Semai, Semaq Beri, and Temiar are collectively referred to as “Senoi”, speak languages in the Central Aslian subbranch of the Austroasiatic language family, live mainly in the central regions of Peninsular Malaysia including highland areas of the main range, and traditionally lived sedentary or semi-nomadic lifestyles practicing swidden horticulture (mixed crops with staples primarily consisting of millet, cassava, sweet potato, hill rice, taro, and banana) supplemented with fishing and hunting. In contrast, the Jakun and Temuan live primarily in the south and are categorized as “Proto-Malay”, speak Austronesian languages, and practice mixed subsistence strategies including permanent agriculture, riverine or coastal fishing, and wage labor or professional jobs. The Proto-Malay are generally more market integrated compared to other Orang Asli. In addition, all of these Orang Asli groups have rich and varied histories that have resulted in distinct religions, coresidence patterns, marriage practices, mobility patterns, and other important aspects of socioecology (for more information on these groups, we refer readers to: [1–9]).

A notable feature of the Orang Asli environment is the dramatic lifestyle gradient that exists today, ranging from semi-nomadic practitioners of foraging lifestyles to fully sedentarized groups living in urban or peri-urban centers who are fully engaged with the national market economy [10]. Among the most urban groups, Orang Asli tend to be of relatively low socioeconomic status, with low levels of education compared to majority ethnic groups and low rates of higher paying, sedentary office jobs. Although most Orang Asli today have some engagement with the market economy, the nature and extent of this engagement is highly variable. For example, the economies of some Temuan communities in close proximity to Kuala Lumpur are highly dependent on wage labor, with community members working a variety of wage labor jobs (i.e., shopkeeper, hotel worker, resort staff, plantation worker), whereas some Batek and Jahai communities have only a minority of or no individuals working wage labor jobs (mostly in sectors such as tourism or rubber-tapping), with others instead relying on a mix of subsistence foraging, small-scale cropping, and the collection and trade of forest products.

*Accelerometry Data Processing*

Raw accelerometry files were downloaded and pre-processed to remove any days recorded before or after participants wore the devices. Next, we used the *GGIR* package (v3.2.6) [11,12] to process the data into 5-second epochs for analysis using default auto-calibration and imputation algorithms. Non-wear time was also estimated using built-in *GGIR* algorithms, and we defined a valid day for inclusion as those in which ≥ 16 hours of wear time were detected. A *GGIR* configuration file describing the full set of parameters used in our analysis is provided as supplementary material. Physical activity metrics were calculated for each person-day (with days calculated from midnight - midnight), but for some analyses averages were taken at the individual level.

*Measures of cardiometabolic health*

Sixteen cardiometabolic phenotypes were measured or derived for participants. These include body weight (kg), body fat percentage (from bioimpedance using Tanita BC-558), waist circumference (cm), body mass index (BMI), body roundness index (BRI) [13], waist-to-hip ratio, total cholesterol, high-density and low-density lipoprotein (HDL, LDL) cholesterol, triglycerides (mg/dL), and systolic and diastolic blood pressure (mmHg; Omron 5 Series Upper Arm Blood Pressure Monitor). We also characterized whether the individual fit criteria for being hypertensive (systolic blood pressure ≥ 130 or diastolic blood pressure ≥ 80), obese (BMI ≥ 30), prediabetic (6.5 > HbA1c ≥ 5.7 or 125 > non-fasting glucose ≥ 199 in few cases where HbA1c not available), or diabetic (HbA1c ≥ 6.5 or non-fasting glucose ≥ 200 in few cases where HbA1c not available). Sample characteristics are described in Table S1. Prior to standardization, several variables with highly skewed distributions were transformed to approximate normality: square-root transformations were applied to total cholesterol, LDL, HDL, and triglycerides; systolic blood pressure was natural log transformed. Variables with binary outcomes (e.g., hypertension) were not transformed or standardized.

**Supplementary Text**

*Physical activity and cardiometabolic health*

Stronger and more consistent associations between physical activity and body composition, versus blood pressure or lipids, are potentially a reflection of the relative roles of physical activity vis a vis other factors such as diet in determining these traits. This finding was unexpected, but not unprecedented. For example, Leon and Sanchez [14] aggregated published studies to demonstrate markedly inconsistent results between exercise/endurance training and blood lipids, whereas “all lipid parameters generally improved in studies in which there was a substantial weight loss (≥ 4 kg), usually associated with a concomitant hypocaloric diet.” Similarly, strong evidence links blood pressure to dietary factors [15], and in some cases with stronger effects than physical activity [16], though both are likely necessary components of hypertension control and prevention [17]. Regardless, these findings raise the possibility that Orang Asli living more urbanized lifestyles may, at least in some cases, exhibit higher rates of obesity without experiencing a commensurate increase in other facets of metabolic syndrome, such as hypertension and hyperlipidemia.

**Figure S1.** Correlations among the cardiometabolic traits in this study. Colors correspond to Pearson correlation coefficients.

**Figure S2. Main analysis results including all metrics of physical activity.** (A) The standardized effect of urbanicity on different physical activity metrics, adjusting for age by sex interactions with a random intercept to account for repeat observations of individuals. Bars represent 95% credible intervals. (B) Forest plot of standardized effects of all physical activity variables measured in this study on the full suite of cardiometabolic health outcomes. All models adjust for age by sex interactions and smoking (as in Fig. 4 in the main text).

**Figure S3. The effects of (A) daily step counts and (B) intensity gradient on cardiometabolic health outcomes controlling for inactivity**. Forest plots represent model estimated effects (±95% credible intervals) from multiple regression models that included the focal physical activity predictor as well as time spent inactive. All models are weighted by the number of valid measurement days of physical activity and adjusted for smoking and an age x sex interaction.

**Figure S4.** Predicted levels of physical activity (standardized) as a function of alternative metrics of urbanicity and lifestyle: (A) first factor from a factor analysis of lifestyle variables across all domains, (B): “Household style of life.” See Watowich et al. (2026) for more details on these metrics. Results in this figure reflect the same models shown in Fig. 2C, but with different variables substituted for urbanicity.

**Figure S5.** Forest plot of standardized coefficients ± 95% credible intervals for terms representing the interaction between age and physical activity (time spent inactive, daily step counts, and intensity gradient) in exploratory regressions of cardiometabolic health outcomes. As an example to aid in interpretation, a positive interaction coefficient for waist circumference indicates that there is a stronger negative association between step counts or intensity gradient and waist circumference in younger individuals (i.e., physical activity variables have increased effects on health at younger ages).

**Figure S6.** Forest plot of standardized coefficients ± 95% credible intervals in regressions of cardiometabolic health outcomes as a function of physical activity, adjusting for the effect of alternative metrics of urbanicity and lifestyle: : (A) first factor from a factor analysis of lifestyle variables across all domains, (B): “Household style of life.”

**Figure S7. Adjusting for urbanicity minimally changes the relationship between physical activity and cardiometabolic health.** The x-axis represents model coefficients for effects of physical activity (PA) variables on health outcomes in models adjusting for urbanicity score, whereas the y-axis represents coefficients from the same models but without adjusting for urbanicity score.

**Table S1: Descriptive statistics for the study sample, by sex. All reported values are means of untransformed variables.**

| **Variable** | **Female** | **Male** |
| --- | --- | --- |
| Number of unique individuals | 684 | 391 |
| Age (years) | 36.9 | 42.5 |
| Inactivity time (min) | 602.5 | 638.2 |
| Light activity time (min) | 302.7 | 274.7 |
| Moderate activity time (min) | 122.8 | 125.8 |
| Vigorous activity time (min) | 4.1 | 8.7 |
| ENMO | 32.5 | 34.2 |
| Steps/day | 11325.0 | 11759.5 |
| Intensity gradient | -2.6 | -2.5 |
| DFA | 1.0 | 1.0 |
| Weight (kg) | 58.2 | 59.2 |
| Body fat (% | 29.5 | 15.6 |
| Waist circumference (cm) | 83.7 | 80.5 |
| Waist:hip ratio | 0.9 | 0.9 |
| BMI | 25.9 | 23.3 |
| BRI | 4.7 | 3.6 |
| Total cholesterol (mg/dL) | 180.8 | 169.2 |
| LDL (mg/dL) | 110.5 | 98.6 |
| HDL (mg/dL) | 49.3 | 44.4 |
| Triglycerides | 119.7 | 148.4 |
| Systolic blood pressure (mm Hg) | 117.1 | 127.7 |
| Diastolic blood pressure (mm Hg) | 77.8 | 80.0 |
| Obese (%) | 24.3 | 9.6 |
| Hypertensive (%) | 46.8 | 60.3 |
| Pre-diabetic (%) | 8.3 | 7.7 |
| Diabetic (%) | 3.0 | 3.3 |

**Table S2. Information on main urbanicity score calculations.**

These items were tallied to create an urbanicity score for each location:

Scaled population density

10 - (10*Proportion listed as not foraging, agriculture, fishing)

5*Proportion of households with flush toilets

5*Proportion of households with electricity

5*Proportion of households with television

5*Proportion of households with smart phones

10*Proportion of >40yr olds with some education

10*Proportion of <40yr olds with some education.

Population density was scaled as follows:

| Score | Density per square kilometer |
| --- | --- |
| 0.67 | 0-99 |
| 1.33 | 100-199 |
| 2.00 | 200-299 |
| 2.67 | 300-399 |
| 3.33 | 400-499 |
| 4.00 | 500-999 |
| 4.67 | 1000-1999 |
| 5.33 | 2000-2999 |
| 6.00 | 3000-3999 |
| 6.67 | 4000-5999 |
| 7.33 | 6000-7999 |
| 8.00 | 8000-9999 |
| 8.67 | 10000-14999 |
| 9.33 | 15000-19999 |
| 10.00 | 20000-Inf |

**Table S3:** Full model results for analyses describing urbanicity effects on health.

See additional pdf file.

**Table S4:** Full model results for analyses describing physical activity effects on health.

See additional pdf file.

**Table S5:** Full model results for analyses describing physical activity effects on health, controlling for urbanicity.

See additional pdf file.

**Table S6:** Full model results for analyses describing the physical activity x age interaction effects on health.

See additional pdf file.

**Supplementary References**

1.	Endicott KM. Introduction. In: Endicott KM (ed.), *Malaysia’s Original People: Past, Present and Future of the Orang Asli*. Singapore: NUS Press, 2016, 1–38.

2.	Carey I. *Orang Asli: The Aboriginal Tribes of Peninsular Malaysia*. Kuala Lumpur: Oxford University Press, 1976.

3.	Blust R. Terror from the sky: Unconventional linguistic clues to the Negrito past. *Human Biology* 2013;**85**(1):401–16.

4.	Lye TP. *Changing Pathways: Forest Degradation and the Batek of Pahang, Malaysia*. Lanham: Lexington Books, 2004.

5.	Endicott KM, Endicott KL. *The Headman Was a Woman: The Gender Egalitarian Batek of Malaysia*. Long Grove, IL: Waveland Press, 2008.

6.	Benjamin G. *Temiar Religion, 1964-2012: Enchantment, Disenchantment and Re-Enchantment in Malaysia’s Uplands*. Singapore: NUS Press, 2014.

7.	Dentan RK. *The Semai: A Nonviolent People of Malaya*. New York: Holt, Rinehart and Winston, 1968.

8.	Nicholas C. Organizing Orang Asli Identity. In: Benjamin G, Chou C (eds), *Tribal Communities in the Malay World: Historical, Cultural and Social Perspectives*. Singapore: ISEAS Publishing, 2002, 119–36.

9.	Burenhult N. A Grammar of Jahai. PhD Thesis, Lund University, 2002.

10.	Wallace IJ, Lea AJ, Lim YA *et al.* Orang Asli Health and Lifeways Project (OA HeLP): a cross-sectional cohort study protocol. *BMJ Open* 2022;**12**(9):e058660.

11.	Hees VT van, Fang Z, Zhao JH *et al.* *Package ‘GGIR’*. 2025.

12.	Migueles JH, Rowlands AV, Huber F *et al.* GGIR: a research community–driven open source R package for generating physical activity and sleep outcomes from multi-day raw accelerometer data. *Journal for the Measurement of Physical Behaviour* 2019;**2**(3):188–96.

13.	Thomas DM, Bredlau C, Bosy-Westphal A *et al.* Relationships between body roundness with body fat and visceral adipose tissue emerging from a new geometrical model. *Obesity* 2013;**21**(11):2264–71.

14.	Leon, AS, Sanchez, Otto A. Response of blood lipids to exercise training alone or combined with dietary intervention. *Medicine & Science in Sports & Exercise* 2001;**33**(6):S502–15.

15.	Gay HC, Rao SG, Vaccarino V *et al.* Effects of different dietary interventions on blood pressure. *Hypertension* 2016;**67**(4):733–9. https://doi.org/10.1161/HYPERTENSIONAHA.115.06853.

16.	Camões M, Oliveira A, Pereira M *et al.* Role of physical activity and diet in incidence of hypertension: a population-based study in Portuguese adults. *Eur J Clin Nutr* 2010;**64**(12):1441–9. https://doi.org/10.1038/ejcn.2010.170.

17.	Zhu Y, Wang Z. Association between joint physical activity and healthy dietary patterns and hypertension in US adults: cross-sectional NHANES study. *BMC Public Health* 2024;**24**:855. https://doi.org/10.1186/s12889-024-18346-8.
