# Supplementary material for: Physical activity and cardiometabolic health across an extreme lifestyle gradient
Source: Evol Med Public Health. 2026 Jul 21;14(1):1–15. doi: 10.1093/emph/eoag017 (PMC13489160; doi:10.1093/emph/eoag017)
Supplement: Supplementary file 1 [file supplementary_material_eoag017.zip › TabS3_bayes_2026_04_14.pdf]

Bayesian models of urbanicity effects on health

	Weight			Body fat			Waist circumference			Waist:hip ratio		
Predictor	Estimate	95% CrI	Pr(direction)	Estimate	95% CrI	Pr(direction)	Estimate	95% CrI	Pr(direction)	Estimate	95% CrI	Pr(direction)
Intercept	**-0.09**	**[-0.16, -0.02]**	**0.997**	**0.4**	**[0.35, 0.46]**	**1**	0.05	[-0.02, 0.12]	0.922	**-0.09**	**[-0.16, -0.02]**	**0.99**
Age (z)	**-0.22**	**[-0.29, -0.15]**	**1**	-0.06	[-0.12, 0.01]	0.963	0.03	[-0.04, 0.1]	0.791	**0.22**	**[0.15, 0.3]**	**1**
Age (z):Sex	0.02	[-0.09, 0.13]	0.653	**0.12**	**[0.02, 0.21]**	**0.993**	0.06	[-0.06, 0.17]	0.827	0.02	[-0.1, 0.14]	0.609
Sex	**0.24**	**[0.13, 0.35]**	**1**	**-1.16**	**[-1.26, -1.07]**	**1**	**-0.19**	**[-0.31, -0.07]**	**0.999**	**0.25**	**[0.13, 0.38]**	**1**
Urbanicity score (z)	**0.41**	**[0.35, 0.46]**	**1**	**0.27**	**[0.22, 0.31]**	**1**	**0.33**	**[0.27, 0.39]**	**1**	**0.06**	**[0, 0.12]**	**0.98**

	BMI			BRI			Total cholesterol			LDL		
Predictor	Estimate	95% CrI	Pr(direction)	Estimate	95% CrI	Pr(direction)	Estimate	95% CrI	Pr(direction)	Estimate	95% CrI	Pr(direction)
Intercept	**0.1**	**[0.04, 0.17]**	**0.999**	**0.2**	**[0.13, 0.27]**	**1**	**0.1**	**[0.02, 0.17]**	**0.996**	**0.12**	**[0.05, 0.2]**	**0.999**
Age (z)	**-0.14**	**[-0.21, -0.07]**	**1**	**0.12**	**[0.05, 0.2]**	**1**	**0.23**	**[0.15, 0.31]**	**1**	**0.18**	**[0.1, 0.26]**	**1**
Age (z):Sex	0.06	[-0.05, 0.18]	0.872	0.03	[-0.08, 0.14]	0.706	**-0.15**	**[-0.27, -0.03]**	**0.992**	-0.07	[-0.19, 0.06]	0.857
Sex	**-0.31**	**[-0.42, -0.19]**	**1**	**-0.59**	**[-0.7, -0.47]**	**1**	**-0.25**	**[-0.37, -0.13]**	**1**	**-0.34**	**[-0.47, -0.21]**	**1**
Urbanicity score (z)	**0.37**	**[0.31, 0.42]**	**1**	**0.27**	**[0.22, 0.33]**	**1**	**0.13**	**[0.07, 0.19]**	**1**	**0.06**	**[0, 0.13]**	**0.981**

	HDL			Triglycerides			Systolic BP			Diastolic BP		
Predictor	Estimate	95% CrI	Pr(direction)	Estimate	95% CrI	Pr(direction)	Estimate	95% CrI	Pr(direction)	Estimate	95% CrI	Pr(direction)
Intercept	**0.16**	**[0.09, 0.23]**	**1**	**-0.17**	**[-0.24, -0.1]**	**1**	**-0.11**	**[-0.18, -0.04]**	**1**	-0.02	[-0.1, 0.05]	0.743
Age (z)	**0.1**	**[0.02, 0.18]**	**0.995**	**0.15**	**[0.08, 0.23]**	**1**	**0.51**	**[0.44, 0.58]**	**1**	**0.28**	**[0.2, 0.35]**	**1**
Age (z):Sex	0.1	[-0.02, 0.22]	0.943	**-0.18**	**[-0.3, -0.06]**	**0.999**	**-0.24**	**[-0.35, -0.13]**	**1**	-0.07	[-0.19, 0.05]	0.87
Sex	**-0.48**	**[-0.6, -0.35]**	**1**	**0.5**	**[0.38, 0.62]**	**1**	**0.39**	**[0.28, 0.5]**	**1**	0.08	[-0.04, 0.21]	0.908
Urbanicity score (z)	**0.07**	**[0.01, 0.13]**	**0.993**	**0.21**	**[0.15, 0.27]**	**1**	**-0.06**	**[-0.11, -0.01]**	**0.988**	0	[-0.06, 0.06]	0.545

	Obese (y/n)			Hypertension (y/n)			Pre-diabetes (y/n)			Diabetes (y/n)		
Predictor	Estimate	95% CrI	Pr(direction)	Estimate	95% CrI	Pr(direction)	Estimate	95% CrI	Pr(direction)	Estimate	95% CrI	Pr(direction)
Intercept	**-1.38**	**[-1.58, -1.19]**	**1**	-0.01	[-0.17, 0.15]	0.549	**-2.48**	**[-2.77, -2.2]**	**1**	**-3.64**	**[-4.14, -3.2]**	**1**
Age (z)	**-0.32**	**[-0.52, -0.12]**	**0.999**	**0.67**	**[0.49, 0.85]**	**1**	**0.48**	**[0.22, 0.74]**	**1**	**0.49**	**[0.11, 0.88]**	**0.993**
Age (z):Sex	0.15	[-0.24, 0.54]	0.772	-0.18	[-0.46, 0.1]	0.897	0.02	[-0.41, 0.44]	0.538	-0.31	[-0.93, 0.32]	0.84
Sex	**-0.93**	**[-1.32, -0.55]**	**1**	**0.36**	**[0.1, 0.63]**	**0.997**	-0.3	[-0.84, 0.22]	0.874	0.11	[-0.63, 0.81]	0.623
Urbanicity score (z)	**0.84**	**[0.68, 1.01]**	**1**	0	[-0.13, 0.12]	0.519	**0.31**	**[0.08, 0.54]**	**0.995**	**0.4**	**[0.05, 0.76]**	**0.989**

*Pr(direction) describes the proportion of the posterior that falls on the same side of 0 as the main association (i.e., a value of 0.96 can be interpreted as expressing 96% confidence that a given parameter is directionally greater or less than zero).
