# Supplementary material for: Physical activity and cardiometabolic health across an extreme lifestyle gradient
Source: Evol Med Public Health. 2026 Jul 21;14(1):1–15. doi: 10.1093/emph/eoag017 (PMC13489160; doi:10.1093/emph/eoag017)
Supplement: Supplementary file 1 [file supplementary_material_eoag017.zip › TabS4_bayes_2026_05_21.pdf]

Physical activity predictor: Inactivity

	Weight			Body fat			Waist circumference			Waist:hip ratio			BMI			BRI			Total cholesterol			LDL		
Predictor	Estimate	95% CrI	Pr(direction)	Estimate	95% CrI	Pr(direction)	Estimate	95% CrI	Pr(direction)	Estimate	95% CrI	Pr(direction)	Estimate	95% CrI	Pr(direction)	Estimate	95% CrI	Pr(direction)	Estimate	95% CrI	Pr(direction)	Estimate	95% CrI	Pr(direction)
Intercept	-0.02	[-0.05, 0.02]	0.807	0.51	[0.48, 0.54]	1	0.15	[0.11, 0.18]	1	-0.04	[-0.08, -0.01]	0.99	0.19	[0.15, 0.22]	1	0.3	[0.26, 0.33]	1	0.14	[0.1, 0.17]	1	0.18	[0.14, 0.21]	1
Age (z)	-0.16	[-0.19, -0.13]	1	-0.01	[-0.04, 0.02]	0.714	0.09	[0.06, 0.13]	1	0.24	[0.21, 0.28]	1	-0.1	[-0.13, -0.06]	1	0.17	[0.14, 0.21]	1	0.25	[0.22, 0.29]	1	0.19	[0.16, 0.23]	1
Age (z):Sex	0.01	[-0.04, 0.06]	0.661	0.11	[0.07, 0.15]	1	0.02	[-0.04, 0.07]	0.737	-0.02	[-0.07, 0.03]	0.805	0.06	[0.01, 0.12]	0.994	0	[-0.05, 0.05]	0.548	-0.18	[-0.24, -0.13]	1	-0.09	[-0.15, -0.04]	1
Sex	0.12	[0.07, 0.18]	1	-1.2	[-1.25, -1.15]	1	-0.25	[-0.31, -0.2]	1	0.28	[0.22, 0.33]	1	-0.39	[-0.45, -0.33]	1	-0.61	[-0.67, -0.56]	1	-0.25	[-0.31, -0.19]	1	-0.29	[-0.35, -0.23]	1
Smoking (y/n)	-0.15	[-0.21, -0.1]	1	-0.27	[-0.32, -0.22]	1	-0.23	[-0.29, -0.17]	1	-0.21	[-0.27, -0.15]	1	-0.2	[-0.26, -0.15]	1	-0.26	[-0.32, -0.2]	1	-0.12	[-0.18, -0.06]	1	-0.18	[-0.24, -0.12]	1
Inactivity (z)	0.18	[0.16, 0.21]	1	0.17	[0.15, 0.2]	1	0.18	[0.16, 0.21]	1	0.08	[0.05, 0.1]	1	0.17	[0.15, 0.2]	1	0.17	[0.15, 0.2]	1	0.02	[-0.01, 0.04]	0.87	0.01	[-0.02, 0.04]	0.731

	HDL			Triglycerides			Systolic BP			Diastolic BP			Obese (y/n)			Hypertension (y/n)			Pre-diabetes (y/n)			Diabetes (y/n)		
Predictor	Estimate	95% CrI	Pr(direction)	Estimate	95% CrI	Pr(direction)	Estimate	95% CrI	Pr(direction)	Estimate	95% CrI	Pr(direction)	Estimate	95% CrI	Pr(direction)	Estimate	95% CrI	Pr(direction)	Estimate	95% CrI	Pr(direction)	Estimate	95% CrI	Pr(direction)
Intercept	0.21	[0.17, 0.24]	1	-0.2	[-0.23, -0.17]	1	-0.05	[-0.08, -0.02]	1	0.03	[0, 0.07]	0.96	-1.07	[-1.16, -0.98]	1	0.14	[0.07, 0.22]	1	-2.37	[-2.5, -2.24]	1	-3.51	[-3.73, -3.29]	1
Age (z)	0.16	[0.13, 0.2]	1	0.13	[0.1, 0.17]	1	0.53	[0.49, 0.56]	1	0.29	[0.26, 0.33]	1	-0.2	[-0.29, -0.11]	1	0.68	[0.6, 0.77]	1	0.56	[0.44, 0.68]	1	0.63	[0.43, 0.81]	1
Age (z):Sex	0.05	[0, 0.1]	0.975	-0.15	[-0.2, -0.1]	1	-0.3	[-0.35, -0.26]	1	-0.14	[-0.19, -0.08]	1	0.1	[-0.05, 0.27]	0.904	-0.25	[-0.38, -0.13]	1	-0.08	[-0.28, 0.11]	0.788	-0.35	[-0.65, -0.04]	0.987
Sex	-0.42	[-0.48, -0.36]	1	0.33	[0.27, 0.38]	1	0.54	[0.49, 0.59]	1	0.16	[0.1, 0.22]	1	-1.05	[-1.24, -0.88]	1	0.51	[0.38, 0.64]	1	-0.25	[-0.51, 0]	0.972	-0.04	[-0.43, 0.34]	0.591
Smoking (y/n)	-0.2	[-0.26, -0.14]	1	0.2	[0.14, 0.26]	1	-0.33	[-0.38, -0.28]	1	-0.24	[-0.3, -0.18]	1	-0.53	[-0.7, -0.35]	1	-0.63	[-0.76, -0.5]	1	-0.4	[-0.63, -0.16]	1	-0.36	[-0.72, -0.01]	0.979
Inactivity (z)	-0.04	[-0.06, -0.01]	0.996	0.12	[0.1, 0.15]	1	0.04	[0.01, 0.06]	0.998	0.09	[0.06, 0.12]	1	0.55	[0.47, 0.63]	1	0.17	[0.1, 0.23]	1	0.09	[-0.01, 0.19]	0.957	0.19	[0.03, 0.35]	0.988

*Pr(direction) describes the proportion of the posterior that falls on the same side of 0 as the main association (i.e., a value of 0.96 can be interpreted as expressing 96% confidence that a given parameter is directionally greater or less than zero).

Physical activity predictor: Daily steps

	Weight			Body fat			Waist circumference			Waist:hip ratio			BMI			BRI			Total cholesterol			LDL		
Predictor	Estimate	95% CrI	Pr(direction)	Estimate	95% CrI	Pr(direction)	Estimate	95% CrI	Pr(direction)	Estimate	95% CrI	Pr(direction)	Estimate	95% CrI	Pr(direction)	Estimate	95% CrI	Pr(direction)	Estimate	95% CrI	Pr(direction)	Estimate	95% CrI	Pr(direction)
Intercept	-0.03	[-0.06, 0.01]	0.948	0.49	[0.46, 0.52]	1	0.13	[0.1, 0.17]	1	-0.05	[-0.08, -0.02]	0.997	0.17	[0.14, 0.21]	1	0.28	[0.25, 0.31]	1	0.12	[0.09, 0.16]	1	0.16	[0.12, 0.19]	1
Age (z)	-0.17	[-0.2, -0.14]	1	-0.01	[-0.04, 0.02]	0.804	0.09	[0.06, 0.12]	1	0.24	[0.21, 0.28]	1	-0.1	[-0.14, -0.07]	1	0.17	[0.14, 0.21]	1	0.24	[0.2, 0.27]	1	0.18	[0.14, 0.21]	1
Age (z):Sex	0.01	[-0.04, 0.07]	0.687	0.1	[0.06, 0.15]	1	0.01	[-0.04, 0.07]	0.707	-0.03	[-0.08, 0.03]	0.836	0.07	[0.01, 0.12]	0.992	0	[-0.05, 0.05]	0.514	-0.2	[-0.25, -0.14]	1	-0.11	[-0.16, -0.05]	1
Sex	0.19	[0.13, 0.24]	1	-1.14	[-1.19, -1.09]	1	-0.19	[-0.25, -0.13]	1	0.3	[0.24, 0.36]	1	-0.33	[-0.39, -0.27]	1	-0.56	[-0.61, -0.5]	1	-0.23	[-0.29, -0.17]	1	-0.27	[-0.33, -0.21]	1
Smoking (y/n)	-0.16	[-0.22, -0.1]	1	-0.27	[-0.31, -0.22]	1	-0.23	[-0.29, -0.17]	1	-0.21	[-0.27, -0.15]	1	-0.2	[-0.26, -0.15]	1	-0.26	[-0.31, -0.21]	1	-0.11	[-0.17, -0.06]	1	-0.17	[-0.23, -0.11]	1
Daily steps (z)	-0.16	[-0.19, -0.13]	1	-0.15	[-0.17, -0.12]	1	-0.15	[-0.17, -0.12]	1	-0.06	[-0.09, -0.04]	1	-0.14	[-0.17, -0.11]	1	-0.13	[-0.15, -0.1]	1	-0.07	[-0.1, -0.04]	1	-0.07	[-0.1, -0.04]	1

	HDL			Triglycerides			Systolic BP			Diastolic BP			Obese (y/n)			Hypertension (y/n)			Pre-diabetes (y/n)			Diabetes (y/n)		
Predictor	Estimate	95% CrI	Pr(direction)	Estimate	95% CrI	Pr(direction)	Estimate	95% CrI	Pr(direction)	Estimate	95% CrI	Pr(direction)	Estimate	95% CrI	Pr(direction)	Estimate	95% CrI	Pr(direction)	Estimate	95% CrI	Pr(direction)	Estimate	95% CrI	Pr(direction)
Intercept	0.21	[0.18, 0.25]	1	-0.21	[-0.24, -0.18]	1	-0.05	[-0.08, -0.02]	0.999	0.02	[-0.01, 0.06]	0.902	-1.12	[-1.2, -1.03]	1	0.13	[0.06, 0.21]	1	-2.36	[-2.49, -2.23]	1	-3.54	[-3.76, -3.33]	1
Age (z)	0.16	[0.13, 0.2]	1	0.13	[0.1, 0.16]	1	0.53	[0.5, 0.56]	1	0.29	[0.25, 0.32]	1	-0.22	[-0.3, -0.13]	1	0.69	[0.61, 0.77]	1	0.52	[0.4, 0.64]	1	0.6	[0.4, 0.79]	1
Age (z):Sex	0.05	[0, 0.1]	0.968	-0.15	[-0.2, -0.1]	1	-0.31	[-0.35, -0.26]	1	-0.13	[-0.18, -0.08]	1	0.1	[-0.06, 0.25]	0.886	-0.27	[-0.4, -0.15]	1	-0.06	[-0.25, 0.14]	0.732	-0.33	[-0.62, -0.03]	0.984
Sex	-0.44	[-0.49, -0.38]	1	0.36	[0.31, 0.42]	1	0.55	[0.5, 0.6]	1	0.2	[0.14, 0.26]	1	-0.86	[-1.04, -0.68]	1	0.6	[0.46, 0.72]	1	-0.24	[-0.5, 0.01]	0.97	0.02	[-0.36, 0.4]	0.553
Smoking (y/n)	-0.2	[-0.26, -0.14]	1	0.19	[0.14, 0.25]	1	-0.34	[-0.39, -0.28]	1	-0.24	[-0.3, -0.18]	1	-0.54	[-0.71, -0.37]	1	-0.64	[-0.78, -0.51]	1	-0.38	[-0.61, -0.16]	1	-0.34	[-0.7, 0.02]	0.969
Daily steps (z)	0.02	[0, 0.05]	0.951	-0.11	[-0.13, -0.08]	1	-0.04	[-0.06, -0.01]	0.999	-0.07	[-0.1, -0.04]	1	-0.52	[-0.61, -0.44]	1	-0.19	[-0.25, -0.13]	1	-0.1	[-0.21, 0]	0.974	-0.17	[-0.35, -0.01]	0.982

*Pr(direction) describes the proportion of the posterior that falls on the same side of 0 as the main association (i.e., a value of 0.96 can be interpreted as expressing 96% confidence that a given parameter is directionally greater or less than zero).

Physical activity predictor: Intensity gradient

	Weight			Body fat			Waist circumference			Waist:hip ratio			BMI			BRI			Total cholesterol			LDL		
Predictor	Estimate	95% CrI	Pr(direction)	Estimate	95% CrI	Pr(direction)	Estimate	95% CrI	Pr(direction)	Estimate	95% CrI	Pr(direction)	Estimate	95% CrI	Pr(direction)	Estimate	95% CrI	Pr(direction)	Estimate	95% CrI	Pr(direction)	Estimate	95% CrI	Pr(direction)
Intercept	-0.06	[-0.1, -0.03]	1	0.45	[0.42, 0.48]	1	0.1	[0.06, 0.13]	1	-0.05	[-0.09, -0.02]	0.998	0.14	[0.11, 0.18]	1	0.25	[0.21, 0.28]	1	0.12	[0.08, 0.16]	1	0.15	[0.11, 0.18]	1
Age (z)	-0.19	[-0.23, -0.16]	1	-0.04	[-0.07, -0.01]	0.995	0.06	[0.03, 0.1]	0.999	0.25	[0.21, 0.28]	1	-0.12	[-0.16, -0.09]	1	0.15	[0.11, 0.18]	1	0.24	[0.2, 0.28]	1	0.17	[0.13, 0.21]	1
Age (z):Sex	0.01	[-0.04, 0.06]	0.653	0.1	[0.06, 0.14]	1	0.01	[-0.04, 0.06]	0.642	-0.01	[-0.07, 0.04]	0.702	0.07	[0.01, 0.12]	0.995	0	[-0.06, 0.05]	0.577	-0.19	[-0.24, -0.13]	1	-0.1	[-0.16, -0.05]	1
Sex	0.26	[0.19, 0.32]	1	-1.07	[-1.12, -1.01]	1	-0.12	[-0.18, -0.05]	1	0.29	[0.23, 0.35]	1	-0.27	[-0.33, -0.21]	1	-0.49	[-0.55, -0.43]	1	-0.23	[-0.3, -0.17]	1	-0.26	[-0.32, -0.19]	1
Smoking (y/n)	-0.13	[-0.19, -0.07]	1	-0.24	[-0.29, -0.19]	1	-0.21	[-0.27, -0.15]	1	-0.2	[-0.26, -0.15]	1	-0.18	[-0.24, -0.12]	1	-0.24	[-0.29, -0.18]	1	-0.1	[-0.16, -0.04]	1	-0.16	[-0.22, -0.1]	1
Intensity gradient (z)	-0.14	[-0.17, -0.11]	1	-0.14	[-0.17, -0.11]	1	-0.15	[-0.18, -0.11]	1	-0.01	[-0.04, 0.03]	0.676	-0.12	[-0.15, -0.09]	1	-0.13	[-0.17, -0.1]	1	-0.02	[-0.05, 0.02]	0.838	-0.04	[-0.08, -0.01]	0.992

	HDL			Triglycerides			Systolic BP			Diastolic BP			Obese (y/n)			Hypertension (y/n)			Pre-diabetes (y/n)			Diabetes (y/n)		
Predictor	Estimate	95% CrI	Pr(direction)	Estimate	95% CrI	Pr(direction)	Estimate	95% CrI	Pr(direction)	Estimate	95% CrI	Pr(direction)	Estimate	95% CrI	Pr(direction)	Estimate	95% CrI	Pr(direction)	Estimate	95% CrI	Pr(direction)	Estimate	95% CrI	Pr(direction)
Intercept	0.23	[0.19, 0.26]	1	-0.23	[-0.26, -0.19]	1	-0.05	[-0.09, -0.02]	0.999	0	[-0.04, 0.04]	0.516	-1.21	[-1.3, -1.12]	1	0.1	[0.02, 0.17]	0.993	-2.44	[-2.58, -2.3]	1	-3.65	[-3.89, -3.43]	1
Age (z)	0.17	[0.14, 0.21]	1	0.12	[0.08, 0.15]	1	0.53	[0.49, 0.56]	1	0.27	[0.24, 0.31]	1	-0.29	[-0.38, -0.2]	1	0.67	[0.58, 0.75]	1	0.45	[0.32, 0.58]	1	0.5	[0.31, 0.7]	1
Age (z):Sex	0.05	[0, 0.11]	0.981	-0.15	[-0.2, -0.1]	1	-0.3	[-0.35, -0.26]	1	-0.13	[-0.19, -0.08]	1	0.1	[-0.06, 0.25]	0.886	-0.27	[-0.39, -0.14]	1	-0.1	[-0.29, 0.09]	0.842	-0.38	[-0.67, -0.08]	0.993
Sex	-0.47	[-0.53, -0.4]	1	0.39	[0.33, 0.46]	1	0.56	[0.5, 0.62]	1	0.24	[0.18, 0.31]	1	-0.62	[-0.81, -0.43]	1	0.65	[0.51, 0.79]	1	-0.09	[-0.36, 0.18]	0.738	0.23	[-0.18, 0.63]	0.864
Smoking (y/n)	-0.2	[-0.26, -0.14]	1	0.21	[0.15, 0.27]	1	-0.33	[-0.39, -0.28]	1	-0.23	[-0.29, -0.17]	1	-0.46	[-0.63, -0.29]	1	-0.61	[-0.74, -0.48]	1	-0.36	[-0.59, -0.13]	0.999	-0.3	[-0.66, 0.05]	0.953
Intensity gradient (z)	0.05	[0.02, 0.08]	0.998	-0.08	[-0.11, -0.05]	1	-0.02	[-0.05, 0.01]	0.919	-0.08	[-0.12, -0.05]	1	-0.46	[-0.56, -0.37]	1	-0.13	[-0.21, -0.06]	1	-0.25	[-0.38, -0.13]	1	-0.36	[-0.54, -0.18]	1

*Pr(direction) describes the proportion of the posterior that falls on the same side of 0 as the main association (i.e., a value of 0.96 can be interpreted as expressing 96% confidence that a given parameter is directionally greater or less than zero).

Physical activity predictor: Light activity

	Weight			Body fat			Waist circumference			Waist:hip ratio			BMI			BRI			Total cholesterol			LDL		
Predictor	Estimate	95% CrI	Pr(direction)	Estimate	95% CrI	Pr(direction)	Estimate	95% CrI	Pr(direction)	Estimate	95% CrI	Pr(direction)	Estimate	95% CrI	Pr(direction)	Estimate	95% CrI	Pr(direction)	Estimate	95% CrI	Pr(direction)	Estimate	95% CrI	Pr(direction)
Intercept	-0.02	[-0.05, 0.02]	0.84	0.51	[0.48, 0.54]	1	0.14	[0.11, 0.18]	1	-0.05	[-0.08, -0.01]	0.994	0.18	[0.15, 0.22]	1	0.29	[0.25, 0.32]	1	0.15	[0.12, 0.19]	1	0.18	[0.15, 0.22]	1
Age (z)	-0.12	[-0.15, -0.08]	1	0.03	[0.01, 0.06]	0.991	0.13	[0.09, 0.17]	1	0.25	[0.22, 0.29]	1	-0.06	[-0.09, -0.02]	1	0.21	[0.17, 0.24]	1	0.28	[0.24, 0.31]	1	0.2	[0.17, 0.24]	1
Age (z):Sex	0.03	[-0.02, 0.08]	0.864	0.12	[0.08, 0.17]	1	0.04	[-0.01, 0.09]	0.941	-0.01	[-0.06, 0.04]	0.639	0.08	[0.03, 0.14]	1	0.03	[-0.03, 0.08]	0.844	-0.2	[-0.25, -0.14]	1	-0.1	[-0.15, -0.05]	1
Sex	0.12	[0.06, 0.18]	1	-1.21	[-1.26, -1.16]	1	-0.26	[-0.32, -0.2]	1	0.28	[0.22, 0.34]	1	-0.39	[-0.45, -0.34]	1	-0.61	[-0.67, -0.55]	1	-0.28	[-0.34, -0.22]	1	-0.31	[-0.37, -0.25]	1
Smoking (y/n)	-0.15	[-0.21, -0.09]	1	-0.26	[-0.31, -0.21]	1	-0.23	[-0.28, -0.17]	1	-0.21	[-0.27, -0.15]	1	-0.2	[-0.25, -0.14]	1	-0.25	[-0.31, -0.2]	1	-0.13	[-0.19, -0.07]	1	-0.18	[-0.25, -0.13]	1
Light activity (z)	-0.09	[-0.11, -0.06]	1	-0.1	[-0.12, -0.08]	1	-0.08	[-0.1, -0.05]	1	-0.01	[-0.04, 0.02]	0.832	-0.08	[-0.1, -0.05]	1	-0.06	[-0.09, -0.04]	1	-0.08	[-0.11, -0.05]	1	-0.04	[-0.07, -0.01]	0.994

	HDL			Triglycerides			Systolic BP			Diastolic BP			Obese (y/n)			Hypertension (y/n)			Pre-diabetes (y/n)			Diabetes (y/n)		
Predictor	Estimate	95% CrI	Pr(direction)	Estimate	95% CrI	Pr(direction)	Estimate	95% CrI	Pr(direction)	Estimate	95% CrI	Pr(direction)	Estimate	95% CrI	Pr(direction)	Estimate	95% CrI	Pr(direction)	Estimate	95% CrI	Pr(direction)	Estimate	95% CrI	Pr(direction)
Intercept	0.22	[0.19, 0.25]	1	-0.2	[-0.23, -0.16]	1	-0.05	[-0.08, -0.02]	0.998	0.03	[-0.01, 0.06]	0.933	-1.03	[-1.12, -0.95]	1	0.14	[0.06, 0.21]	1	-2.39	[-2.53, -2.26]	1	-3.56	[-3.8, -3.34]	1
Age (z)	0.17	[0.14, 0.21]	1	0.17	[0.13, 0.2]	1	0.54	[0.51, 0.57]	1	0.31	[0.27, 0.35]	1	-0.07	[-0.16, 0.01]	0.953	0.72	[0.63, 0.8]	1	0.56	[0.43, 0.68]	1	0.62	[0.42, 0.8]	1
Age (z):Sex	0.04	[-0.01, 0.09]	0.92	-0.14	[-0.19, -0.09]	1	-0.3	[-0.35, -0.25]	1	-0.13	[-0.18, -0.07]	1	0.17	[0.02, 0.33]	0.983	-0.24	[-0.36, -0.11]	1	-0.05	[-0.25, 0.15]	0.697	-0.29	[-0.57, 0]	0.975
Sex	-0.45	[-0.5, -0.39]	1	0.31	[0.25, 0.36]	1	0.53	[0.48, 0.58]	1	0.17	[0.11, 0.23]	1	-1.02	[-1.21, -0.83]	1	0.5	[0.37, 0.63]	1	-0.21	[-0.47, 0.03]	0.951	0.05	[-0.34, 0.43]	0.589
Smoking (y/n)	-0.21	[-0.26, -0.15]	1	0.2	[0.14, 0.25]	1	-0.33	[-0.38, -0.28]	1	-0.23	[-0.3, -0.17]	1	-0.49	[-0.67, -0.3]	1	-0.62	[-0.75, -0.49]	1	-0.37	[-0.61, -0.14]	0.999	-0.3	[-0.66, 0.05]	0.953
Light activity (z)	-0.04	[-0.07, -0.01]	0.998	-0.09	[-0.11, -0.06]	1	-0.03	[-0.06, 0]	0.99	-0.02	[-0.05, 0]	0.951	-0.21	[-0.28, -0.13]	1	-0.08	[-0.14, -0.02]	0.993	0.07	[-0.04, 0.18]	0.891	0.16	[-0.01, 0.33]	0.97

*Pr(direction) describes the proportion of the posterior that falls on the same side of 0 as the main association (i.e., a value of 0.96 can be interpreted as expressing 96% confidence that a given parameter is directionally greater or less than zero).

Physical activity predictor: Moderate activity

	Weight			Body fat			Waist circumference			Waist:hip ratio			BMI			BRI			Total cholesterol			LDL		
Predictor	Estimate	95% CrI	Pr(direction)	Estimate	95% CrI	Pr(direction)	Estimate	95% CrI	Pr(direction)	Estimate	95% CrI	Pr(direction)	Estimate	95% CrI	Pr(direction)	Estimate	95% CrI	Pr(direction)	Estimate	95% CrI	Pr(direction)	Estimate	95% CrI	Pr(direction)
Intercept	-0.04	[-0.07, 0]	0.978	0.49	[0.46, 0.52]	1	0.13	[0.1, 0.17]	1	-0.05	[-0.08, -0.01]	0.998	0.17	[0.13, 0.2]	1	0.28	[0.24, 0.31]	1	0.14	[0.1, 0.17]	1	0.18	[0.14, 0.21]	1
Age (z)	-0.17	[-0.2, -0.13]	1	-0.02	[-0.05, 0.01]	0.911	0.08	[0.05, 0.12]	1	0.24	[0.2, 0.27]	1	-0.1	[-0.13, -0.06]	1	0.17	[0.13, 0.2]	1	0.25	[0.21, 0.28]	1	0.18	[0.14, 0.22]	1
Age (z):Sex	0.03	[-0.02, 0.08]	0.864	0.12	[0.08, 0.17]	1	0.04	[-0.02, 0.09]	0.907	-0.02	[-0.07, 0.03]	0.758	0.08	[0.03, 0.13]	1	0.02	[-0.03, 0.07]	0.781	-0.19	[-0.24, -0.14]	1	-0.1	[-0.16, -0.05]	1
Sex	0.17	[0.11, 0.23]	1	-1.15	[-1.19, -1.1]	1	-0.2	[-0.26, -0.14]	1	0.3	[0.24, 0.36]	1	-0.34	[-0.4, -0.28]	1	-0.57	[-0.62, -0.51]	1	-0.24	[-0.29, -0.18]	1	-0.28	[-0.34, -0.22]	1
Smoking (y/n)	-0.15	[-0.21, -0.09]	1	-0.27	[-0.31, -0.22]	1	-0.23	[-0.29, -0.17]	1	-0.21	[-0.27, -0.16]	1	-0.2	[-0.26, -0.14]	1	-0.26	[-0.32, -0.2]	1	-0.12	[-0.18, -0.06]	1	-0.19	[-0.25, -0.13]	1
Moderate activity (z)	-0.09	[-0.12, -0.06]	1	-0.1	[-0.13, -0.08]	1	-0.1	[-0.12, -0.07]	1	-0.05	[-0.08, -0.03]	1	-0.08	[-0.1, -0.05]	1	-0.09	[-0.12, -0.06]	1	-0.03	[-0.06, 0]	0.988	-0.05	[-0.08, -0.02]	0.999

	HDL			Triglycerides			Systolic BP			Diastolic BP			Obese (y/n)			Hypertension (y/n)			Pre-diabetes (y/n)			Diabetes (y/n)		
Predictor	Estimate	95% CrI	Pr(direction)	Estimate	95% CrI	Pr(direction)	Estimate	95% CrI	Pr(direction)	Estimate	95% CrI	Pr(direction)	Estimate	95% CrI	Pr(direction)	Estimate	95% CrI	Pr(direction)	Estimate	95% CrI	Pr(direction)	Estimate	95% CrI	Pr(direction)
Intercept	0.21	[0.18, 0.25]	1	-0.21	[-0.25, -0.18]	1	-0.05	[-0.08, -0.02]	0.999	0.02	[-0.01, 0.06]	0.919	-1.09	[-1.18, -1.01]	1	0.13	[0.05, 0.21]	1	-2.38	[-2.51, -2.25]	1	-3.53	[-3.75, -3.31]	1
Age (z)	0.18	[0.14, 0.21]	1	0.12	[0.09, 0.16]	1	0.52	[0.49, 0.55]	1	0.28	[0.25, 0.32]	1	-0.23	[-0.33, -0.14]	1	0.65	[0.57, 0.73]	1	0.54	[0.41, 0.67]	1	0.57	[0.37, 0.76]	1
Age (z):Sex	0.05	[0, 0.11]	0.977	-0.14	[-0.19, -0.09]	1	-0.31	[-0.35, -0.26]	1	-0.14	[-0.19, -0.08]	1	0.13	[-0.03, 0.3]	0.944	-0.26	[-0.38, -0.13]	1	-0.09	[-0.29, 0.12]	0.816	-0.36	[-0.65, -0.07]	0.992
Sex	-0.44	[-0.5, -0.38]	1	0.37	[0.31, 0.42]	1	0.56	[0.5, 0.61]	1	0.19	[0.13, 0.26]	1	-0.87	[-1.04, -0.69]	1	0.59	[0.46, 0.71]	1	-0.21	[-0.46, 0.05]	0.948	0.04	[-0.35, 0.41]	0.586
Smoking (y/n)	-0.19	[-0.25, -0.14]	1	0.2	[0.14, 0.25]	1	-0.33	[-0.39, -0.28]	1	-0.24	[-0.3, -0.18]	1	-0.52	[-0.7, -0.34]	1	-0.65	[-0.78, -0.52]	1	-0.4	[-0.63, -0.18]	1	-0.38	[-0.75, -0.03]	0.981
Moderate activity (z)	0.06	[0.03, 0.09]	1	-0.07	[-0.1, -0.04]	1	-0.05	[-0.07, -0.02]	1	-0.08	[-0.11, -0.06]	1	-0.38	[-0.46, -0.3]	1	-0.21	[-0.27, -0.15]	1	-0.11	[-0.21, -0.01]	0.983	-0.26	[-0.43, -0.09]	0.999

*Pr(direction) describes the proportion of the posterior that falls on the same side of 0 as the main association (i.e., a value of 0.96 can be interpreted as expressing 96% confidence that a given parameter is directionally greater or less than zero).

Physical activity predictor: Vigorous activity

	Weight			Body fat			Waist circumference			Waist:hip ratio			BMI			BRI			Total cholesterol			LDL		
Predictor	Estimate	95% CrI	Pr(direction)	Estimate	95% CrI	Pr(direction)	Estimate	95% CrI	Pr(direction)	Estimate	95% CrI	Pr(direction)	Estimate	95% CrI	Pr(direction)	Estimate	95% CrI	Pr(direction)	Estimate	95% CrI	Pr(direction)	Estimate	95% CrI	Pr(direction)
Intercept	-0.08	[-0.12, -0.04]	1	0.45	[0.42, 0.48]	1	0.09	[0.05, 0.12]	1	-0.06	[-0.1, -0.03]	1	0.13	[0.1, 0.17]	1	0.24	[0.21, 0.27]	1	0.13	[0.09, 0.16]	1	0.16	[0.13, 0.2]	1
Age (z)	-0.19	[-0.22, -0.15]	1	-0.03	[-0.07, 0]	0.986	0.07	[0.03, 0.1]	1	0.24	[0.2, 0.27]	1	-0.12	[-0.15, -0.08]	1	0.15	[0.12, 0.18]	1	0.24	[0.21, 0.28]	1	0.18	[0.14, 0.22]	1
Age (z):Sex	0	[-0.05, 0.06]	0.536	0.1	[0.06, 0.15]	1	0.01	[-0.04, 0.06]	0.69	-0.02	[-0.07, 0.03]	0.78	0.06	[0.01, 0.11]	0.991	0	[-0.05, 0.05]	0.505	-0.19	[-0.24, -0.14]	1	-0.1	[-0.16, -0.05]	1
Sex	0.28	[0.22, 0.35]	1	-1.05	[-1.1, -1]	1	-0.1	[-0.16, -0.03]	0.998	0.33	[0.27, 0.39]	1	-0.25	[-0.31, -0.19]	1	-0.47	[-0.53, -0.42]	1	-0.22	[-0.28, -0.15]	1	-0.25	[-0.31, -0.18]	1
Smoking (y/n)	-0.13	[-0.19, -0.07]	1	-0.25	[-0.3, -0.2]	1	-0.21	[-0.27, -0.15]	1	-0.2	[-0.26, -0.15]	1	-0.18	[-0.24, -0.12]	1	-0.24	[-0.3, -0.19]	1	-0.12	[-0.18, -0.06]	1	-0.18	[-0.24, -0.12]	1
Vigorous activity (z)	-0.17	[-0.2, -0.14]	1	-0.16	[-0.18, -0.13]	1	-0.17	[-0.2, -0.14]	1	-0.05	[-0.08, -0.03]	1	-0.14	[-0.17, -0.12]	1	-0.15	[-0.18, -0.12]	1	-0.04	[-0.07, -0.01]	0.993	-0.06	[-0.09, -0.02]	1

	HDL			Triglycerides			Systolic BP			Diastolic BP			Obese (y/n)			Hypertension (y/n)			Pre-diabetes (y/n)			Diabetes (y/n)		
Predictor	Estimate	95% CrI	Pr(direction)	Estimate	95% CrI	Pr(direction)	Estimate	95% CrI	Pr(direction)	Estimate	95% CrI	Pr(direction)	Estimate	95% CrI	Pr(direction)	Estimate	95% CrI	Pr(direction)	Estimate	95% CrI	Pr(direction)	Estimate	95% CrI	Pr(direction)
Intercept	0.23	[0.2, 0.27]	1	-0.24	[-0.27, -0.2]	1	-0.05	[-0.09, -0.02]	1	0.01	[-0.03, 0.04]	0.641	-1.23	[-1.32, -1.14]	1	0.09	[0.01, 0.16]	0.989	-2.45	[-2.59, -2.32]	1	-3.56	[-3.79, -3.33]	1
Age (z)	0.18	[0.15, 0.22]	1	0.11	[0.08, 0.15]	1	0.53	[0.5, 0.56]	1	0.29	[0.25, 0.32]	1	-0.27	[-0.36, -0.18]	1	0.66	[0.58, 0.75]	1	0.5	[0.38, 0.63]	1	0.6	[0.4, 0.8]	1
Age (z):Sex	0.06	[0.01, 0.11]	0.992	-0.15	[-0.2, -0.1]	1	-0.3	[-0.34, -0.25]	1	-0.14	[-0.19, -0.08]	1	0.1	[-0.06, 0.25]	0.88	-0.26	[-0.38, -0.14]	1	-0.13	[-0.32, 0.06]	0.921	-0.35	[-0.66, -0.04]	0.987
Sex	-0.49	[-0.55, -0.43]	1	0.43	[0.37, 0.49]	1	0.54	[0.49, 0.6]	1	0.22	[0.16, 0.29]	1	-0.6	[-0.78, -0.41]	1	0.65	[0.51, 0.78]	1	-0.05	[-0.33, 0.21]	0.636	0.09	[-0.3, 0.5]	0.663
Smoking (y/n)	-0.2	[-0.26, -0.15]	1	0.21	[0.15, 0.27]	1	-0.33	[-0.38, -0.28]	1	-0.23	[-0.29, -0.17]	1	-0.45	[-0.63, -0.28]	1	-0.61	[-0.74, -0.48]	1	-0.38	[-0.6, -0.15]	1	-0.33	[-0.69, 0.02]	0.97
Vigorous activity (z)	0.08	[0.05, 0.11]	1	-0.11	[-0.14, -0.08]	1	0	[-0.03, 0.03]	0.563	-0.06	[-0.09, -0.03]	1	-0.51	[-0.6, -0.42]	1	-0.15	[-0.21, -0.08]	1	-0.26	[-0.38, -0.14]	1	-0.17	[-0.36, 0.01]	0.963

*Pr(direction) describes the proportion of the posterior that falls on the same side of 0 as the main association (i.e., a value of 0.96 can be interpreted as expressing 96% confidence that a given parameter is directionally greater or less than zero).

Physical activity predictor: ENMO

	Weight			Body fat			Waist circumference			Waist:hip ratio			BMI			BRI			Total cholesterol			LDL		
Predictor	Estimate	95% CrI	Pr(direction)	Estimate	95% CrI	Pr(direction)	Estimate	95% CrI	Pr(direction)	Estimate	95% CrI	Pr(direction)	Estimate	95% CrI	Pr(direction)	Estimate	95% CrI	Pr(direction)	Estimate	95% CrI	Pr(direction)	Estimate	95% CrI	Pr(direction)
Intercept	-0.03	[-0.07, 0]	0.972	0.48	[0.46, 0.51]	1	0.13	[0.09, 0.16]	1	-0.05	[-0.08, -0.02]	0.998	0.17	[0.14, 0.2]	1	0.28	[0.24, 0.31]	1	0.12	[0.09, 0.16]	1	0.15	[0.12, 0.19]	1
Age (z)	-0.16	[-0.2, -0.13]	1	-0.01	[-0.04, 0.02]	0.739	0.09	[0.06, 0.13]	1	0.24	[0.21, 0.28]	1	-0.1	[-0.13, -0.06]	1	0.18	[0.14, 0.21]	1	0.24	[0.2, 0.27]	1	0.18	[0.14, 0.21]	1
Age (z):Sex	0.01	[-0.05, 0.06]	0.584	0.1	[0.05, 0.14]	1	0.01	[-0.04, 0.06]	0.663	-0.03	[-0.08, 0.02]	0.85	0.06	[0.01, 0.11]	0.988	0	[-0.05, 0.05]	0.555	-0.2	[-0.26, -0.15]	1	-0.12	[-0.17, -0.06]	1
Sex	0.19	[0.13, 0.25]	1	-1.13	[-1.18, -1.08]	1	-0.19	[-0.25, -0.13]	1	0.3	[0.24, 0.36]	1	-0.33	[-0.38, -0.27]	1	-0.55	[-0.61, -0.5]	1	-0.22	[-0.28, -0.16]	1	-0.26	[-0.32, -0.2]	1
Smoking (y/n)	-0.15	[-0.21, -0.1]	1	-0.26	[-0.31, -0.21]	1	-0.23	[-0.29, -0.17]	1	-0.21	[-0.27, -0.15]	1	-0.2	[-0.26, -0.14]	1	-0.26	[-0.31, -0.2]	1	-0.11	[-0.17, -0.05]	1	-0.17	[-0.23, -0.11]	1
ENMO (z)	-0.14	[-0.17, -0.12]	1	-0.13	[-0.16, -0.11]	1	-0.13	[-0.16, -0.11]	1	-0.06	[-0.08, -0.03]	1	-0.13	[-0.15, -0.1]	1	-0.12	[-0.14, -0.09]	1	-0.08	[-0.11, -0.05]	1	-0.08	[-0.11, -0.05]	1

	HDL			Triglycerides			Systolic BP			Diastolic BP			Obese (y/n)			Hypertension (y/n)			Pre-diabetes (y/n)			Diabetes (y/n)		
Predictor	Estimate	95% CrI	Pr(direction)	Estimate	95% CrI	Pr(direction)	Estimate	95% CrI	Pr(direction)	Estimate	95% CrI	Pr(direction)	Estimate	95% CrI	Pr(direction)	Estimate	95% CrI	Pr(direction)	Estimate	95% CrI	Pr(direction)	Estimate	95% CrI	Pr(direction)
Intercept	0.22	[0.18, 0.25]	1	-0.21	[-0.25, -0.18]	1	-0.05	[-0.08, -0.02]	0.999	0.02	[-0.02, 0.05]	0.856	-1.12	[-1.21, -1.03]	1	0.13	[0.05, 0.2]	0.999	-2.37	[-2.51, -2.25]	1	-3.55	[-3.79, -3.34]	1
Age (z)	0.16	[0.13, 0.2]	1	0.13	[0.1, 0.16]	1	0.53	[0.5, 0.56]	1	0.29	[0.25, 0.32]	1	-0.19	[-0.28, -0.11]	1	0.69	[0.61, 0.78]	1	0.51	[0.39, 0.63]	1	0.61	[0.42, 0.79]	1
Age (z):Sex	0.05	[0, 0.11]	0.978	-0.16	[-0.21, -0.11]	1	-0.31	[-0.35, -0.26]	1	-0.13	[-0.19, -0.08]	1	0.08	[-0.08, 0.23]	0.841	-0.29	[-0.41, -0.17]	1	-0.07	[-0.26, 0.12]	0.783	-0.33	[-0.62, -0.04]	0.985
Sex	-0.44	[-0.5, -0.38]	1	0.37	[0.31, 0.43]	1	0.55	[0.5, 0.6]	1	0.21	[0.14, 0.27]	1	-0.84	[-1.02, -0.67]	1	0.61	[0.48, 0.74]	1	-0.22	[-0.48, 0.01]	0.965	0.03	[-0.38, 0.42]	0.561
Smoking (y/n)	-0.2	[-0.25, -0.14]	1	0.19	[0.14, 0.25]	1	-0.34	[-0.39, -0.29]	1	-0.24	[-0.3, -0.18]	1	-0.54	[-0.71, -0.36]	1	-0.64	[-0.77, -0.51]	1	-0.39	[-0.61, -0.17]	1	-0.33	[-0.7, 0.04]	0.962
ENMO (z)	0.04	[0.01, 0.06]	0.997	-0.11	[-0.14, -0.09]	1	-0.03	[-0.05, 0]	0.989	-0.08	[-0.11, -0.05]	1	-0.5	[-0.58, -0.41]	1	-0.2	[-0.26, -0.14]	1	-0.16	[-0.27, -0.04]	0.998	-0.17	[-0.35, 0.01]	0.964

*Pr(direction) describes the proportion of the posterior that falls on the same side of 0 as the main association (i.e., a value of 0.96 can be interpreted as expressing 96% confidence that a given parameter is directionally greater or less than zero).

Physical activity predictor: DFA

	Weight			Body fat			Waist circumference			Waist:hip ratio			BMI			BRI			Total cholesterol			LDL		
Predictor	Estimate	95% CrI	Pr(direction)	Estimate	95% CrI	Pr(direction)	Estimate	95% CrI	Pr(direction)	Estimate	95% CrI	Pr(direction)	Estimate	95% CrI	Pr(direction)	Estimate	95% CrI	Pr(direction)	Estimate	95% CrI	Pr(direction)	Estimate	95% CrI	Pr(direction)
Intercept	0	[-0.04, 0.03]	0.53	0.52	[0.49, 0.55]	1	0.15	[0.12, 0.19]	1	-0.05	[-0.08, -0.01]	0.998	0.2	[0.17, 0.24]	1	0.3	[0.27, 0.33]	1	0.12	[0.09, 0.16]	1	0.16	[0.12, 0.19]	1
Age (z)	-0.14	[-0.17, -0.1]	1	0.02	[-0.01, 0.05]	0.89	0.12	[0.08, 0.15]	1	0.25	[0.21, 0.28]	1	-0.07	[-0.1, -0.04]	1	0.2	[0.16, 0.23]	1	0.24	[0.21, 0.28]	1	0.18	[0.15, 0.22]	1
Age (z):Sex	0.04	[-0.02, 0.09]	0.922	0.13	[0.08, 0.17]	1	0.04	[-0.01, 0.09]	0.946	-0.02	[-0.07, 0.04]	0.721	0.09	[0.04, 0.14]	1	0.03	[-0.02, 0.08]	0.854	-0.18	[-0.23, -0.13]	1	-0.09	[-0.15, -0.04]	0.999
Sex	0.09	[0.03, 0.15]	0.998	-1.24	[-1.29, -1.19]	1	-0.27	[-0.33, -0.21]	1	0.29	[0.23, 0.35]	1	-0.43	[-0.49, -0.38]	1	-0.63	[-0.69, -0.58]	1	-0.24	[-0.3, -0.18]	1	-0.29	[-0.35, -0.22]	1
Smoking (y/n)	-0.15	[-0.21, -0.09]	1	-0.26	[-0.31, -0.21]	1	-0.22	[-0.28, -0.17]	1	-0.21	[-0.26, -0.15]	1	-0.2	[-0.25, -0.14]	1	-0.25	[-0.31, -0.2]	1	-0.1	[-0.16, -0.04]	1	-0.16	[-0.22, -0.1]	1
DFA (z)	-0.12	[-0.15, -0.09]	1	-0.12	[-0.14, -0.1]	1	-0.08	[-0.11, -0.05]	1	0	[-0.03, 0.03]	0.507	-0.13	[-0.16, -0.11]	1	-0.09	[-0.11, -0.06]	1	0.01	[-0.02, 0.04]	0.787	0	[-0.03, 0.03]	0.534

	HDL			Triglycerides			Systolic BP			Diastolic BP			Obese (y/n)			Hypertension (y/n)			Pre-diabetes (y/n)			Diabetes (y/n)		
Predictor	Estimate	95% CrI	Pr(direction)	Estimate	95% CrI	Pr(direction)	Estimate	95% CrI	Pr(direction)	Estimate	95% CrI	Pr(direction)	Estimate	95% CrI	Pr(direction)	Estimate	95% CrI	Pr(direction)	Estimate	95% CrI	Pr(direction)	Estimate	95% CrI	Pr(direction)
Intercept	0.21	[0.17, 0.24]	1	-0.19	[-0.23, -0.16]	1	-0.05	[-0.09, -0.02]	1	0.03	[-0.01, 0.06]	0.941	-1.01	[-1.09, -0.93]	1	0.14	[0.06, 0.21]	1	-2.32	[-2.46, -2.2]	1	-3.51	[-3.74, -3.28]	1
Age (z)	0.16	[0.12, 0.19]	1	0.15	[0.12, 0.18]	1	0.53	[0.5, 0.56]	1	0.3	[0.26, 0.33]	1	-0.1	[-0.19, -0.02]	0.99	0.71	[0.63, 0.79]	1	0.56	[0.44, 0.68]	1	0.65	[0.46, 0.83]	1
Age (z):Sex	0.05	[0, 0.1]	0.97	-0.13	[-0.18, -0.08]	1	-0.31	[-0.35, -0.26]	1	-0.12	[-0.17, -0.07]	1	0.19	[0.03, 0.35]	0.989	-0.25	[-0.38, -0.13]	1	-0.03	[-0.23, 0.16]	0.641	-0.3	[-0.6, 0]	0.974
Sex	-0.43	[-0.48, -0.37]	1	0.3	[0.24, 0.36]	1	0.57	[0.52, 0.62]	1	0.18	[0.12, 0.24]	1	-1.15	[-1.34, -0.97]	1	0.55	[0.42, 0.68]	1	-0.43	[-0.71, -0.18]	1	-0.11	[-0.52, 0.29]	0.713
Smoking (y/n)	-0.19	[-0.26, -0.14]	1	0.2	[0.14, 0.26]	1	-0.34	[-0.39, -0.28]	1	-0.24	[-0.3, -0.18]	1	-0.5	[-0.68, -0.33]	1	-0.63	[-0.76, -0.5]	1	-0.39	[-0.62, -0.15]	0.999	-0.32	[-0.67, 0.02]	0.966
DFA (z)	0.02	[-0.01, 0.05]	0.932	-0.07	[-0.1, -0.04]	1	0.04	[0.01, 0.06]	0.998	-0.02	[-0.05, 0.01]	0.879	-0.36	[-0.43, -0.28]	1	-0.02	[-0.08, 0.04]	0.738	-0.29	[-0.39, -0.19]	1	-0.17	[-0.33, -0.01]	0.982

*Pr(direction) describes the proportion of the posterior that falls on the same side of 0 as the main association (i.e., a value of 0.96 can be interpreted as expressing 96% confidence that a given parameter is directionally greater or less than zero).
