# Supplementary material for: Physical activity and cardiometabolic health across an extreme lifestyle gradient
Source: Evol Med Public Health. 2026 Jul 21;14(1):1–15. doi: 10.1093/emph/eoag017 (PMC13489160; doi:10.1093/emph/eoag017)
Supplement: Supplementary file 1 [file supplementary_material_eoag017.zip › TabS5_bayes_2026_05_21.pdf]

### Physical activity predictor: Inactivity; adjusted for urbanicity

| | Weight | | | Body fat | | | Waist circumference | | | Waist:hip ratio | | | BMI | | | BRI | | | Total cholesterol | | | LDL | | |
|---|---|---|---|---|---|---|---|---|---|---|---|---|---|---|---|---|---|---|---|---|---|---|---|---|
| Predictor | Estimate | 95% CrI | Pr(direction) | Estimate | 95% CrI | Pr(direction) | Estimate | 95% CrI | Pr(direction) | Estimate | 95% CrI | Pr(direction) | Estimate | 95% CrI | Pr(direction) | Estimate | 95% CrI | Pr(direction) | Estimate | 95% CrI | Pr(direction) | Estimate | 95% CrI | Pr(direction) |
| Intercept | -0.04 | [-0.07, -0.01] | 0.994 | 0.49 | [0.46, 0.52] | 1 | 0.12 | [0.09, 0.15] | 1 | -0.04 | [-0.08, -0.01] | 0.993 | 0.17 | [0.13, 0.2] | 1 | 0.27 | [0.24, 0.3] | 1 | 0.13 | [0.09, 0.16] | 1 | 0.17 | [0.14, 0.21] | 1 |
| Age (z) | -0.24 | [-0.27, -0.2] | 1 | -0.06 | [-0.09, -0.03] | 1 | 0.04 | [0, 0.07] | 0.985 | 0.24 | [0.2, 0.27] | 1 | -0.16 | [-0.2, -0.13] | 1 | 0.13 | [0.09, 0.16] | 1 | 0.23 | [0.2, 0.27] | 1 | 0.18 | [0.15, 0.22] | 1 |
| Age (z):Sex | 0.01 | [-0.04, 0.06] | 0.628 | 0.11 | [0.06, 0.15] | 1 | 0.01 | [-0.04, 0.06] | 0.671 | -0.03 | [-0.08, 0.03] | 0.827 | 0.06 | [0.01, 0.11] | 0.992 | 0 | [-0.05, 0.04] | 0.557 | -0.19 | [-0.24, -0.14] | 1 | -0.1 | [-0.15, -0.04] | 1 |
| Sex | 0.25 | [0.2, 0.31] | 1 | -1.11 | [-1.16, -1.07] | 1 | -0.15 | [-0.21, -0.1] | 1 | 0.29 | [0.23, 0.35] | 1 | -0.27 | [-0.33, -0.22] | 1 | -0.53 | [-0.59, -0.48] | 1 | -0.21 | [-0.27, -0.15] | 1 | -0.28 | [-0.34, -0.22] | 1 |
| Smoking (y/n) | -0.15 | [-0.2, -0.1] | 1 | -0.27 | [-0.31, -0.22] | 1 | -0.22 | [-0.28, -0.17] | 1 | -0.21 | [-0.27, -0.15] | 1 | -0.2 | [-0.26, -0.15] | 1 | -0.25 | [-0.31, -0.2] | 1 | -0.12 | [-0.18, -0.06] | 1 | -0.18 | [-0.24, -0.12] | 1 |
| Urbanicity score (z) | 0.41 | [0.38, 0.43] | 1 | 0.25 | [0.23, 0.28] | 1 | 0.32 | [0.3, 0.35] | 1 | 0.04 | [0.01, 0.07] | 0.998 | 0.37 | [0.35, 0.4] | 1 | 0.27 | [0.24, 0.29] | 1 | 0.11 | [0.09, 0.14] | 1 | 0.05 | [0.02, 0.08] | 1 |
| Inactivity (z) | 0.12 | [0.1, 0.15] | 1 | 0.13 | [0.11, 0.15] | 1 | 0.13 | [0.11, 0.16] | 1 | 0.07 | [0.04, 0.1] | 1 | 0.12 | [0.09, 0.14] | 1 | 0.13 | [0.1, 0.15] | 1 | 0 | [-0.03, 0.03] | 0.517 | 0 | [-0.03, 0.03] | 0.54 |

| | HDL | | | Triglycerides | | | Systolic BP | | | Diastolic BP | | | Obese (y/n) | | | Hypertension (y/n) | | | Pre-diabetes (y/n) | | | Diabetes (y/n) | | |
|---|---|---|---|---|---|---|---|---|---|---|---|---|---|---|---|---|---|---|---|---|---|---|---|---|
| Predictor | Estimate | 95% CrI | Pr(direction) | Estimate | 95% CrI | Pr(direction) | Estimate | 95% CrI | Pr(direction) | Estimate | 95% CrI | Pr(direction) | Estimate | 95% CrI | Pr(direction) | Estimate | 95% CrI | Pr(direction) | Estimate | 95% CrI | Pr(direction) | Estimate | 95% CrI | Pr(direction) |
| Intercept | 0.2 | [0.17, 0.24] | 1 | -0.22 | [-0.25, -0.18] | 1 | -0.04 | [-0.07, -0.01] | 0.995 | 0.04 | [0, 0.07] | 0.983 | -1.29 | [-1.39, -1.19] | 1 | 0.16 | [0.08, 0.23] | 1 | -2.46 | [-2.59, -2.32] | 1 | -3.61 | [-3.86, -3.39] | 1 |
| Age (z) | 0.15 | [0.12, 0.19] | 1 | 0.1 | [0.07, 0.13] | 1 | 0.54 | [0.51, 0.57] | 1 | 0.3 | [0.27, 0.34] | 1 | -0.39 | [-0.49, -0.29] | 1 | 0.71 | [0.62, 0.79] | 1 | 0.52 | [0.4, 0.65] | 1 | 0.6 | [0.4, 0.79] | 1 |
| Age (z):Sex | 0.05 | [0, 0.1] | 0.979 | -0.15 | [-0.2, -0.1] | 1 | -0.31 | [-0.35, -0.26] | 1 | -0.15 | [-0.2, -0.1] | 1 | 0.12 | [-0.05, 0.3] | 0.918 | -0.26 | [-0.39, -0.14] | 1 | -0.09 | [-0.29, 0.11] | 0.808 | -0.36 | [-0.67, -0.05] | 0.99 |
| Sex | -0.39 | [-0.45, -0.33] | 1 | 0.4 | [0.34, 0.46] | 1 | 0.52 | [0.46, 0.57] | 1 | 0.15 | [0.09, 0.21] | 1 | -0.87 | [-1.07, -0.68] | 1 | 0.49 | [0.36, 0.62] | 1 | -0.15 | [-0.41, 0.11] | 0.872 | 0.06 | [-0.35, 0.45] | 0.624 |
| Smoking (y/n) | -0.2 | [-0.26, -0.14] | 1 | 0.2 | [0.14, 0.25] | 1 | -0.34 | [-0.39, -0.29] | 1 | -0.25 | [-0.31, -0.19] | 1 | -0.58 | [-0.77, -0.39] | 1 | -0.65 | [-0.78, -0.52] | 1 | -0.35 | [-0.58, -0.12] | 0.999 | -0.31 | [-0.65, 0.03] | 0.96 |
| Urbanicity score (z) | 0.08 | [0.05, 0.1] | 1 | 0.2 | [0.17, 0.23] | 1 | -0.07 | [-0.09, -0.04] | 1 | -0.03 | [-0.06, 0] | 0.983 | 0.89 | [0.81, 0.97] | 1 | -0.1 | [-0.16, -0.04] | 1 | 0.36 | [0.25, 0.47] | 1 | 0.38 | [0.21, 0.56] | 1 |
| Inactivity (z) | -0.05 | [-0.08, -0.02] | 1 | 0.1 | [0.07, 0.12] | 1 | 0.05 | [0.02, 0.07] | 1 | 0.09 | [0.06, 0.12] | 1 | 0.46 | [0.37, 0.54] | 1 | 0.18 | [0.12, 0.24] | 1 | 0.05 | [-0.06, 0.15] | 0.807 | 0.15 | [-0.02, 0.31] | 0.957 |

*Pr(direction) describes the proportion of the posterior that falls on the same side of 0 as the main association (i.e., a value of 0.96 can be interpreted as expressing 96% confidence that a given parameter is directionally greater or less than zero).

### Physical activity predictor: Daily steps; adjusted for urbanicity

| | Weight | | | Body fat | | | Waist circumference | | | Waist:hip ratio | | | BMI | | | BRI | | | Total cholesterol | | | LDL | | |
|---|---|---|---|---|---|---|---|---|---|---|---|---|---|---|---|---|---|---|---|---|---|---|---|---|
| Predictor | Estimate | 95% CrI | Pr(direction) | Estimate | 95% CrI | Pr(direction) | Estimate | 95% CrI | Pr(direction) | Estimate | 95% CrI | Pr(direction) | Estimate | 95% CrI | Pr(direction) | Estimate | 95% CrI | Pr(direction) | Estimate | 95% CrI | Pr(direction) | Estimate | 95% CrI | Pr(direction) |
| Intercept | -0.05 | [-0.08, -0.02] | 0.999 | 0.47 | [0.45, 0.5] | 1 | 0.11 | [0.07, 0.14] | 1 | -0.05 | [-0.08, -0.02] | 0.998 | 0.15 | [0.12, 0.19] | 1 | 0.26 | [0.23, 0.29] | 1 | 0.12 | [0.08, 0.15] | 1 | 0.15 | [0.12, 0.19] | 1 |
| Age (z) | -0.25 | [-0.28, -0.22] | 1 | -0.06 | [-0.09, -0.04] | 1 | 0.03 | [0, 0.06] | 0.96 | 0.23 | [0.2, 0.27] | 1 | -0.17 | [-0.2, -0.14] | 1 | 0.12 | [0.09, 0.16] | 1 | 0.22 | [0.19, 0.25] | 1 | 0.17 | [0.13, 0.21] | 1 |
| Age (z):Sex | 0.01 | [-0.04, 0.06] | 0.648 | 0.1 | [0.06, 0.14] | 1 | 0.01 | [-0.04, 0.06] | 0.633 | -0.03 | [-0.08, 0.03] | 0.843 | 0.06 | [0.01, 0.11] | 0.994 | -0.01 | [-0.05, 0.04] | 0.595 | -0.2 | [-0.25, -0.15] | 1 | -0.11 | [-0.17, -0.06] | 1 |
| Sex | 0.29 | [0.24, 0.35] | 1 | -1.07 | [-1.11, -1.02] | 1 | -0.11 | [-0.17, -0.06] | 1 | 0.31 | [0.25, 0.37] | 1 | -0.23 | [-0.29, -0.18] | 1 | -0.49 | [-0.54, -0.44] | 1 | -0.2 | [-0.26, -0.14] | 1 | -0.26 | [-0.32, -0.2] | 1 |
| Smoking (y/n) | -0.15 | [-0.2, -0.1] | 1 | -0.26 | [-0.31, -0.22] | 1 | -0.22 | [-0.28, -0.17] | 1 | -0.21 | [-0.27, -0.15] | 1 | -0.2 | [-0.25, -0.15] | 1 | -0.25 | [-0.3, -0.2] | 1 | -0.11 | [-0.17, -0.05] | 1 | -0.17 | [-0.23, -0.1] | 1 |
| Urbanicity score (z) | 0.42 | [0.39, 0.44] | 1 | 0.26 | [0.24, 0.28] | 1 | 0.33 | [0.31, 0.36] | 1 | 0.04 | [0.02, 0.07] | 1 | 0.38 | [0.36, 0.4] | 1 | 0.28 | [0.25, 0.3] | 1 | 0.1 | [0.07, 0.13] | 1 | 0.03 | [0.01, 0.06] | 0.992 |
| Daily steps (z) | -0.11 | [-0.13, -0.09] | 1 | -0.11 | [-0.13, -0.09] | 1 | -0.11 | [-0.13, -0.08] | 1 | -0.05 | [-0.08, -0.03] | 1 | -0.1 | [-0.12, -0.07] | 1 | -0.1 | [-0.12, -0.07] | 1 | -0.06 | [-0.09, -0.03] | 1 | -0.07 | [-0.1, -0.04] | 1 |

| | HDL | | | Triglycerides | | | Systolic BP | | | Diastolic BP | | | Obese (y/n) | | | Hypertension (y/n) | | | Pre-diabetes (y/n) | | | Diabetes (y/n) | | |
|---|---|---|---|---|---|---|---|---|---|---|---|---|---|---|---|---|---|---|---|---|---|---|---|---|
| Predictor | Estimate | 95% CrI | Pr(direction) | Estimate | 95% CrI | Pr(direction) | Estimate | 95% CrI | Pr(direction) | Estimate | 95% CrI | Pr(direction) | Estimate | 95% CrI | Pr(direction) | Estimate | 95% CrI | Pr(direction) | Estimate | 95% CrI | Pr(direction) | Estimate | 95% CrI | Pr(direction) |
| Intercept | 0.21 | [0.18, 0.24] | 1 | -0.22 | [-0.26, -0.19] | 1 | -0.04 | [-0.07, -0.01] | 0.995 | 0.03 | [-0.01, 0.06] | 0.948 | -1.33 | [-1.43, -1.23] | 1 | 0.15 | [0.07, 0.22] | 1 | -2.45 | [-2.59, -2.31] | 1 | -3.65 | [-3.88, -3.42] | 1 |
| Age (z) | 0.15 | [0.12, 0.18] | 1 | 0.1 | [0.06, 0.13] | 1 | 0.54 | [0.51, 0.57] | 1 | 0.29 | [0.26, 0.33] | 1 | -0.41 | [-0.5, -0.31] | 1 | 0.71 | [0.63, 0.79] | 1 | 0.48 | [0.35, 0.6] | 1 | 0.56 | [0.37, 0.75] | 1 |
| Age (z):Sex | 0.05 | [0, 0.1] | 0.973 | -0.16 | [-0.21, -0.11] | 1 | -0.31 | [-0.36, -0.26] | 1 | -0.14 | [-0.19, -0.09] | 1 | 0.1 | [-0.07, 0.28] | 0.882 | -0.28 | [-0.4, -0.16] | 1 | -0.06 | [-0.25, 0.14] | 0.715 | -0.34 | [-0.64, -0.04] | 0.988 |
| Sex | -0.41 | [-0.47, -0.35] | 1 | 0.42 | [0.37, 0.48] | 1 | 0.53 | [0.48, 0.58] | 1 | 0.19 | [0.13, 0.25] | 1 | -0.72 | [-0.92, -0.52] | 1 | 0.58 | [0.45, 0.71] | 1 | -0.17 | [-0.43, 0.09] | 0.9 | 0.11 | [-0.29, 0.49] | 0.711 |
| Smoking (y/n) | -0.2 | [-0.26, -0.14] | 1 | 0.19 | [0.14, 0.25] | 1 | -0.35 | [-0.4, -0.3] | 1 | -0.26 | [-0.32, -0.2] | 1 | -0.59 | [-0.77, -0.4] | 1 | -0.67 | [-0.8, -0.54] | 1 | -0.32 | [-0.55, -0.1] | 0.997 | -0.28 | [-0.64, 0.07] | 0.937 |
| Urbanicity score (z) | 0.07 | [0.04, 0.1] | 1 | 0.2 | [0.17, 0.23] | 1 | -0.06 | [-0.09, -0.04] | 1 | -0.02 | [-0.05, 0.01] | 0.914 | 0.91 | [0.83, 0.99] | 1 | -0.08 | [-0.14, -0.03] | 0.997 | 0.36 | [0.26, 0.47] | 1 | 0.37 | [0.2, 0.55] | 1 |
| Daily steps (z) | 0.03 | [0, 0.06] | 0.99 | -0.08 | [-0.11, -0.06] | 1 | -0.04 | [-0.07, -0.02] | 0.999 | -0.07 | [-0.1, -0.05] | 1 | -0.43 | [-0.52, -0.35] | 1 | -0.19 | [-0.25, -0.13] | 1 | -0.06 | [-0.17, 0.04] | 0.87 | -0.13 | [-0.3, 0.04] | 0.936 |

*Pr(direction) describes the proportion of the posterior that falls on the same side of 0 as the main association (i.e., a value of 0.96 can be interpreted as expressing 96% confidence that a given parameter is directionally greater or less than zero).

### Physical activity predictor: Intensity gradient; adjusted for urbanicity

| | Weight | | | Body fat | | | Waist circumference | | | Waist:hip ratio | | | BMI | | | BRI | | | Total cholesterol | | | LDL | | |
|---|---|---|---|---|---|---|---|---|---|---|---|---|---|---|---|---|---|---|---|---|---|---|---|---|
| Predictor | Estimate | 95% CrI | Pr(direction) | Estimate | 95% CrI | Pr(direction) | Estimate | 95% CrI | Pr(direction) | Estimate | 95% CrI | Pr(direction) | Estimate | 95% CrI | Pr(direction) | Estimate | 95% CrI | Pr(direction) | Estimate | 95% CrI | Pr(direction) | Estimate | 95% CrI | Pr(direction) |
| Intercept | -0.07 | [-0.1, -0.04] | 1 | 0.45 | [0.42, 0.48] | 1 | 0.08 | [0.05, 0.12] | 1 | -0.05 | [-0.08, -0.01] | 0.998 | 0.14 | [0.11, 0.17] | 1 | 0.24 | [0.2, 0.27] | 1 | 0.12 | [0.08, 0.15] | 1 | 0.14 | [0.11, 0.18] | 1 |
| Age (z) | -0.26 | [-0.29, -0.22] | 1 | -0.08 | [-0.11, -0.05] | 1 | 0.01 | [-0.02, 0.05] | 0.789 | 0.24 | [0.21, 0.28] | 1 | -0.18 | [-0.21, -0.15] | 1 | 0.11 | [0.07, 0.14] | 1 | 0.23 | [0.19, 0.26] | 1 | 0.16 | [0.13, 0.2] | 1 |
| Age (z):Sex | 0.01 | [-0.03, 0.06] | 0.706 | 0.1 | [0.06, 0.14] | 1 | 0.01 | [-0.04, 0.06] | 0.632 | -0.01 | [-0.07, 0.04] | 0.707 | 0.07 | [0.02, 0.11] | 0.996 | -0.01 | [-0.05, 0.04] | 0.601 | -0.19 | [-0.24, -0.13] | 1 | -0.1 | [-0.16, -0.05] | 1 |
| Sex | 0.32 | [0.26, 0.38] | 1 | -1.02 | [-1.07, -0.97] | 1 | -0.07 | [-0.13, -0.01] | 0.987 | 0.3 | [0.23, 0.36] | 1 | -0.21 | [-0.27, -0.16] | 1 | -0.45 | [-0.51, -0.39] | 1 | -0.22 | [-0.28, -0.15] | 1 | -0.25 | [-0.32, -0.19] | 1 |
| Smoking (y/n) | -0.13 | [-0.19, -0.08] | 1 | -0.25 | [-0.29, -0.2] | 1 | -0.2 | [-0.26, -0.15] | 1 | -0.2 | [-0.26, -0.15] | 1 | -0.19 | [-0.24, -0.13] | 1 | -0.23 | [-0.29, -0.18] | 1 | -0.11 | [-0.16, -0.05] | 1 | -0.16 | [-0.22, -0.1] | 1 |
| Urbanicity score (z) | 0.42 | [0.4, 0.45] | 1 | 0.26 | [0.24, 0.28] | 1 | 0.33 | [0.31, 0.36] | 1 | 0.05 | [0.02, 0.08] | 1 | 0.39 | [0.36, 0.41] | 1 | 0.28 | [0.25, 0.3] | 1 | 0.11 | [0.08, 0.13] | 1 | 0.04 | [0.01, 0.06] | 0.994 |
| Intensity gradient (z) | -0.07 | [-0.1, -0.04] | 1 | -0.09 | [-0.12, -0.07] | 1 | -0.09 | [-0.12, -0.06] | 1 | 0.01 | [-0.03, 0.04] | 0.62 | -0.06 | [-0.09, -0.03] | 1 | -0.09 | [-0.12, -0.06] | 1 | 0 | [-0.03, 0.03] | 0.518 | -0.04 | [-0.07, 0] | 0.987 |

| | HDL | | | Triglycerides | | | Systolic BP | | | Diastolic BP | | | Obese (y/n) | | | Hypertension (y/n) | | | Pre-diabetes (y/n) | | | Diabetes (y/n) | | |
|---|---|---|---|---|---|---|---|---|---|---|---|---|---|---|---|---|---|---|---|---|---|---|---|---|
| Predictor | Estimate | 95% CrI | Pr(direction) | Estimate | 95% CrI | Pr(direction) | Estimate | 95% CrI | Pr(direction) | Estimate | 95% CrI | Pr(direction) | Estimate | 95% CrI | Pr(direction) | Estimate | 95% CrI | Pr(direction) | Estimate | 95% CrI | Pr(direction) | Estimate | 95% CrI | Pr(direction) |
| Intercept | 0.23 | [0.19, 0.26] | 1 | -0.23 | [-0.27, -0.2] | 1 | -0.05 | [-0.08, -0.02] | 0.999 | 0.01 | [-0.03, 0.04] | 0.614 | -1.4 | [-1.5, -1.3] | 1 | 0.11 | [0.03, 0.19] | 0.997 | -2.5 | [-2.65, -2.36] | 1 | -3.73 | [-3.98, -3.5] | 1 |
| Age (z) | 0.17 | [0.13, 0.2] | 1 | 0.09 | [0.06, 0.13] | 1 | 0.54 | [0.5, 0.57] | 1 | 0.28 | [0.24, 0.31] | 1 | -0.47 | [-0.57, -0.37] | 1 | 0.69 | [0.6, 0.77] | 1 | 0.42 | [0.3, 0.55] | 1 | 0.48 | [0.28, 0.69] | 1 |
| Age (z):Sex | 0.06 | [0.01, 0.11] | 0.987 | -0.15 | [-0.2, -0.1] | 1 | -0.31 | [-0.36, -0.26] | 1 | -0.14 | [-0.2, -0.09] | 1 | 0.12 | [-0.06, 0.3] | 0.898 | -0.28 | [-0.4, -0.16] | 1 | -0.09 | [-0.29, 0.1] | 0.823 | -0.38 | [-0.68, -0.08] | 0.993 |
| Sex | -0.45 | [-0.51, -0.38] | 1 | 0.43 | [0.37, 0.5] | 1 | 0.55 | [0.49, 0.6] | 1 | 0.23 | [0.17, 0.3] | 1 | -0.54 | [-0.75, -0.34] | 1 | 0.63 | [0.49, 0.77] | 1 | -0.04 | [-0.32, 0.23] | 0.608 | 0.28 | [-0.14, 0.7] | 0.905 |
| Smoking (y/n) | -0.21 | [-0.26, -0.15] | 1 | 0.21 | [0.15, 0.27] | 1 | -0.34 | [-0.39, -0.29] | 1 | -0.24 | [-0.3, -0.18] | 1 | -0.55 | [-0.73, -0.36] | 1 | -0.63 | [-0.77, -0.5] | 1 | -0.32 | [-0.55, -0.08] | 0.997 | -0.26 | [-0.63, 0.09] | 0.929 |
| Urbanicity score (z) | 0.07 | [0.05, 0.1] | 1 | 0.21 | [0.18, 0.23] | 1 | -0.06 | [-0.09, -0.04] | 1 | -0.02 | [-0.05, 0.01] | 0.917 | 0.92 | [0.84, 1] | 1 | -0.08 | [-0.13, -0.02] | 0.994 | 0.35 | [0.24, 0.45] | 1 | 0.35 | [0.18, 0.53] | 1 |
| Intensity gradient (z) | 0.06 | [0.03, 0.1] | 1 | -0.04 | [-0.07, -0.01] | 0.996 | -0.03 | [-0.06, 0] | 0.984 | -0.09 | [-0.12, -0.05] | 1 | -0.35 | [-0.45, -0.25] | 1 | -0.14 | [-0.21, -0.06] | 1 | -0.2 | [-0.32, -0.08] | 0.999 | -0.3 | [-0.49, -0.12] | 0.999 |

*Pr(direction) describes the proportion of the posterior that falls on the same side of 0 as the main association (i.e., a value of 0.96 can be interpreted as expressing 96% confidence that a given parameter is directionally greater or less than zero).

### Physical activity predictor: Light activity; adjusted for urbanicity

| | Weight | | | Body fat | | | Waist circumference | | | Waist:hip ratio | | | BMI | | | BRI | | | Total cholesterol | | | LDL | | |
|---|---|---|---|---|---|---|---|---|---|---|---|---|---|---|---|---|---|---|---|---|---|---|---|---|
| Predictor | Estimate | 95% CrI | Pr(direction) | Estimate | 95% CrI | Pr(direction) | Estimate | 95% CrI | Pr(direction) | Estimate | 95% CrI | Pr(direction) | Estimate | 95% CrI | Pr(direction) | Estimate | 95% CrI | Pr(direction) | Estimate | 95% CrI | Pr(direction) | Estimate | 95% CrI | Pr(direction) |
| Intercept | -0.04 | [-0.07, 0] | 0.981 | 0.49 | [0.46, 0.52] | 1 | 0.12 | [0.09, 0.16] | 1 | -0.05 | [-0.08, -0.01] | 0.998 | 0.17 | [0.14, 0.2] | 1 | 0.27 | [0.24, 0.3] | 1 | 0.15 | [0.11, 0.18] | 1 | 0.18 | [0.14, 0.22] | 1 |
| Age (z) | -0.2 | [-0.23, -0.17] | 1 | -0.02 | [-0.05, 0] | 0.95 | 0.07 | [0.03, 0.1] | 0.999 | 0.25 | [0.21, 0.28] | 1 | -0.13 | [-0.17, -0.1] | 1 | 0.15 | [0.12, 0.18] | 1 | 0.26 | [0.22, 0.3] | 1 | 0.2 | [0.16, 0.23] | 1 |
| Age (z):Sex | 0.02 | [-0.03, 0.06] | 0.744 | 0.11 | [0.07, 0.15] | 1 | 0.02 | [-0.03, 0.07] | 0.824 | -0.01 | [-0.07, 0.04] | 0.713 | 0.07 | [0.02, 0.12] | 0.998 | 0.01 | [-0.04, 0.06] | 0.666 | -0.2 | [-0.26, -0.15] | 1 | -0.1 | [-0.16, -0.04] | 1 |
| Sex | 0.24 | [0.18, 0.29] | 1 | -1.13 | [-1.17, -1.08] | 1 | -0.16 | [-0.22, -0.1] | 1 | 0.3 | [0.24, 0.36] | 1 | -0.28 | [-0.33, -0.23] | 1 | -0.53 | [-0.59, -0.48] | 1 | -0.25 | [-0.31, -0.19] | 1 | -0.3 | [-0.36, -0.24] | 1 |
| Smoking (y/n) | -0.15 | [-0.2, -0.1] | 1 | -0.27 | [-0.31, -0.22] | 1 | -0.22 | [-0.28, -0.16] | 1 | -0.21 | [-0.27, -0.15] | 1 | -0.2 | [-0.25, -0.15] | 1 | -0.25 | [-0.3, -0.19] | 1 | -0.13 | [-0.19, -0.07] | 1 | -0.18 | [-0.25, -0.12] | 1 |
| Urbanicity score (z) | 0.43 | [0.4, 0.45] | 1 | 0.27 | [0.25, 0.3] | 1 | 0.34 | [0.32, 0.37] | 1 | 0.05 | [0.03, 0.08] | 1 | 0.39 | [0.37, 0.41] | 1 | 0.29 | [0.26, 0.31] | 1 | 0.12 | [0.09, 0.14] | 1 | 0.05 | [0.02, 0.08] | 1 |
| Light activity (z) | -0.09 | [-0.11, -0.06] | 1 | -0.1 | [-0.12, -0.08] | 1 | -0.08 | [-0.1, -0.05] | 1 | -0.01 | [-0.04, 0.01] | 0.825 | -0.08 | [-0.1, -0.05] | 1 | -0.07 | [-0.09, -0.04] | 1 | -0.08 | [-0.11, -0.05] | 1 | -0.04 | [-0.07, -0.01] | 0.994 |

| | HDL | | | Triglycerides | | | Systolic BP | | | Diastolic BP | | | Obese (y/n) | | | Hypertension (y/n) | | | Pre-diabetes (y/n) | | | Diabetes (y/n) | | |
|---|---|---|---|---|---|---|---|---|---|---|---|---|---|---|---|---|---|---|---|---|---|---|---|---|
| Predictor | Estimate | 95% CrI | Pr(direction) | Estimate | 95% CrI | Pr(direction) | Estimate | 95% CrI | Pr(direction) | Estimate | 95% CrI | Pr(direction) | Estimate | 95% CrI | Pr(direction) | Estimate | 95% CrI | Pr(direction) | Estimate | 95% CrI | Pr(direction) | Estimate | 95% CrI | Pr(direction) |
| Intercept | 0.22 | [0.18, 0.25] | 1 | -0.21 | [-0.24, -0.17] | 1 | -0.04 | [-0.07, -0.01] | 0.994 | 0.03 | [-0.01, 0.07] | 0.955 | -1.27 | [-1.37, -1.17] | 1 | 0.15 | [0.07, 0.22] | 1 | -2.48 | [-2.63, -2.34] | 1 | -3.67 | [-3.92, -3.43] | 1 |
| Age (z) | 0.16 | [0.13, 0.2] | 1 | 0.13 | [0.1, 0.17] | 1 | 0.55 | [0.52, 0.59] | 1 | 0.32 | [0.28, 0.35] | 1 | -0.29 | [-0.39, -0.19] | 1 | 0.74 | [0.65, 0.82] | 1 | 0.51 | [0.38, 0.64] | 1 | 0.58 | [0.39, 0.78] | 1 |
| Age (z):Sex | 0.04 | [-0.01, 0.09] | 0.917 | -0.15 | [-0.2, -0.1] | 1 | -0.3 | [-0.35, -0.26] | 1 | -0.14 | [-0.19, -0.08] | 1 | 0.15 | [-0.03, 0.32] | 0.947 | -0.24 | [-0.37, -0.12] | 1 | -0.07 | [-0.27, 0.13] | 0.747 | -0.32 | [-0.63, -0.01] | 0.979 |
| Sex | -0.42 | [-0.48, -0.36] | 1 | 0.38 | [0.32, 0.44] | 1 | 0.51 | [0.46, 0.57] | 1 | 0.16 | [0.09, 0.22] | 1 | -0.86 | [-1.05, -0.67] | 1 | 0.48 | [0.36, 0.62] | 1 | -0.11 | [-0.38, 0.15] | 0.795 | 0.16 | [-0.25, 0.57] | 0.778 |
| Smoking (y/n) | -0.21 | [-0.27, -0.15] | 1 | 0.19 | [0.14, 0.25] | 1 | -0.34 | [-0.39, -0.29] | 1 | -0.25 | [-0.31, -0.19] | 1 | -0.56 | [-0.74, -0.37] | 1 | -0.64 | [-0.77, -0.51] | 1 | -0.33 | [-0.56, -0.11] | 0.998 | -0.27 | [-0.62, 0.09] | 0.926 |
| Urbanicity score (z) | 0.07 | [0.04, 0.1] | 1 | 0.21 | [0.19, 0.24] | 1 | -0.06 | [-0.09, -0.04] | 1 | -0.02 | [-0.04, 0.01] | 0.867 | 0.96 | [0.88, 1.04] | 1 | -0.08 | [-0.13, -0.02] | 0.995 | 0.37 | [0.27, 0.47] | 1 | 0.4 | [0.23, 0.57] | 1 |
| Light activity (z) | -0.04 | [-0.07, -0.01] | 0.996 | -0.09 | [-0.12, -0.06] | 1 | -0.03 | [-0.06, 0] | 0.991 | -0.02 | [-0.05, 0] | 0.944 | -0.23 | [-0.32, -0.15] | 1 | -0.07 | [-0.14, -0.01] | 0.986 | 0.07 | [-0.04, 0.17] | 0.892 | 0.15 | [-0.01, 0.31] | 0.963 |

*Pr(direction) describes the proportion of the posterior that falls on the same side of 0 as the main association (i.e., a value of 0.96 can be interpreted as expressing 96% confidence that a given parameter is directionally greater or less than zero).

### Physical activity predictor: Moderate activity; adjusted for urbanicity

| | Weight | | | Body fat | | | Waist circumference | | | Waist:hip ratio | | | BMI | | | BRI | | | Total cholesterol | | | LDL | | |
|---|---|---|---|---|---|---|---|---|---|---|---|---|---|---|---|---|---|---|---|---|---|---|---|---|
| Predictor | Estimate | 95% CrI | Pr(direction) | Estimate | 95% CrI | Pr(direction) | Estimate | 95% CrI | Pr(direction) | Estimate | 95% CrI | Pr(direction) | Estimate | 95% CrI | Pr(direction) | Estimate | 95% CrI | Pr(direction) | Estimate | 95% CrI | Pr(direction) | Estimate | 95% CrI | Pr(direction) |
| Intercept | -0.05 | [-0.09, -0.02] | 1 | 0.47 | [0.45, 0.5] | 1 | 0.11 | [0.07, 0.14] | 1 | -0.05 | [-0.09, -0.01] | 0.997 | 0.15 | [0.12, 0.18] | 1 | 0.26 | [0.23, 0.29] | 1 | 0.13 | [0.09, 0.16] | 1 | 0.17 | [0.14, 0.21] | 1 |
| Age (z) | -0.24 | [-0.28, -0.21] | 1 | -0.07 | [-0.1, -0.05] | 1 | 0.03 | [-0.01, 0.06] | 0.93 | 0.23 | [0.2, 0.26] | 1 | -0.17 | [-0.2, -0.14] | 1 | 0.12 | [0.08, 0.15] | 1 | 0.23 | [0.19, 0.26] | 1 | 0.17 | [0.14, 0.21] | 1 |
| Age (z):Sex | 0.02 | [-0.03, 0.07] | 0.763 | 0.12 | [0.07, 0.16] | 1 | 0.02 | [-0.03, 0.07] | 0.822 | -0.02 | [-0.07, 0.03] | 0.776 | 0.07 | [0.02, 0.12] | 0.999 | 0.01 | [-0.04, 0.06] | 0.636 | -0.19 | [-0.25, -0.14] | 1 | -0.11 | [-0.16, -0.05] | 1 |
| Sex | 0.29 | [0.24, 0.35] | 1 | -1.07 | [-1.11, -1.02] | 1 | -0.11 | [-0.17, -0.06] | 1 | 0.31 | [0.25, 0.37] | 1 | -0.24 | [-0.29, -0.18] | 1 | -0.49 | [-0.54, -0.44] | 1 | -0.2 | [-0.26, -0.15] | 1 | -0.27 | [-0.33, -0.2] | 1 |
| Smoking (y/n) | -0.15 | [-0.2, -0.1] | 1 | -0.27 | [-0.31, -0.22] | 1 | -0.22 | [-0.28, -0.17] | 1 | -0.21 | [-0.27, -0.15] | 1 | -0.2 | [-0.25, -0.14] | 1 | -0.25 | [-0.3, -0.2] | 1 | -0.12 | [-0.18, -0.07] | 1 | -0.19 | [-0.25, -0.13] | 1 |
| Urbanicity score (z) | 0.42 | [0.4, 0.45] | 1 | 0.27 | [0.25, 0.29] | 1 | 0.34 | [0.31, 0.36] | 1 | 0.05 | [0.02, 0.08] | 1 | 0.39 | [0.36, 0.41] | 1 | 0.28 | [0.26, 0.31] | 1 | 0.11 | [0.09, 0.14] | 1 | 0.05 | [0.02, 0.08] | 1 |
| Moderate activity (z) | -0.07 | [-0.09, -0.04] | 1 | -0.09 | [-0.11, -0.07] | 1 | -0.07 | [-0.1, -0.05] | 1 | -0.05 | [-0.08, -0.02] | 1 | -0.06 | [-0.08, -0.03] | 1 | -0.07 | [-0.1, -0.04] | 1 | -0.03 | [-0.06, 0] | 0.977 | -0.05 | [-0.08, -0.02] | 1 |

| | HDL | | | Triglycerides | | | Systolic BP | | | Diastolic BP | | | Obese (y/n) | | | Hypertension (y/n) | | | Pre-diabetes (y/n) | | | Diabetes (y/n) | | |
|---|---|---|---|---|---|---|---|---|---|---|---|---|---|---|---|---|---|---|---|---|---|---|---|---|
| Predictor | Estimate | 95% CrI | Pr(direction) | Estimate | 95% CrI | Pr(direction) | Estimate | 95% CrI | Pr(direction) | Estimate | 95% CrI | Pr(direction) | Estimate | 95% CrI | Pr(direction) | Estimate | 95% CrI | Pr(direction) | Estimate | 95% CrI | Pr(direction) | Estimate | 95% CrI | Pr(direction) |
| Intercept | 0.21 | [0.17, 0.24] | 1 | -0.23 | [-0.26, -0.19] | 1 | -0.05 | [-0.08, -0.01] | 0.998 | 0.03 | [-0.01, 0.06] | 0.943 | -1.32 | [-1.42, -1.22] | 1 | 0.14 | [0.07, 0.22] | 1 | -2.46 | [-2.6, -2.32] | 1 | -3.63 | [-3.86, -3.4] | 1 |
| Age (z) | 0.16 | [0.13, 0.2] | 1 | 0.09 | [0.06, 0.13] | 1 | 0.53 | [0.5, 0.56] | 1 | 0.29 | [0.25, 0.32] | 1 | -0.44 | [-0.54, -0.33] | 1 | 0.67 | [0.59, 0.76] | 1 | 0.5 | [0.37, 0.63] | 1 | 0.53 | [0.33, 0.74] | 1 |
| Age (z):Sex | 0.06 | [0, 0.11] | 0.983 | -0.15 | [-0.2, -0.1] | 1 | -0.31 | [-0.36, -0.26] | 1 | -0.15 | [-0.2, -0.09] | 1 | 0.13 | [-0.04, 0.31] | 0.93 | -0.26 | [-0.39, -0.14] | 1 | -0.1 | [-0.3, 0.1] | 0.832 | -0.37 | [-0.68, -0.08] | 0.992 |
| Sex | -0.41 | [-0.47, -0.35] | 1 | 0.43 | [0.37, 0.49] | 1 | 0.54 | [0.48, 0.59] | 1 | 0.18 | [0.12, 0.24] | 1 | -0.7 | [-0.89, -0.51] | 1 | 0.57 | [0.43, 0.7] | 1 | -0.12 | [-0.39, 0.13] | 0.818 | 0.14 | [-0.25, 0.53] | 0.753 |
| Smoking (y/n) | -0.2 | [-0.25, -0.14] | 1 | 0.2 | [0.14, 0.25] | 1 | -0.34 | [-0.4, -0.29] | 1 | -0.26 | [-0.32, -0.2] | 1 | -0.58 | [-0.77, -0.4] | 1 | -0.67 | [-0.8, -0.54] | 1 | -0.36 | [-0.59, -0.13] | 0.998 | -0.33 | [-0.69, 0.02] | 0.97 |
| Urbanicity score (z) | 0.07 | [0.05, 0.1] | 1 | 0.21 | [0.18, 0.24] | 1 | -0.06 | [-0.09, -0.04] | 1 | -0.02 | [-0.04, 0.01] | 0.924 | 0.94 | [0.85, 1.02] | 1 | -0.08 | [-0.14, -0.02] | 0.996 | 0.36 | [0.25, 0.47] | 1 | 0.39 | [0.22, 0.56] | 1 |
| Moderate activity (z) | 0.07 | [0.04, 0.09] | 1 | -0.06 | [-0.09, -0.03] | 1 | -0.05 | [-0.08, -0.03] | 1 | -0.08 | [-0.11, -0.06] | 1 | -0.34 | [-0.43, -0.26] | 1 | -0.21 | [-0.27, -0.14] | 1 | -0.08 | [-0.19, 0.02] | 0.938 | -0.23 | [-0.39, -0.07] | 0.999 |

*Pr(direction) describes the proportion of the posterior that falls on the same side of 0 as the main association (i.e., a value of 0.96 can be interpreted as expressing 96% confidence that a given parameter is directionally greater or less than zero).

### Physical activity predictor: Vigorous activity; adjusted for urbanicity

| | Weight | | | Body fat | | | Waist circumference | | | Waist:hip ratio | | | BMI | | | BRI | | | Total cholesterol | | | LDL | | |
|---|---|---|---|---|---|---|---|---|---|---|---|---|---|---|---|---|---|---|---|---|---|---|---|---|
| Predictor | Estimate | 95% CrI | Pr(direction) | Estimate | 95% CrI | Pr(direction) | Estimate | 95% CrI | Pr(direction) | Estimate | 95% CrI | Pr(direction) | Estimate | 95% CrI | Pr(direction) | Estimate | 95% CrI | Pr(direction) | Estimate | 95% CrI | Pr(direction) | Estimate | 95% CrI | Pr(direction) |
| Intercept | -0.08 | [-0.11, -0.05] | 1 | 0.45 | [0.42, 0.47] | 1 | 0.08 | [0.05, 0.11] | 1 | -0.06 | [-0.1, -0.03] | 1 | 0.13 | [0.1, 0.16] | 1 | 0.23 | [0.2, 0.27] | 1 | 0.12 | [0.09, 0.16] | 1 | 0.16 | [0.12, 0.2] | 1 |
| Age (z) | -0.25 | [-0.28, -0.22] | 1 | -0.08 | [-0.11, -0.05] | 1 | 0.02 | [-0.02, 0.05] | 0.86 | 0.23 | [0.2, 0.27] | 1 | -0.18 | [-0.21, -0.14] | 1 | 0.11 | [0.08, 0.14] | 1 | 0.23 | [0.19, 0.27] | 1 | 0.17 | [0.14, 0.21] | 1 |
| Age (z):Sex | 0.01 | [-0.04, 0.05] | 0.592 | 0.1 | [0.06, 0.14] | 1 | 0.01 | [-0.04, 0.06] | 0.644 | -0.02 | [-0.07, 0.03] | 0.792 | 0.06 | [0.01, 0.11] | 0.995 | 0 | [-0.05, 0.05] | 0.52 | -0.19 | [-0.25, -0.14] | 1 | -0.11 | [-0.16, -0.05] | 1 |
| Sex | 0.35 | [0.29, 0.41] | 1 | -1.01 | [-1.05, -0.96] | 1 | -0.05 | [-0.11, 0.01] | 0.937 | 0.33 | [0.27, 0.39] | 1 | -0.19 | [-0.24, -0.13] | 1 | -0.43 | [-0.49, -0.38] | 1 | -0.2 | [-0.26, -0.14] | 1 | -0.24 | [-0.31, -0.18] | 1 |
| Smoking (y/n) | -0.14 | [-0.19, -0.08] | 1 | -0.25 | [-0.3, -0.21] | 1 | -0.21 | [-0.26, -0.15] | 1 | -0.2 | [-0.26, -0.14] | 1 | -0.19 | [-0.24, -0.13] | 1 | -0.24 | [-0.29, -0.19] | 1 | -0.12 | [-0.18, -0.06] | 1 | -0.18 | [-0.24, -0.12] | 1 |
| Urbanicity score (z) | 0.41 | [0.39, 0.44] | 1 | 0.26 | [0.24, 0.28] | 1 | 0.33 | [0.3, 0.35] | 1 | 0.05 | [0.02, 0.07] | 0.999 | 0.38 | [0.35, 0.4] | 1 | 0.27 | [0.25, 0.3] | 1 | 0.11 | [0.08, 0.14] | 1 | 0.04 | [0.02, 0.07] | 1 |
| Vigorous activity (z) | -0.1 | [-0.13, -0.08] | 1 | -0.11 | [-0.13, -0.09] | 1 | -0.11 | [-0.14, -0.09] | 1 | -0.04 | [-0.07, -0.01] | 0.999 | -0.08 | [-0.11, -0.06] | 1 | -0.1 | [-0.13, -0.07] | 1 | -0.02 | [-0.05, 0.01] | 0.892 | -0.05 | [-0.08, -0.02] | 0.999 |

| | HDL | | | Triglycerides | | | Systolic BP | | | Diastolic BP | | | Obese (y/n) | | | Hypertension (y/n) | | | Pre-diabetes (y/n) | | | Diabetes (y/n) | | |
|---|---|---|---|---|---|---|---|---|---|---|---|---|---|---|---|---|---|---|---|---|---|---|---|---|
| Predictor | Estimate | 95% CrI | Pr(direction) | Estimate | 95% CrI | Pr(direction) | Estimate | 95% CrI | Pr(direction) | Estimate | 95% CrI | Pr(direction) | Estimate | 95% CrI | Pr(direction) | Estimate | 95% CrI | Pr(direction) | Estimate | 95% CrI | Pr(direction) | Estimate | 95% CrI | Pr(direction) |
| Intercept | 0.23 | [0.2, 0.27] | 1 | -0.24 | [-0.28, -0.21] | 1 | -0.05 | [-0.08, -0.02] | 1 | 0.01 | [-0.02, 0.05] | 0.75 | -1.41 | [-1.51, -1.31] | 1 | 0.1 | [0.03, 0.18] | 0.996 | -2.51 | [-2.66, -2.37] | 1 | -3.65 | [-3.9, -3.41] | 1 |
| Age (z) | 0.17 | [0.14, 0.21] | 1 | 0.09 | [0.05, 0.12] | 1 | 0.54 | [0.51, 0.57] | 1 | 0.29 | [0.26, 0.33] | 1 | -0.44 | [-0.55, -0.34] | 1 | 0.68 | [0.6, 0.77] | 1 | 0.48 | [0.35, 0.6] | 1 | 0.59 | [0.39, 0.78] | 1 |
| Age (z):Sex | 0.07 | [0.01, 0.12] | 0.993 | -0.15 | [-0.2, -0.1] | 1 | -0.3 | [-0.35, -0.25] | 1 | -0.15 | [-0.2, -0.1] | 1 | 0.12 | [-0.05, 0.3] | 0.911 | -0.27 | [-0.39, -0.15] | 1 | -0.13 | [-0.32, 0.07] | 0.897 | -0.36 | [-0.68, -0.06] | 0.991 |
| Sex | -0.47 | [-0.52, -0.41] | 1 | 0.47 | [0.41, 0.53] | 1 | 0.53 | [0.48, 0.59] | 1 | 0.21 | [0.15, 0.28] | 1 | -0.52 | [-0.73, -0.32] | 1 | 0.63 | [0.5, 0.78] | 1 | -0.01 | [-0.28, 0.25] | 0.523 | 0.16 | [-0.26, 0.56] | 0.767 |
| Smoking (y/n) | -0.21 | [-0.27, -0.15] | 1 | 0.21 | [0.15, 0.26] | 1 | -0.34 | [-0.39, -0.28] | 1 | -0.24 | [-0.3, -0.19] | 1 | -0.54 | [-0.73, -0.36] | 1 | -0.64 | [-0.77, -0.51] | 1 | -0.34 | [-0.57, -0.11] | 0.998 | -0.29 | [-0.64, 0.04] | 0.956 |
| Urbanicity score (z) | 0.08 | [0.05, 0.11] | 1 | 0.2 | [0.18, 0.23] | 1 | -0.06 | [-0.09, -0.04] | 1 | -0.03 | [-0.05, 0] | 0.974 | 0.91 | [0.83, 0.99] | 1 | -0.1 | [-0.16, -0.03] | 0.999 | 0.34 | [0.23, 0.45] | 1 | 0.39 | [0.22, 0.56] | 1 |
| Vigorous activity (z) | 0.1 | [0.07, 0.13] | 1 | -0.07 | [-0.1, -0.05] | 1 | -0.01 | [-0.04, 0.02] | 0.783 | -0.07 | [-0.1, -0.04] | 1 | -0.37 | [-0.46, -0.28] | 1 | -0.16 | [-0.22, -0.09] | 1 | -0.2 | [-0.32, -0.08] | 1 | -0.1 | [-0.3, 0.09] | 0.84 |

*Pr(direction) describes the proportion of the posterior that falls on the same side of 0 as the main association (i.e., a value of 0.96 can be interpreted as expressing 96% confidence that a given parameter is directionally greater or less than zero).

### Physical activity predictor: ENMO; adjusted for urbanicity

| | Weight | | | Body fat | | | Waist circumference | | | Waist:hip ratio | | | BMI | | | BRI | | | Total cholesterol | | | LDL | | |
|---|---|---|---|---|---|---|---|---|---|---|---|---|---|---|---|---|---|---|---|---|---|---|---|---|
| Predictor | Estimate | 95% CrI | Pr(direction) | Estimate | 95% CrI | Pr(direction) | Estimate | 95% CrI | Pr(direction) | Estimate | 95% CrI | Pr(direction) | Estimate | 95% CrI | Pr(direction) | Estimate | 95% CrI | Pr(direction) | Estimate | 95% CrI | Pr(direction) | Estimate | 95% CrI | Pr(direction) |
| Intercept | -0.06 | [-0.09, -0.02] | 1 | 0.47 | [0.44, 0.5] | 1 | 0.1 | [0.07, 0.14] | 1 | -0.05 | [-0.09, -0.02] | 1 | 0.15 | [0.12, 0.18] | 1 | 0.25 | [0.22, 0.29] | 1 | 0.12 | [0.08, 0.15] | 1 | 0.15 | [0.11, 0.19] | 1 |
| Age (z) | -0.25 | [-0.28, -0.21] | 1 | -0.06 | [-0.09, -0.03] | 1 | 0.03 | [0, 0.07] | 0.968 | 0.24 | [0.2, 0.27] | 1 | -0.17 | [-0.2, -0.14] | 1 | 0.12 | [0.09, 0.16] | 1 | 0.22 | [0.19, 0.25] | 1 | 0.17 | [0.13, 0.21] | 1 |
| Age (z):Sex | 0 | [-0.04, 0.05] | 0.566 | 0.1 | [0.05, 0.14] | 1 | 0 | [-0.05, 0.05] | 0.577 | -0.03 | [-0.08, 0.02] | 0.868 | 0.06 | [0.01, 0.1] | 0.987 | -0.01 | [-0.06, 0.04] | 0.673 | -0.21 | [-0.26, -0.15] | 1 | -0.12 | [-0.17, -0.06] | 1 |
| Sex | 0.3 | [0.25, 0.35] | 1 | -1.06 | [-1.11, -1.02] | 1 | -0.11 | [-0.16, -0.05] | 1 | 0.31 | [0.25, 0.37] | 1 | -0.23 | [-0.28, -0.18] | 1 | -0.48 | [-0.54, -0.43] | 1 | -0.19 | [-0.26, -0.14] | 1 | -0.26 | [-0.32, -0.2] | 1 |
| Smoking (y/n) | -0.15 | [-0.2, -0.09] | 1 | -0.26 | [-0.31, -0.22] | 1 | -0.22 | [-0.27, -0.16] | 1 | -0.21 | [-0.27, -0.15] | 1 | -0.2 | [-0.25, -0.14] | 1 | -0.25 | [-0.3, -0.2] | 1 | -0.11 | [-0.17, -0.06] | 1 | -0.17 | [-0.23, -0.11] | 1 |
| Urbanicity score (z) | 0.42 | [0.4, 0.45] | 1 | 0.26 | [0.24, 0.29] | 1 | 0.33 | [0.31, 0.36] | 1 | 0.05 | [0.02, 0.07] | 1 | 0.38 | [0.36, 0.41] | 1 | 0.28 | [0.25, 0.3] | 1 | 0.1 | [0.08, 0.13] | 1 | 0.04 | [0.01, 0.06] | 0.993 |
| ENMO (z) | -0.1 | [-0.13, -0.08] | 1 | -0.11 | [-0.13, -0.08] | 1 | -0.1 | [-0.13, -0.07] | 1 | -0.05 | [-0.08, -0.02] | 1 | -0.09 | [-0.12, -0.06] | 1 | -0.09 | [-0.12, -0.06] | 1 | -0.07 | [-0.1, -0.05] | 1 | -0.08 | [-0.11, -0.05] | 1 |

| | HDL | | | Triglycerides | | | Systolic BP | | | Diastolic BP | | | Obese (y/n) | | | Hypertension (y/n) | | | Pre-diabetes (y/n) | | | Diabetes (y/n) | | |
|---|---|---|---|---|---|---|---|---|---|---|---|---|---|---|---|---|---|---|---|---|---|---|---|---|
| Predictor | Estimate | 95% CrI | Pr(direction) | Estimate | 95% CrI | Pr(direction) | Estimate | 95% CrI | Pr(direction) | Estimate | 95% CrI | Pr(direction) | Estimate | 95% CrI | Pr(direction) | Estimate | 95% CrI | Pr(direction) | Estimate | 95% CrI | Pr(direction) | Estimate | 95% CrI | Pr(direction) |
| Intercept | 0.21 | [0.18, 0.25] | 1 | -0.23 | [-0.26, -0.2] | 1 | -0.04 | [-0.07, -0.01] | 0.997 | 0.03 | [-0.01, 0.06] | 0.923 | -1.34 | [-1.44, -1.25] | 1 | 0.14 | [0.06, 0.22] | 1 | -2.46 | [-2.59, -2.33] | 1 | -3.65 | [-3.89, -3.42] | 1 |
| Age (z) | 0.15 | [0.12, 0.19] | 1 | 0.1 | [0.06, 0.13] | 1 | 0.54 | [0.51, 0.58] | 1 | 0.29 | [0.26, 0.33] | 1 | -0.4 | [-0.5, -0.31] | 1 | 0.71 | [0.63, 0.79] | 1 | 0.47 | [0.35, 0.6] | 1 | 0.57 | [0.38, 0.76] | 1 |
| Age (z):Sex | 0.06 | [0, 0.11] | 0.982 | -0.16 | [-0.22, -0.11] | 1 | -0.31 | [-0.36, -0.26] | 1 | -0.14 | [-0.2, -0.09] | 1 | 0.09 | [-0.08, 0.26] | 0.849 | -0.29 | [-0.41, -0.17] | 1 | -0.08 | [-0.28, 0.11] | 0.786 | -0.35 | [-0.65, -0.04] | 0.989 |
| Sex | -0.42 | [-0.48, -0.36] | 1 | 0.43 | [0.37, 0.49] | 1 | 0.53 | [0.48, 0.59] | 1 | 0.19 | [0.13, 0.25] | 1 | -0.7 | [-0.9, -0.51] | 1 | 0.59 | [0.46, 0.72] | 1 | -0.15 | [-0.4, 0.1] | 0.867 | 0.11 | [-0.28, 0.51] | 0.712 |
| Smoking (y/n) | -0.2 | [-0.25, -0.14] | 1 | 0.19 | [0.14, 0.25] | 1 | -0.35 | [-0.4, -0.3] | 1 | -0.25 | [-0.31, -0.2] | 1 | -0.6 | [-0.78, -0.42] | 1 | -0.67 | [-0.8, -0.53] | 1 | -0.33 | [-0.56, -0.1] | 0.997 | -0.28 | [-0.64, 0.07] | 0.938 |
| Urbanicity score (z) | 0.07 | [0.04, 0.1] | 1 | 0.2 | [0.18, 0.23] | 1 | -0.06 | [-0.09, -0.04] | 1 | -0.02 | [-0.04, 0.01] | 0.897 | 0.93 | [0.85, 1.01] | 1 | -0.08 | [-0.14, -0.02] | 0.995 | 0.36 | [0.26, 0.46] | 1 | 0.38 | [0.2, 0.54] | 1 |
| ENMO (z) | 0.05 | [0.02, 0.07] | 1 | -0.09 | [-0.12, -0.06] | 1 | -0.03 | [-0.06, -0.01] | 0.996 | -0.08 | [-0.11, -0.05] | 1 | -0.44 | [-0.53, -0.35] | 1 | -0.2 | [-0.26, -0.13] | 1 | -0.12 | [-0.23, -0.01] | 0.98 | -0.13 | [-0.32, 0.05] | 0.922 |

*Pr(direction) describes the proportion of the posterior that falls on the same side of 0 as the main association (i.e., a value of 0.96 can be interpreted as expressing 96% confidence that a given parameter is directionally greater or less than zero).

### Physical activity predictor: DFA; adjusted for urbanicity

| | Weight | | | Body fat | | | Waist circumference | | | Waist:hip ratio | | | BMI | | | BRI | | | Total cholesterol | | | LDL | | |
|---|---|---|---|---|---|---|---|---|---|---|---|---|---|---|---|---|---|---|---|---|---|---|---|---|
| Predictor | Estimate | 95% CrI | Pr(direction) | Estimate | 95% CrI | Pr(direction) | Estimate | 95% CrI | Pr(direction) | Estimate | 95% CrI | Pr(direction) | Estimate | 95% CrI | Pr(direction) | Estimate | 95% CrI | Pr(direction) | Estimate | 95% CrI | Pr(direction) | Estimate | 95% CrI | Pr(direction) |
| Intercept | -0.06 | [-0.09, -0.02] | 0.999 | 0.48 | [0.45, 0.51] | 1 | 0.1 | [0.07, 0.13] | 1 | -0.06 | [-0.09, -0.02] | 0.999 | 0.16 | [0.13, 0.19] | 1 | 0.26 | [0.22, 0.29] | 1 | 0.1 | [0.07, 0.14] | 1 | 0.15 | [0.11, 0.19] | 1 |
| Age (z) | -0.24 | [-0.27, -0.21] | 1 | -0.05 | [-0.08, -0.02] | 1 | 0.03 | [0, 0.07] | 0.978 | 0.24 | [0.2, 0.27] | 1 | -0.16 | [-0.19, -0.13] | 1 | 0.13 | [0.1, 0.16] | 1 | 0.22 | [0.18, 0.25] | 1 | 0.17 | [0.14, 0.21] | 1 |
| Age (z):Sex | 0.03 | [-0.02, 0.08] | 0.87 | 0.12 | [0.08, 0.16] | 1 | 0.02 | [-0.02, 0.07] | 0.841 | -0.02 | [-0.07, 0.03] | 0.777 | 0.08 | [0.03, 0.12] | 0.999 | 0.01 | [-0.04, 0.06] | 0.676 | -0.19 | [-0.24, -0.14] | 1 | -0.1 | [-0.15, -0.04] | 1 |
| Sex | 0.29 | [0.24, 0.35] | 1 | -1.11 | [-1.15, -1.06] | 1 | -0.1 | [-0.16, -0.04] | 1 | 0.32 | [0.26, 0.38] | 1 | -0.26 | [-0.31, -0.2] | 1 | -0.5 | [-0.55, -0.44] | 1 | -0.18 | [-0.24, -0.12] | 1 | -0.27 | [-0.33, -0.21] | 1 |
| Smoking (y/n) | -0.14 | [-0.19, -0.08] | 1 | -0.26 | [-0.31, -0.21] | 1 | -0.21 | [-0.27, -0.15] | 1 | -0.2 | [-0.26, -0.15] | 1 | -0.19 | [-0.24, -0.14] | 1 | -0.24 | [-0.29, -0.18] | 1 | -0.1 | [-0.16, -0.04] | 1 | -0.16 | [-0.22, -0.1] | 1 |
| Urbanicity score (z) | 0.44 | [0.41, 0.46] | 1 | 0.26 | [0.24, 0.29] | 1 | 0.36 | [0.33, 0.38] | 1 | 0.06 | [0.03, 0.09] | 1 | 0.39 | [0.36, 0.41] | 1 | 0.29 | [0.27, 0.32] | 1 | 0.12 | [0.1, 0.15] | 1 | 0.05 | [0.02, 0.08] | 0.997 |
| DFA (z) | 0.02 | [-0.01, 0.05] | 0.944 | -0.03 | [-0.06, -0.01] | 0.996 | 0.04 | [0.01, 0.06] | 0.995 | 0.02 | [-0.01, 0.05] | 0.915 | -0.01 | [-0.03, 0.02] | 0.739 | 0.01 | [-0.02, 0.04] | 0.774 | 0.05 | [0.02, 0.08] | 1 | 0.01 | [-0.02, 0.04] | 0.832 |

| | HDL | | | Triglycerides | | | Systolic BP | | | Diastolic BP | | | Obese (y/n) | | | Hypertension (y/n) | | | Pre-diabetes (y/n) | | | Diabetes (y/n) | | |
|---|---|---|---|---|---|---|---|---|---|---|---|---|---|---|---|---|---|---|---|---|---|---|---|---|
| Predictor | Estimate | 95% CrI | Pr(direction) | Estimate | 95% CrI | Pr(direction) | Estimate | 95% CrI | Pr(direction) | Estimate | 95% CrI | Pr(direction) | Estimate | 95% CrI | Pr(direction) | Estimate | 95% CrI | Pr(direction) | Estimate | 95% CrI | Pr(direction) | Estimate | 95% CrI | Pr(direction) |
| Intercept | 0.2 | [0.16, 0.23] | 1 | -0.22 | [-0.26, -0.19] | 1 | -0.04 | [-0.07, -0.01] | 0.996 | 0.04 | [0, 0.07] | 0.977 | -1.29 | [-1.39, -1.19] | 1 | 0.16 | [0.08, 0.24] | 1 | -2.41 | [-2.55, -2.28] | 1 | -3.63 | [-3.88, -3.4] | 1 |
| Age (z) | 0.14 | [0.1, 0.17] | 1 | 0.1 | [0.07, 0.14] | 1 | 0.54 | [0.51, 0.57] | 1 | 0.31 | [0.27, 0.34] | 1 | -0.36 | [-0.46, -0.26] | 1 | 0.73 | [0.65, 0.81] | 1 | 0.51 | [0.39, 0.64] | 1 | 0.6 | [0.41, 0.79] | 1 |
| Age (z):Sex | 0.05 | [0, 0.1] | 0.968 | -0.14 | [-0.19, -0.09] | 1 | -0.31 | [-0.36, -0.26] | 1 | -0.13 | [-0.18, -0.08] | 1 | 0.18 | [0, 0.36] | 0.975 | -0.26 | [-0.38, -0.14] | 1 | -0.05 | [-0.25, 0.15] | 0.702 | -0.33 | [-0.63, -0.02] | 0.982 |
| Sex | -0.38 | [-0.44, -0.32] | 1 | 0.41 | [0.35, 0.47] | 1 | 0.54 | [0.49, 0.6] | 1 | 0.16 | [0.1, 0.22] | 1 | -0.8 | [-0.99, -0.6] | 1 | 0.52 | [0.39, 0.65] | 1 | -0.29 | [-0.56, -0.02] | 0.983 | 0.05 | [-0.36, 0.44] | 0.601 |
| Smoking (y/n) | -0.19 | [-0.25, -0.14] | 1 | 0.2 | [0.15, 0.26] | 1 | -0.35 | [-0.4, -0.29] | 1 | -0.25 | [-0.31, -0.19] | 1 | -0.53 | [-0.72, -0.35] | 1 | -0.65 | [-0.79, -0.52] | 1 | -0.34 | [-0.57, -0.11] | 0.999 | -0.27 | [-0.64, 0.1] | 0.93 |
| Urbanicity score (z) | 0.08 | [0.06, 0.11] | 1 | 0.21 | [0.18, 0.24] | 1 | -0.05 | [-0.08, -0.03] | 1 | -0.02 | [-0.05, 0.01] | 0.904 | 0.93 | [0.85, 1.02] | 1 | -0.08 | [-0.14, -0.01] | 0.992 | 0.31 | [0.19, 0.43] | 1 | 0.37 | [0.19, 0.55] | 1 |
| DFA (z) | 0.05 | [0.02, 0.07] | 1 | 0 | [-0.03, 0.03] | 0.507 | 0.02 | [-0.01, 0.04] | 0.914 | -0.03 | [-0.05, 0] | 0.957 | -0.06 | [-0.14, 0.02] | 0.919 | -0.04 | [-0.11, 0.02] | 0.916 | -0.19 | [-0.3, -0.08] | 1 | -0.06 | [-0.23, 0.11] | 0.77 |

*Pr(direction) describes the proportion of the posterior that falls on the same side of 0 as the main association (i.e., a value of 0.96 can be interpreted as expressing 96% confidence that a given parameter is directionally greater or less than zero).
